# The Effect of Selenium-Based Ligands on Tungsten Acetylene Complexes

**DOI:** 10.1021/acs.inorgchem.4c01636

**Published:** 2024-06-20

**Authors:** Lorenz Steiner, Antoine Dupé, Karl Kirchner, Nadia C. Mösch-Zanetti

**Affiliations:** † Institute of Chemistry, Inorganic Chemistry, University of Graz, 8010 Graz, Austria; ‡ Institute of Applied Synthetic Chemistry, Vienna University of Technology, 1060 Vienna, Austria

## Abstract

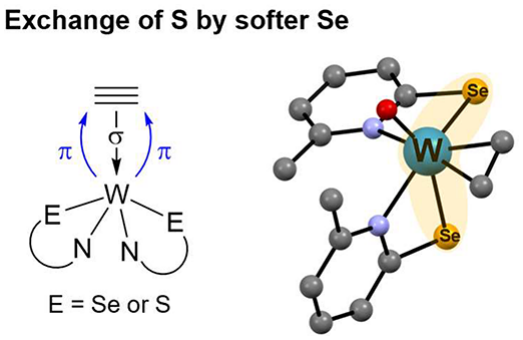

Bioinspired tungsten acetylene complexes containing pyridine-2-selenolato (PySe) or 6-methyl-pyridine-2-selenolato (6-MePySe) ligands were synthesized. ^77^Se NMR spectroscopy allowed for an assessment of the resonance structures in the pyridine-2-selenolato ligands and the rationalization of chemoselectivity observed in regard to 1,2 migratory insertion of HC≡CH. [W(CO)(C_2_H_2_)(CHCH-PySe)(PySe)] is formed exclusively via insertion of HC≡CH into the W–N bond, while the use of bulkier 6-MePySe allows for the isolation of [W(CO)(C_2_H_2_)(6-MePySe)_2_], which only partially reacts with excess HC≡CH to give [W(CO)(C_2_H_2_)(CHCH-6-MePySe)(6-MePySe)]. Oxidation of [W(CO)(C_2_H_2_)(6-MePySe)_2_] with pyridine-*N*-oxide gave the tungsten(IV) complex [WO(C_2_H_2_)(6-MePySe)_2_]. Complexes [W(CO)(C_2_H_2_)(6-MePySe)_2_] and [WO(C_2_H_2_)(6-MePySe)_2_] react with trimethyl phosphine to carbyne complex [W(CO)(CCH_2_PMe_3_)(PMe_3_)_2_(6-MePySe)]Cl and alkylidene complex [WO(CHCHPMe_3_)(PMe_3_)_2_(6-MePySe)]Cl, respectively. The addition of substituted alkynes to [W(CO)_3_(PySe)_2_] via thermal decarbonylation gave complexes [W(CO)(MeC≡CMe)(PySe)_2_] and [W(CO)(HC≡C*t*-Bu)(PySe)_2_], respectively. The here presented complexes are relevant for the modeling of the active site of acetylene hydratase from *Pelobacter acetylenicus*, in which a tungsten atom is enclosed in a sulfur-rich coordination sphere. A recently published theoretical study concluded that the exchange of sulfur for selenium would increase the activity of the enzyme. Our findings contrast this claim as comparative analysis concludes negligible structural and electronic differences between the selenium-based and previously published sulfur-based complexes.

## Abstract 2

Bioinspired pyridine-2-selenolato tungsten acetylene complexes were prepared and characterized, and a comparative analysis vs analogous pyridine-2-thiolato tungsten complexes revealed the electron-donating influence of the softer selenium over sulfur to be small which is relevant when cysteine is exchanged for selenocysteine.

## Introduction

Tungsten, the heaviest metal in biology, is found in several bacterial or archaeal tungstoenzymes. They catalyze various oxygen atom transfer reactions, similar to ubiquitous molybdoenzymes. These enzymes all contain an active site consisting of a metal atom, W or Mo, which is coordinated to the dithiolene group of pterin cofactors (metallopterin or metal binding pterin; MPT). Among them, the tungstoenzyme acetylene hydratase (AH) from *Pelobacter acetylenicus* is unique as it catalyzes a nonredox reaction, namely the hydration of acetylene to acetaldehyde.^1,2^ The active site consists of a tungsten(IV) center coordinated by two MPT cofactors,^3^ one cysteine, and one oxygen atom, most likely from a molecule of water, while the substrate acetylene has not been detected. For this reason, the mechanism is still under debate attracting several theoretical investigations.^4−7^ Proposed mechanisms fall into two categories: In the first shell mechanism, acetylene is coordinated at W and thus activated and attacked by incoming water.^1^ In the second shell mechanism, water is activated at W which attacks incoming acetylene.^4,5^

In the first shell mechanism with an external nucleophilic attack on the coordinated acetylene, the electronic properties of the metal center play a crucial role in the feasibility of this reaction. In the case of olefin metal complexes an external nucleophilic attack on coordinated olefin is expected when the metal is a good σ-acceptor while exhibiting low π-basicity.^8^ However, a theoretical study finds Au(I) alkyne complexes, which are intermediates in nucleophilic attacks on alkynes, to exhibit a strong π-backdonation.^9^

Recently, Zhang and Bushnell performed a quantum mechanical/molecular mechanical investigation of the first shell mechanism of AH.^10^ Their comparative study of the energies of AH with the native dithiolene environment and a model in which the sulfur atoms are exchanged for selenium showed that Se makes binding of acetylene less endergonic by 65.6 kJ/mol and the overall reaction less endergonic by 38.2 kJ/mol.^10^ Hence, exchanging S for Se would lead to a more active enzyme. To investigate the influence of selenium vs sulfur ligands on the coordination and conversion of acetylene we developed bioinspired tungsten complexes with selenium ligands for comparison to our previously described sulfur-based models.^11−15^

According to the Dewar-Chatt-Duncanson bonding model, metal to ligand π-backdonation occurs into the π*-orbital at the alkyne. In the case of selenium vs sulfur, the softer selenium is expected to increase the electron density at tungsten leading to a more pronounced π-backdonation and hence to a more activated acetylene with a longer C≡C bond.

Bioinorganic model chemistry of tungstoenzymes has been developed using dithiolene and nondithiolene ligands.^16−20^ However, models that address specifically mechanistic aspects of AH are scarce. Sarkar and co-workers reported that the W(IV) complex Et_4_N[WO(mnt)_2_] (mnt = maleonitriledithiolate) is an active catalyst for the conversion of acetylene to acetaldehyde,^21^ however the results were later disputed by a study of Hintermann and co-workers.^22^

**Figure 1:**
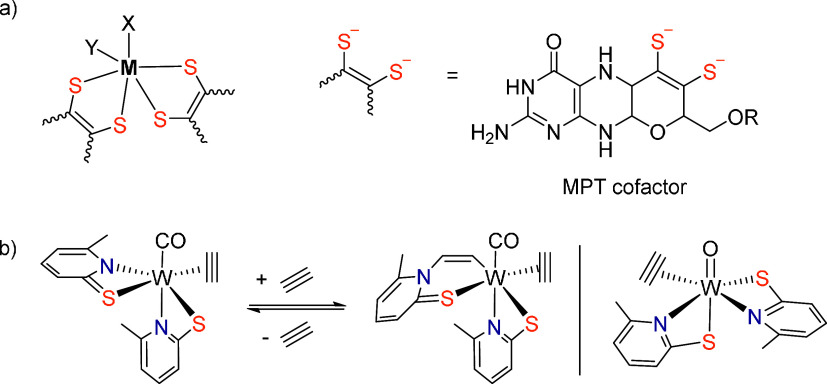
(a) Structural motif of the active site found in DMSO reductase family (M = Mo; X = serine, cysteine, selenocysteine; Y = oxygen) and acetylene hydratase (M = W; X = cysteine; Y = OH or H_2_O, R = guanosine diphosphate). (b) Previously published tungsten(II) and tungsten(IV) acetylene complexes derived from pyridine-2-thiolate ligands.^14^

Our group has investigated Mo^23^ as well as W acetylene complexes coordinated by S-containing ligands as potential models for AH, i.e. thiophenyloxazoline,^11,12^ pyridine-2-thiolates,^13−15^ and hydridotris(2-mercapto-1-methylimidazolyl) borate).^24^ Acetylene was found to insert into the W–N bond of the pyridine-2-thiolato ligand in W(II) complexes, e.g. of the type [W(PyS)_2_(C_2_H_2_)(CO)] (PyS = pyridine-2-thiolate) forming [W(CO)(C_2_H_2_)(CHCHPyS)(PyS)] (Figure 1).^13,15^ This represents an intramolecular nucleophilic attack on acetylene. By introducing various substituents in the pyridine-2-thiolato ligands we found insertion to be favored with electron rich ligands.^15^ Thus, in [W(CO)(C_2_H_2_)(CHCHPyS)(PyS)] the insertion is reversible in contrast to [W(CO)(C_2_H_2_)(CHCH-4-MePys)(4-MePyS)] (4-Me-PyS = 4-methyl-pyridine-2-thiolate) where the insertion is irreversible. It is furthermore crucial that the 6-position of the pyridine-2-thiolate is not blocked by bulky substituents as otherwise insertion is also hindered.^15^

Intermolecular nucleophilic attack of water on coordinated acetylene has not been observed in these systems. However, with the W acetylene complexes [W(CO)(C_2_H_2_)(6-MePyS)_2_] and [WO(C_2_H_2_)(6-MePyS)_2_] (6-MePyS = 6-methyl-pyridine-2-thiolate), respectively, the nucleophilic attack of trimethyl phosphine on coordinated acetylene was observed to give a carbyne in the former, and an alkenyl complex in the latter.^14^

While the chemistry of transition metal complexes containing pyridine-2-thiolato ligands has been widely explored,^25^ examples of analogous pyridine-2-selenolato ligands are much less common. Much like its pyridine-2-thiolate analog, the soft Se and hard N in pyridine-2-selenolates allow for bidentate coordination to a broad spectrum of transition metals. Mononuclear complexes of pyridine-2-selenolates are known for a variety of transition metals including Re,^26,27^ In,^28−30^ Ru,^31^ Os,^31^ Co,^32^ Pd,^33^ Pt,^33^ Ni,^34^ and others. Group 6 transition metals are only sparingly explored and to our knowledge, just two molybdenum complexes,^35,36^ and one example of a tungsten complex are known in literature.^35^ The naturally occurring isotope ^77^Se with its nuclear spin quantum number of 1/2 renders it a highly useful NMR probe.^37^ With the selenium ligands used in this study, ^77^Se NMR spectroscopy is particularly useful as it allows distinguishing between the two mesomeric resonance structures pyridine-2-selenolate vs -selenone (Scheme 5) due to their different charge at Se.^37,38^

Here, we present the preparation of a series of W complexes that contain the pyridine-selenolato ligands PySe and 6-MePySe which are analogous to our previously reported pyridine-thiolato ligands allowing for a direct chemical, structural, and spectroscopic comparison and assessment of the effect that Se has in these complexes.

## Results and Discussion

### Ligand Synthesis

Pyridine-2-selenol (PySeH) and 6-methyl-pyridine-2-selenol (6-MePySeH) were prepared from a modified literature procedure by reaction of 2-chloro-pyridine or 2-chloro-6-methyl-pyridine, respectively, with Na_2_Se_2_, which was freshly prepared from selenium metal and NaBH_4_ in degassed water and under exclusion of O_2_.^39^ The two selenols PySeH and 6-MePySeH, respectively, were isolated as bright yellow powders.

Under ambient conditions, PySeH and 6-MePySeH in solution are rapidly oxidized to the corresponding diselenide. Solid samples are also oxidized over several hours after which a sticky material is obtained, likely caused by the formation of H_2_O in the process. The sodium salts Na(PySe) and Na(6-MePySe), respectively, were prepared from the corresponding selenols by the addition of NaH in THF and were isolated as off-white solids, that are not hygroscopic and could be handled under ambient conditions for a short time without any apparent decomposition. Alternatively, the sodium salts can also be prepared *in situ* from the corresponding diselenide in methanol with 2 equiv of NaBH_4_.

### Preparation of Tungsten Compounds

**Scheme 1:**
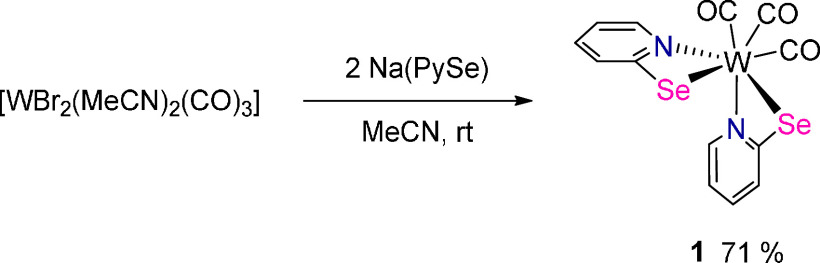
Synthesis of [W(CO)_3_(PySe)_2_] (**1**) by Salt Metathesis

Reaction of [WBr_2_(MeCN)_2_(CO)_3_] with 2.1 equiv of Na(PySe) in acetonitrile and workup as described in the Experimental Section gave the tungsten carbonyl compound [W(CO)_3_(PySe)_2_] (**1**) as a red microcrystalline solid in 71% yield (Scheme 1). The compound is stable in the solid state under ambient conditions. It is soluble in dichloromethane, chloroform, tetrahydrofuran, benzene, and toluene, but insoluble in methanol, acetonitrile, and water. The synthetic procedure is similar to that of our previously described sulfur analog [W(CO)_3_(PyS)_2_].^14^ However, we obtained higher yields by using acetonitrile compared to dichloromethane as **1** cleanly precipitates from the reaction solution. The preparation of **1** was previously described by an alternative route by reaction of 2,2′-dipyridyl diselenide with norbornadiene tetracarbonyl tungsten upon oxidation of the metal.^35^ NMR data of **1** are consistent with those reported in literature. In contrast, attempts to obtain the analogous W(II) compound [W(CO)_3_(6-MePySe)_2_] with an additional methyl group in the selenium ligand were futile as the reaction of [WBr_2_(MeCN)_2_(CO)_3_] with Na(6-MePySe) gave only intractable mixtures.

**Scheme 2:**
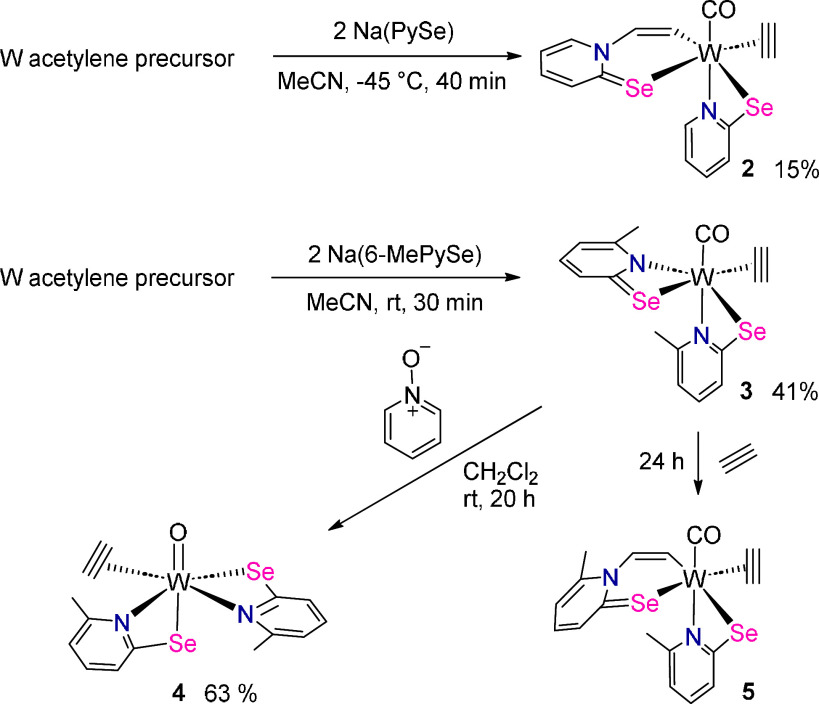
Preparation of **2**, **3**, **4**, and **5** Compound **5** is observed by ^1^H NMR spectroscopy. W acetylene precursor = mixture of 0.5 equiv [WBr_2_(MeCN)_2_(C_2_H_2_)(CO)] and 0.5 equiv of [WBr_2_(MeCN)(C_2_H_2_)_2_(CO)]^13^.

For the preparation of a tungsten acetylene complex, compound **1** was stirred in dichloromethane under an atmosphere of acetylene. However, we were again unable to isolate any product but rather the formation of a black precipitate, presumably polyacetylene, was observed. Also, by addition of smaller amounts of acetylene, no product could be isolated. For these reasons, Na(6-MePySe) and also Na(PySe), respectively, were reacted with a tungsten starting material which contains coordinated acetylene. By applying such a synthetic procedure, we have previously been successful if polyacetylene formation is predominant.^13^ A W(II) acetylene starting material containing halogen ligands which can be substituted by the selenolato ligand is only available as a mixture with one or two coordinated C_2_H_2_ molecules of the type [WBr_2_(MeCN)_2_(C_2_H_2_)(CO)]/[WBr_2_(MeCN)(C_2_H_2_)_2_(CO)] (1:1).^13^ Thus, the latter was reacted with 2 equiv of Na(PySe) and Na(6-MePySe), respectively, in acetonitrile. The reaction employing Na(6-MePySe) allowed the isolation of the tungsten acetylene compound [W(CO)(C_2_H_2_)(6-MePySe)_2_] (**3**) as a fine purple powder in 60% yield (Scheme 2). The bulk material is of suitable purity for use in further reactions without purification. Spectroscopically pure material was obtained by recrystallization from CH_2_Cl_2_/*n*-heptane at −30 °C in 41% overall yield. Compound **3** was found to be soluble in dichloromethane, chloroform, and tetrahydrofuran, slightly soluble in acetonitrile, and insoluble in aliphatic hydrocarbons, diethyl ether as well as toluene. Solid samples are stable under inert conditions, but solutions decompose within days at room temperature.

In contrast, the reaction of Na(PySe) with the tungsten acetylene precursor mixture did not lead to [W(CO)(C_2_H_2_)(PySe)_2_], the analogous compound to **3**, but rather compound [W(CO)(C_2_H_2_)(CHCH-PySe)(PySe)] (**2**) with an additional inserted C_2_H_2_ molecule was isolated as a purple solid. Since only 50% of the precursor mixture contains two molecules of C_2_H_2_, the yield is with 15% vs the total W content inherently low. Solutions of **2** in dichloromethane decomposed within hours after which a reddish-brown solution over a dark precipitate was obtained.

The structure of **2** was confirmed by single crystal X-ray diffraction analysis (*vide infra*, Figure 1). The formation of **2**, and therefore the ease of insertion, is consistent with the reactivity of [W(CO)_3_(PySe)_2_] (**1**) with acetylene where only polyacetylene is observed. In contrast, in [W(CO)(C_2_H_2_)(6-MePySe)_2_] (**3**) insertion is sterically hindered due to the methyl group *ortho* to nitrogen allowing its isolation.

The reaction of **3** with excess acetylene in dichloromethane at room temperature led after 24 h to a 2:1 mixture of **3** and [W(CO)(C_2_H_2_)(CHCH-6-MePySe)(6-MePySe)] (**5**); the latter revealing resonances in the ^1^H NMR spectrum which are comparable to those of [W(CO)(C_2_H_2_)(CHCH-6-MePyS)(6-MePyS)].^14^ Two doublets at 7.62 and 6.78 ppm are indicative of the inserted acetylene (Figure S18). Isolation attempts of **5** by crystallization were futile as only mixtures containing **3** and **5** together with small amounts of another product were obtained. In the absence of acetylene, no interconversion of **3** and **5** is occurring, as evidenced by ^1^H NMR spectroscopy.

Overall, complexes **2** and **3** exhibit properties comparable to their corresponding sulfur-based analogs. In both cases, the stability of the complexes was lower in the selenium-based variants, as they readily decomposed when kept in solution, in the case of **2** even at low temperatures. In contrast to [W(CO)(C_2_H_2_)(CHCH-6-MePyS)(6-MePyS)], the insertion of acetylene is irreversible in case of **5**. Se is softer than S and as such has a larger polarizability volume (α_D_ = 28.9 for Se vs α_D_ = 19.4 for S).^40^ Despite this difference, very similar structural and chemical behavior is observed.

The W(IV) oxido complex [WO(C_2_H_2_)(6-MePySe)_2_] (**4**) was obtained by oxidation of **3** with a slight excess (1.5 equiv) of pyridine-*N*-oxide (PyNO). Upon addition of PyNO to a stirred solution of **3**, the intense purple color gradually faded to give a pale-yellow solution after 20 h. By workup as described in the Experimental Section compound **4** was isolated in 63% yield after crystallization. The pale-yellow powder of **4** is soluble in dichloromethane, chloroform, and tetrahydrofuran, slightly soluble in methanol and acetonitrile but insoluble in aliphatic hydrocarbons, diethyl ether, and toluene. Under inert conditions solid samples of **4** are stable for several months, and at ambient conditions for several hours. Solutions of **4** prepared under inert conditions were stable for several days, while the use of benchtop solvents led to decomposition within minutes pointing toward hydrolytic instability.

### Nucleophilic Attack of Coordinated Acetylene

**Scheme 3:**
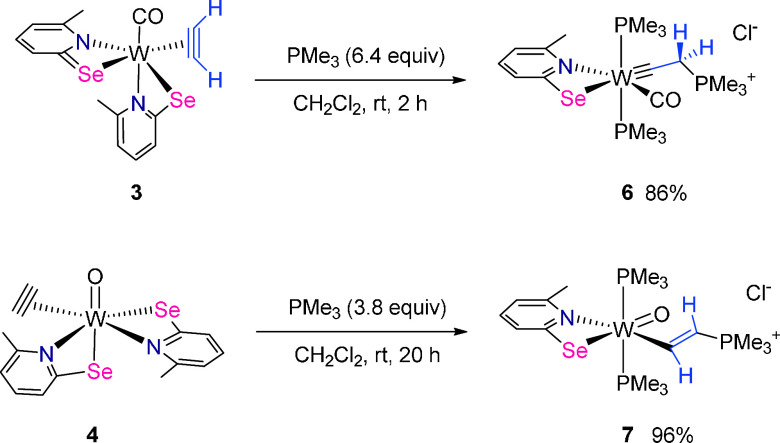
Reaction of η^2^-C_2_H_2_ Complexes **3** and **4** with Excess PMe_3_ Forming **6** and **7**

The reactivity of the W acetylene complexes **3** and **4** toward nucleophilic attack on the coordinated C_2_H_2_ by PMe_3_ was explored (Scheme 3). The reaction of **3** in dichloromethane with excess PMe_3_ (6.4 equiv) led to the formation of the cationic tungsten carbyne complex [W(CO)(CCH_2_PMe_3_)(PMe_3_)_2_(6-MePySe)]Cl (**6**). The product was isolated in 86% yield as an orange powder. The compound is soluble in dichloromethane, but insoluble in aliphatic hydrocarbons, diethyl ether, and toluene. As a solid **6** may be stored for longer periods without apparent change but solutions partly decompose over several days. The phosphine has nucleophilically attacked the coordinated acetylene and under 1,2-hydrogen shift a carbyne complex was formed. The chloride counterion in **6** and **7** originates from the solvent CH_2_Cl_2_. One of the selenolato ligands is removed by reaction with CH_2_Cl_2_ and two additional phosphine molecules coordinate to W. The involvement of CH_2_Cl_2_ in the displacement of the ligand has been previously elaborated for both [W(CO)(CCH_2_PMe_3_)(PMe_3_)_2_(6-Me-Py**S**)]Cl and [WO(CHCHPMe_3_)(PMe_3_)_2_(6-MePyS)]Cl.^14^

The reaction of **4** in CH_2_Cl_2_ with excess PMe_3_ (3.8 equiv) gave the cationic ethenyl complex [WO(CHCHPMe_3_)(PMe_3_)_2_(6-MePySe)]Cl (**7**) in 96% yield. The dark-green complex precipitated upon the addition of toluene. Furthermore, it is insoluble in aliphatic hydrocarbons and diethyl ether, but well soluble in dichloromethane and chloroform. Under inert conditions, solid samples of **7** can be stored without apparent decomposition, but solutions decompose partly within several days. The nucleophilic attack occurred at the η^2^-C_2_H_2_ ligand forming a C–P bond. In contrast to **6**, a single W–C bond remains in **7** making it a tungsten ethenyl complex.

### Preparation of Substituted Alkyne Complexes

**Scheme 4:**
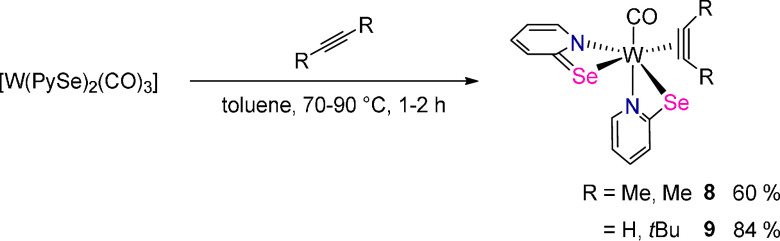
Thermal Decarbonylation in the Presence of Excess Alkyne to Yield **8** and **9**

Since substitution of CO by C_2_H_2_ in [W(CO)_3_(PySe)_2_] (**1**) was not feasible, the reaction of an excess of 2-butyne and 3,3-dimethyl-1-butyne, respectively, with **1** in toluene was investigated. After 1–2 h at 70–80 °C and workup as described in the Experimental Section [W(CO)(MeCCMe)(PySe)_2_] (**8**) and [W(CO)(*t*Bu-CCH)(PySe)_2_] (**9**), respectively, were isolated in good yields as red, crystalline solids (Scheme 4). Solutions of **8** prepared in dichloromethane, benzene or chloroform are intensely green-colored, while solutions in dimethyl sulfoxide or acetonitrile took on a red color. Similarly, solutions of **9** in chloroform or dichloromethane are intensely green-colored.

### NMR Spectroscopy

The ^1^H NMR spectrum of **3** in CD_2_Cl_2_ confirms the coordination of one molecule of C_2_H_2_ (singlets at 13.76 and 12.58 ppm) vs two 6-MePySe ligands. The inequivalence of the two selenolato ligands is confirmed by the observation of two resonances for methyl groups (1.91 and 1.18 ppm). The ^13^C NMR spectrum shows signals at 206.3 and 205.7 ppm which are indicative of the η^2^-C_2_H_2_ acting as a four electron donor.^41^

The ^1^H NMR spectrum of **2** in CD_2_Cl_2_ reveals next to the two singlets at 12.90 and 11.94 ppm, typical for coordinated C_2_H_2_, 10 resonances in the aromatic region consistent with two PySe ligands and one inserted acetylene molecule (Scheme 2).

The ^1^H NMR spectrum of **4** recorded in CD_2_Cl_2_ confirms the coordination of acetylene with the η^2^-C_2_H_2_ protons appearing as singlets at 11.36 and 11.14 ppm, respectively, both flanked by ^183^W satellites. Additionally, the signal at 11.36 ppm is flanked by ^77^Se satellites. In the ^13^C NMR spectrum, the signals for acetylene resonate at 160.9 and 160.6 ppm, respectively.

The formation of **6** is evidenced by the ^1^H NMR spectrum in CD_2_Cl_2_ where the CH_2_ protons appear at 3.77 ppm as doublets of triplets (^2^*J*_HP_ = 19.3 Hz, ^4^*J*_HP_ = 5.2 Hz), and the methyl groups of the two PMe_3_ molecules coordinating W at 1.46 ppm and those of the carbon-bound PMe_3_ at 2.15 ppm, respectively. The ^31^P{^1^H} NMR spectrum of **6** shows a triplet at 20.34 ppm for the carbon-bound PMe_3_ and a doublet at −21.19 ppm being flanked by ^183^W satellites (^1^*J*_WP_ = 278.2 Hz) for the W-bound PMe_3_. In the ^13^C NMR spectrum, the methylene carbon resonates at 45.7 ppm (d, ^1^*J*_CP_ = 49.1 Hz) while the carbyne resonates at 246.2 ppm, which is in the range of similar tungsten carbyne complexes known to literature.^42−44^

The ethenyl protons of **7** are identified by ^1^H NMR spectroscopy with resonances in CD_2_Cl_2_ at 11.49 and 4.26 ppm and coupling to each other with ^3^*J* = 18.1 Hz. The ^31^P{^1^H} NMR spectrum of **7** shows two rather broad singlets at 5.24 and −25.17 ppm, with the latter being flanked with ^183^W satellites. Shifts and coupling constants corresponding to acetylene or PMe_3_, respectively, in the ^1^H and ^31^P NMR spectra of **6** and **7** are virtually identical to the ones found in the analogous pyridine-2-thiolate complexes, [W(CO)(CCH_2_PMe_3_)(PMe_3_)_2_(6-Me-Py**S**)]Cl and [WO(CHCHPMe_3_)(PMe_3_)_2_(6-MePyS)]Cl, respectively, as previously published (Table 1).^14^ The overall comparison of the chemical shifts in the ^1^H NMR and ^31^P NMR spectra suggests that the softer selenium has little influence on the electronic properties of the tungsten center and the structure of the carbyne and vinyl species **6** and **7**.

**Table 1:** ^1^H and ^31^P NMR Spectroscopic Data of [W(CO)(CCH_2_PMe_3_)(PMe_3_)_2_(6-MePy**E**)]Cl and [WO(CHCHPMe_3_)(PMe_3_)_2_(6-Me-Py**E**)]Cl (E = Se, S) Recorded in CD_2_Cl_2_

[W(CO)(CCH_2_PMe_3_)(PMe_3_)_2_(6-Me-Py**E**)]Cl			[WO(CHCHPMe_3_)(PMe_3_)_2_(6-Me-Py**E**)]Cl		
^**1**^**H (ppm)**	E = Se (**6**)	E = S^14^	^**1**^**H (ppm)**	E = Se (**7**)	E = S^14^
CC**H**_2_PMe_3_	3.77	3.81	W–C**H**	11.49	11.42
W(P(C**H**_3_)_3_)_2_	1.46	1.42	W-CC**H**	4.26	4.26
C(P(C**H**_3_)_3_)	2.14	2.12	W(P(C**H**_3_)_3_)_2_	1.43	1.40
			C(P(CH_3_)_3_)	1.94	1.94
					
^**31**^**P (ppm)**	E = Se	E = S^14^	^**31**^**P (ppm)**	E = Se	E = S^14^
W(**P**Me_3_)_2_	–21.19	–17.94	W(**P**Me_3_)_2_	–25.17	–25.17
C-**P**Me_3_	20.34	19.87	C-**P**Me_3_	5.24	5.35

In solution, **8** is present as a mixture of two isomers in the ratio of 2:1 (isomers A:B). The isomers presumably occur due to the relative position of the pyridine-selenolato ligands to each other (*cis*- or *trans*-Se,Se). Also, the ^1^H NMR spectrum of **9** in CDCl_3_ recorded at −30 °C displays four different isomers in the ratio 40:12:4:3 (isomers A:B:C:D) due to the unsymmetric nature of the alkyne.

The ^1^H NMR spectrum of **8** in CDCl_3_ at room temperature reveals two sets of pyridine-2-selenolato ligands while the methyl groups at the alkyne appear as one broad resonance. When the ^1^H NMR spectrum is recorded at −30 °C, the latter resolves into two sets of two singlets also in the ratio of 2:1 which indicates that the alkyne rotates along the bond to tungsten but that the two isomers A and B are not in equilibrium with each other. Our findings are in line with observations from literature.^45^ However, single crystals of **8** were dissolved in CDCl_3_ at −45 °C and a ^1^H NMR spectrum was recorded within 10 min revealing the two isomers A and B in the same ratio 2:1 as observed from bulk samples. Furthermore, upon dissolving a sample in CD_2_Cl_2_ or CD_3_CN the ratio A:B is found to be solvent dependent: 1:1 in the former and 1:2 in the latter. These experimental data point toward an equilibrium of the two isomers which is in contrast to variable temperature ^1^H NMR spectroscopy. Thus, the lack of spectroscopic evidence for the interconversion between the isomers at room temperature is surprising.

**Scheme 5:**
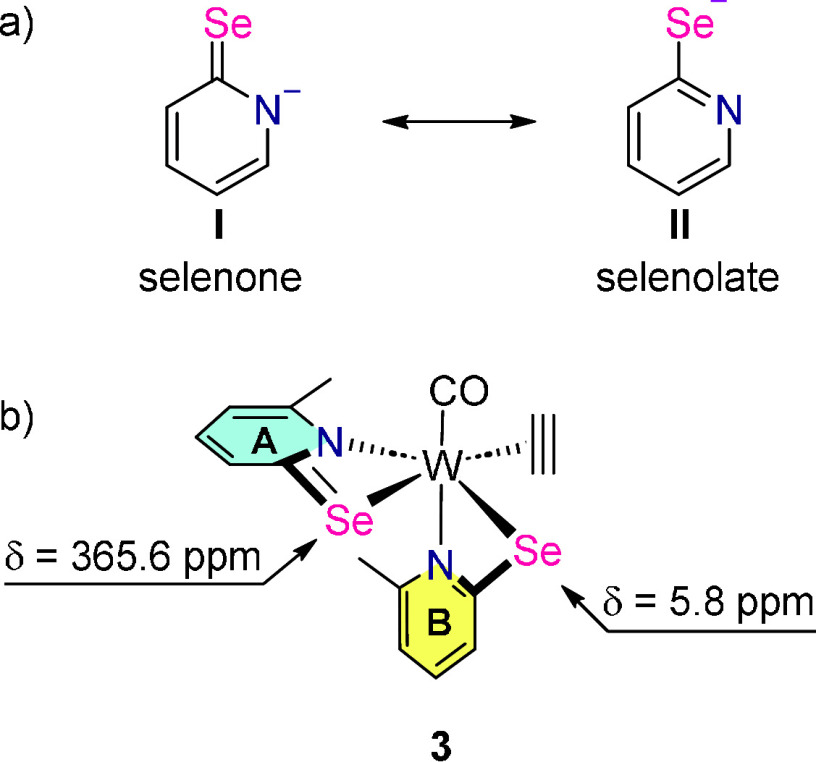
(a) Mesomeric Resonance Structures of the Pyridine-Selenolate Anion. (b) Complex **3** with ^77^Se NMR Data. Ligand A showing downfield shift points towards higher population of the selenone form while upfield shifted ligand B of the selenolate form.

The presence of selenium atoms allows for additional probing by ^77^Se NMR spectroscopy. The high atomic number of selenium requires the consideration of both the diamagnetic, as well as the paramagnetic term when inferring information from observed chemical shifts. Under the justified assumption that the diamagnetic shift term outweighs the paramagnetic one, lower electron density at selenium leads to deshielding and thus to higher ppm values.^37^ The large chemical shift range in ^77^Se NMR spectroscopy (about 2000 to −1000 ppm) may result in a significant difference in the absolute chemical shift of selenium atoms in similar chemical environments, allowing the assessment of electronic trends within similar systems. This is an opportunity to gain insight into the electronic situation of two spectroscopically different but chemically identical ligands in one complex. This is particularly interesting regarding the investigated pyridine-2-selenolato ligands here, since they may exhibit two different mesomeric resonance structures with the negative charge at nitrogen (selenone, form **I** in Scheme 5) or at selenium (selenolate, form **II** in Scheme 5), respectively.

In the selenone form with a C=Se bond, the selenium is less shielded compared to C–Se−, hence higher ppm values point toward a higher population of the selenone form. In the complexes presented here, the availability of the nitrogen lone pair as well as the ability of selenium to stabilize the negative charge associated with resonance form **II** has a profound effect on the chemical shift observed in the ^77^Se NMR spectrum, which is in line with established theories regarding α-hetero substituted selenones.^37^

The ^77^Se NMR spectrum of [W(CO)_3_(PySe)_2_] (**1**) in CDCl_3_ shows a single signal at 61.3 ppm indicative of two selenolate ligands in an identical chemical environment. Complex **3** reveals two resonances at 365.6 and 5.8 ppm in CD_2_Cl_2_ (Scheme 5 and Table 2). This large difference indicates that the two ligands exhibit different relative importance of resonance forms **I** and **II**, respectively, with the downfield signal being consistent with the selenone form (ligand B in Scheme 5). The CO ligand exhibits a strong *trans*-effect on the nitrogen of ligand B, and in turn, its lone pair is available for π-backdonation to the selenium atom. This gives it an increased form **II** mesomeric resonance character and shields it from the magnetic field which results in a notable upfield shift in the ^77^Se NMR spectrum. Similarly, the π-accepting acetylene ligand *trans* to the selenium atom of ligand **A** contributes to its deshielding and thus results in a significantly larger chemical shift. These observations are in accordance with measured bond lengths–in ligand **A** the C–Se bond is shorter while the W–Se bond length is longer in comparison to ligand **B** (see Table 3). Furthermore, these insights allow for the rationalization of the chemoselectivity observed for acetylene insertion into the W–N bond of ligand **A** over ligand **B**, since insertion into the metal–ligand bond only occurs with formally anionic ligands. The relative importane of the resonance form **I** is larger in ligand **A** and as such, insertion may occur. This selectivity has been reported before in complexes of the type [W(CO)(C_2_H_2_)(R-PyS)_2_] (R-PyS = substituted pyridine-2-thiolate).^15^

**Table 2:** Resonances Recorded for Complexes **1**, **3**, **4**, **6**, **8**, and **9** in the ^77^Se NMR Spectrum

complex (solvent)	δ (ppm)
**1** (CDCl_3_)	61.3
**3** (CD_2_Cl_2_)	365.6, 5.8
**4** (CD_2_Cl_2_)	280.4, 176.7
**6** (CD_2_Cl_2_)	–86.1
**8** (CDCl_3_)	295.7, −80.4 (isomer A); 334.8, 45.4 (isomer B)
**9** (CDCl_3_)	303.4, −86.3 (isomer A); 330.0, 52.4 (isomer B)

**Table 3:** Selected Bond Lengths (Å) for Complexes **2**, **3**, and **4** and Their Respective Pyridine-2-thiolate Analogs

	[W(CO)(C_2_H_2_)(Py**E**) (CHCHPy**E**)]		[W(CO)(C_2_H_2_) (6-MePy**E**)_2_]		[WO(C_2_H_2_)(6-MePy**E**)_2_]	
	E = Se (**2**)	E = S^14^	E = Se (**3**)	E = S^13^	E = Se (**4**)	E = S^14^
C1–C2	1.319(8)	1.3148(19)	1.305(6)	1.306(7)	1.280(4)	1.279(2)
W1–E1	2.6683(6)	2.5551(3)	2.6929(5)	2.5834(12)	2.5285(3)	2.4177(4)
W1–E2	2.5919(6)	2.4816(3)	2.5184(5)	2.4073(12)	2.7429(2)	2.6367(5)
W1–N11	–	–	2.215(3)	2.197(3)	2.293(2)	2.2637(13)
W1–N21	2.240(4)	2.2348(10)	2.268(3)	2.259(4)	2.216(2)	2.1971(13)
E1–C12	1.876(5)	1.7183(13)	1.889(4)	1.739(6)	1.893(3)	1.7551(18)
E2–C22	1.909(5)	1.7507(12)	1.907(4)	1.760(6)	1.879(3)	1.7253(16)
W1–C18	2.113(6)	2.0964(13)	–	–	–	–
C17–C18	1.322(8)	1.3421(18)	–	–	–	–
W1–C3	1.973(4)	1.9781(12)	1.973(5)	1.958(5)	–	–
C3–-O3	1.159(7)	1.1611(15)	1.149(5)	1.167(6)	–	–
W–C1	2.040(5)	2.0353(13)	2.022(4)	2.022(5)	2.075(3)	2.0693(15)
W–C2	2.062(6)	2.0582(13)	2.045(4)	2.055(3)	2.088(3)	2.1027(15)

**Figure 2:**
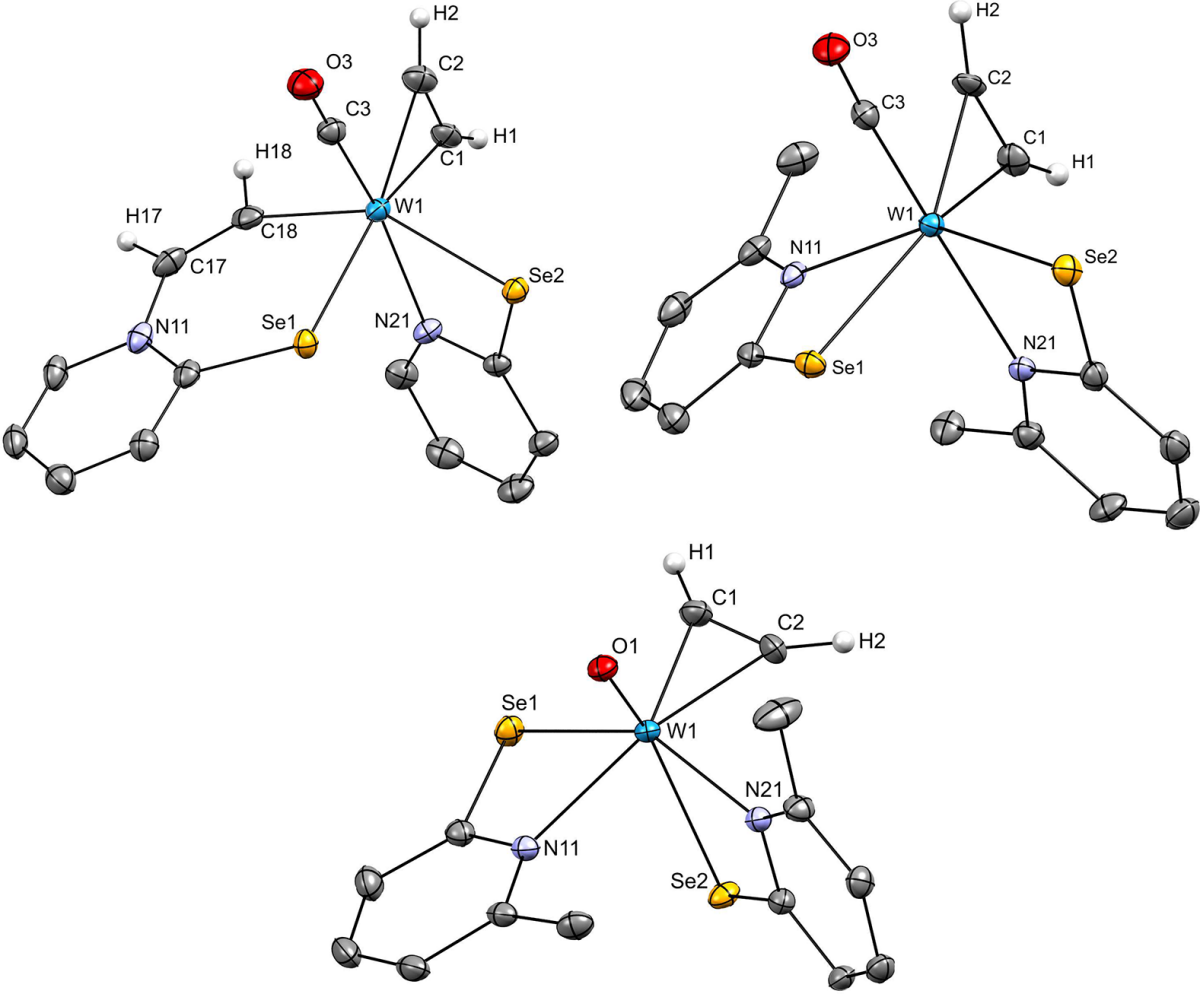
Molecular structures of **2** (top left), **3** (top right), and **4** (bottom) with probability ellipsoids drawn at 50% level. Hydrogen atoms other than acetylene-bound ones are omitted for clarity.

In the ^1^H NMR spectrum of **4** in CD_2_Cl_2_, a set of satellites flank the acetylene signal at 11.36 ppm originating from coupling with ^77^Se (^3^*J*_SeH_ = 6.10 Hz). The coupling is also detected in the corresponding ^77^Se NMR spectrum as a doublet at 280.4 ppm, while the signal corresponding to the other selenium resonates as a singlet at 176.7 ppm (Figure S21). The chemical shift is more indicative of a larger relative population of the mesomeric form **II**, albeit less pronounced in comparison to ligand B in **3**.^38^ The difference in chemical shift and C–Se bond length overall is much less pronounced, likely as a consequence of both selenium atoms oriented *cis* to the coordinated acetylene, and the lack of a carbonyl ligand exhibiting a strong *trans* effect. The fact that only one of the protons shows J-coupling with one of the selenium atoms may be a result of dihedral bond angle overlap as described by the Karplus equation. Hence, in this specific case, we most likely observe a coupling between Se1 and H1 (see Figure 2).^37^

The ^77^Se NMR spectrum of **6** in CD_2_Cl_2_ shows one resonance at −86.1 ppm, which is indicative of the ligand having a notable selenolate character (form **I** in Scheme 5). The cumulative effects of the electron-rich phosphine ligands and the strong *trans*-effect exhibited on the pyridine-2-selenolato ligand by both the carbyne and carbonyl are likely the reason for the low shift in the ^77^Se NMR of **6**.

As discussed above, the 2-butyne complex **8** forms a mixture of two isomers A and B in solution, the structure of which is elusive. From the relative intensity of the signal in the ^77^Se NMR, shifts can be assigned to the corresponding isomer, and shifts are again indicative of two mesomeric forms for each isomer (isomer A: δ = 295.7, – 80.4 ppm; isomer B: 334.8, 45.4 ppm). The very high upfield shifted signal at −80.4 ppm as well as the signal at 45.4 ppm indicate the mesomeric selenolate form **II**, while the shifts at 295.7 and 334.8 ppm correspond to the mesomeric selenone form **I**. The low resonance at −80.4 ppm may arise from the presumed conformational isomers suggested for **8**. Thus, the π-acidic properties of 2-butyne *trans* to selenium would aid in transferring electrons from the aromatic ring to selenium, which in turn leads to significant shielding.

At room temperature, no signals are found in the ^77^Se NMR spectrum of **9** as a result of the rate of rotation of the 2,2-dimethyl-3-butyne ligand. At–20 °C, two sets of signals are detected for each of the major isomers at 303.4 and −86.3 ppm (isomer A), and at 330.0 and 52.4 ppm (isomer B). The similar shift range of these isomers suggests the underlying isomerism is the same as in **8**. The remaining isomers C and D, where the *t*Bu-moiety is pointing toward the CO-ligand, are not resolved due to sensitivity limitations and their inherently low concentration in solution.

Although the amide character is clearly given in these cases, we did not observe the insertion of higher alkynes into the W–N bond. This selectivity is a parallel to AH which was found to not convert propyne, *p*-toluyl-acetylene, acetylene dicarboxylate, or propiolic acid to the corresponding aldehydes or ketones, but rather these alkynes act as inhibitors for enzyme activity.^46^

### Molecular Structures

The structures of acetylene complexes **2**, **3,** and **4** were determined by single crystal X-ray diffraction analyses, and molecular views are displayed in Figure 2. Suitable single crystals were obtained as described in the Experimental Section. Crystallographic data can be found in the Supporting Information and selected bond lengths are summarized in Table 3. All three compounds contain two pyridine-selenolato ligands and one η^2^-coordinate acetylene molecule. In **2**, a second acetylene has inserted into one tungsten nitrogen bond. Both, **2** and **4** contain one CO ligand while in **4** this has been replaced by an oxido ligand thereby oxidizing the central atom to W(IV). Considering the acetylene ligand as one coordination site, they all exhibit distorted octahedral geometries. All complexes are present as the N,N-*cis*-Se,Se-*cis* geometry.

In both, **2** and **3** the acetylene is *trans* to Se, while CO is *trans* to N. This is reversed in the case of **4**, where the coordinated acetylene is *trans* to N, while O is *trans* to Se. The C≡C bond of coordinated acetylene is aligned in parallel with the W-CO bond in **2** and **3** and perpendicular to the W=O bond in **4**. The three structures are very similar to those of the analogous compounds with sulfur instead of selenium as summarized in Table 3.^13,14^

Comparison of the three selenolato-based compounds reveals that the C1–C2 bond length decreases in the order **2** (1.319(8) Å), **3** (1.305(6) Å) to **4** (1.280(4) Å). In **2**, the bond length of the inserted acetylene was measured at C17–C18 (1.322(8) Å). The coordinated acetylenes in the W(II) complexes are highly activated and the bond lengths are closer to free ethylene (1.339 Å) than free acetylene (1.203 Å).^47^ The W1–C3 bond lengths in **2** (1.973(5) Å) and **3** (1.973(4) Å) as well as the C3–O3 bond lengths in **2** (1.159(7) Å) and **3** (1.149(5) Å) are not significantly different.

When comparing the structural data of the W pyridine-2-selenolates with their corresponding W pyridine-2-thiolate, besides the longer W–Se bonds, differences are small to nonsignificant. This is in line with our previous findings on Ni and Co complexes, where the exchange of an S- for Se-based ligand had little structural effects.^48^ Also, the C1–C2 bond lengths in the Se- and S-based compounds are similar, indicating identical π-backdonation. This is in contrast to the data calculated previously where in the fully selenolated model of AH a 12.74% higher π-backdonation to the acetylene was found.^10^ It is furthermore surprising since in [FeFe]-hydrogenase mimics the replacement of sulfur for selenium has been shown to enhance the electron density at the iron cores.^49^

Geometries and electronic structures of **3** and **4** were investigated by DFT/PBE0 calculations and compared to previously published results for the pyridine-2-thiolate analogs [W(CO)(C_2_H_2_)(6-MePyS)_2_] and [WO(C_2_H_2_)(6-MePyS)_2_], respectively.^23^ Optimized structures are presented in Figures S39 and S40 and selected bond lengths in Table S25 in the Supporting Information. The acetylene C≡C, W-CO, WC≡O, and W=O bond lengths are virtually identical in the pyridine-2-thiolate vs the pyridine-2-selenolate variants. The W–N and W–Se bonds are longer than the corresponding W–N and W–S bonds, as expected by the larger atomic radius of Se. Also natural population analysis (NPA) of the electronic structure of the optimized species revealed almost identical data. Only the NPA charges at tungsten are slightly different with +1.21 vs +1.11 (S vs Se) for the oxido complexes and +0.38 vs +0.28 for the CO complexes. These data are consistent with the observed similar reactivity of the two chalcogen systems.

X-ray crystallography analysis of single crystals of **6** confirms the formation of the carbyne compound with similar bond lengths and angles to that of the sulfur-based tungsten carbyne complex with 6-MePyS ligands.^14^ However, the quality of the data does not allow a detailed discussion of the structure. Nevertheless, a molecular view can be found in the Supporting Information (Figure S7).

**Figure 3:**
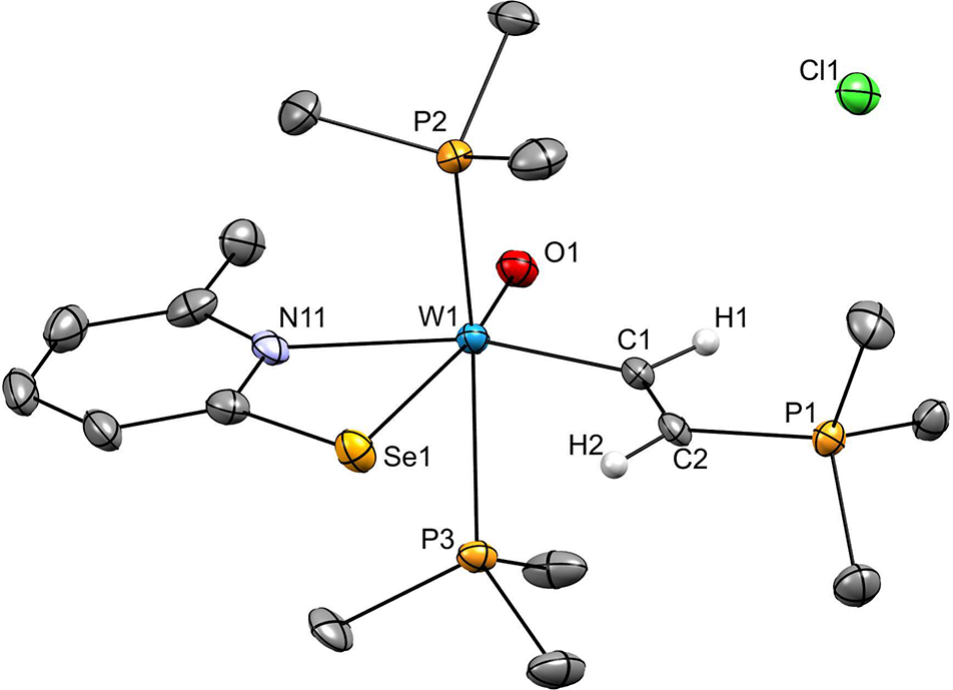
Molecular view of **7** with probability ellipsoids drawn at 50% level. Hydrogen atoms other than the ones of the ethynyl ligand are omitted for clarity.

Single crystals of **7** suitable for X-ray crystallography were obtained by recrystallization from CH_2_Cl_2_/*n*-heptane, showing high similarity with the 6-MePyS ethynyl complex^14^ (Figure 3): an octahedral coordination sphere of the W atom with an ethenyl ligand (W1–C1 2.069(6) Å, C1–C2 1.362(8) Å; C2–C1–W1 136.9(4)°) *trans* to N11 of the pyridine ring (W1–N11 2.205(5) Å), an oxo ligand (W1–O1 1.707(4) Å) *trans* to the very distant selenolato group (W1–Se1 2.8010(7) Å) and the two trimethyl phosphine ligands (W1–P2 2.4933(15)Å, W1–P3 2.4961(15)Å) *trans* to each other.

**Figure 4:**
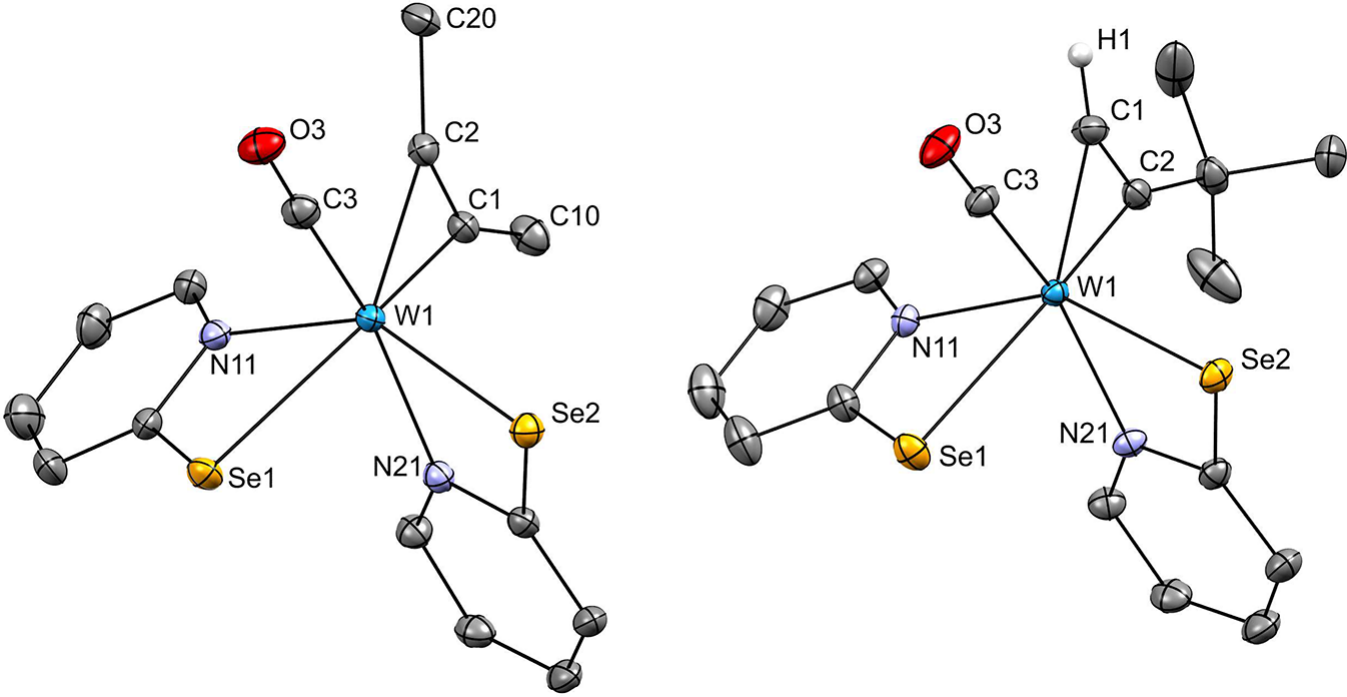
Molecular views of **8** (left) and **9** (right) with probability ellipsoids drawn at 50% level. H atoms are omitted for clarity, except for the H atom of the alkyne in **9**.

Considering the coordinated alkynes in **8** and **9** as a single coordination site, the complexes are of a distorted octahedral geometry. Like in many W(II)-complexes, the alkyne is aligned in parallel to the coordinated CO-ligand, with the *t*Bu-group in **9** pointing in the opposite direction of CO (Figure 4). The C–C triple bond is lengthened in coordinated alkynes of **8** (1.317(4) Å) and **9** (1.326(3) Å) and as such are in the range of a double bond. The double bond character is also reflected in the CCC bond angles of coordinated 2-butyne (142.0(3)° and 142.0(3)°) and 2,2-dimethyl-but-3-yne (136.27(19)°).

### IR Spectroscopy

The stretching frequency of coordinated CO is directly proportional to π-backdonation from the metal center to CO and is a good indicator of overall electron density at the metal in comparable complexes. Lower wave numbers arise from increased π-backdonation and *vice versa*. The values for tungsten carbonyl complexes **1**, **2**, **3**, and **6** and the corresponding sulfur analogs are summarized in Table 4. While the selenium-based ligand indeed provides more π-backdonation in **1**, there is no apparent difference in **2** and **3** and their sulfur-analogs. In **6** the wavenumber is even slightly higher, indicating less electron density at the metal in comparison to the analogous sulfur-based complex.

**Table 4:** Stretching Frequencies of Coordinated CO Determined by FT-IR Spectroscopy

	E = Se (cm^–1^)	E = S (cm^–1^)
[W(CO)_3_(Py**E**)_2_] (E = Se (**1**), S^13^)	2001	2013
	1905	1933
	1878	1899
[W(CO)(C_2_H_2_)(Py**E**)(CHCHPy**E**)] (E = Se (**2**), S^13^)	1890	1890
[W(CO)(C_2_H_2_)(6MePy**E**)_2_] (E = Se (**3**), S^14^)	1892	1891
[W(CO)(CCH_2_PMe_3_)(PMe_3_)_2_(6-Me-Py**E**)]Cl (E = Se (**6**), S^14^)	1853	1847

## Conclusion

Here, we report tungsten acetylene complexes containing pyridine-selenolato ancillary ligands. A standard procedure to prepare tungsten acetylene complexes of this type involves the intermediate formation of [W(CO)_3_(L)_2_] which is subsequently treated with acetylene. [W(CO)_3_(PySe)_2_] (**1**) could be isolated in contrast to the analog with 6-MePySe where only intractable mixtures were obtained. Thus, for the preparation of **[W(CO)(C_2_H_2_)(6-MePySe)_2_] (**3**)** a tungsten halide starting material with coordinated acetylene was required in which the selenolato ligand was introduced by salt metathesis. Treatment of **3** with C_2_H_2_ led to the compound with an additionally inserted molecule of acetylene [W(CO)(C_2_H_2_)(CHCH-6-MePySe)(6-MePySe)] (**5**). Since the reaction of **1** with excess acetylene led to polymerization, the analogous introduction of PySe was achieved by employing the tungsten-acetylene precursor leading exclusively to the inserted [W(CO)(C_2_H_2_)(CHCH-PySe)(PySe)] (**2**). Comparative data analysis of bond lengths in **2**, **3**, and **5** obtained from single crystal X-ray diffraction analysis with the corresponding sulfur analogs did not reveal a significant influence of Se over S in regards to the binding of acetylene. The data presented show that Se-based ligands do not increase the σ-acidity of the tungsten center while lowering its π-basicity. The carbonyl ligands in **1** can be substituted by alkynes other than acetylene giving complexes of the type [W(CO)(C_2_R_2_)(PySe)_2_] (R = Me **8**; R = H,tBu **9**). While they exhibit a single conformation in the single state as evident by single crystal X-ray diffraction, they exist as a mixture of isomers in solution, which is supported by NMR spectroscopy. Furthermore, oxidation of **3** with pyridine-N-oxide gave [WO(C_2_H_2_)(6-MePySe)_2_] (**4**) a still rare example of a tungsten(IV) acetylene complex. Nucleophilic attack of **3** and **4**, respectively, in dichloromethane led to the carbyne [W(CO)(CCH_2_PMe_3_)(PMe_3_)_2_(6-MePySe)]Cl (**6**) and the vinyl complex [WO(CHCHPMe_3_)(PMe_3_)_2_(6-MePySe)]Cl (**7**). Overall, the structures and reactivities of the obtained tungsten complexes supported by pyridine-2-selenolato ligands resemble those with the pyridine-2-thiolato ligands.

The striking structural and spectroscopic similarity of the prepared selenium-based complexes to the sulfur-based ones turned out to be of high value, as the here presented insights obtained by ^77^Se NMR spectroscopy can confidently be applied to our previous work with the pyridine-2-thiolate ligands. The selenium atom in the ligand allowed for the additional probing of the system by ^77^Se NMR spectroscopy. Surprisingly, data of tungsten(II) complexes **3**, **8**, and **9** point toward two different mesomeric forms of the pyridine-2-selenolato ligands one exhibiting pronounced selenone character, while the other one exhibiting selenolate character. Upon oxidation of tungsten, the difference in chemical shift is less pronounced. These results explain on one hand the chemoselectivity of the insertion of acetylene into the W–N bond of the ligand with a larger amide character in W(II) complexes. On the other hand, acetylene insertion is not observed in tungsten(IV) complexes because the amide character of the ligands is less pronounced, as evidenced by ^77^Se NMR spectroscopy. In contrast to unsubstituted acetylene neither 2-butyne in **8** nor 2,2,-dimethly-3-butyne in **9** were observed to insert into the W–N bond.

Despite the softer behavior of selenium, π-backdonation to coordinated acetylene was found to be equal as evidenced by the observed HC≡CH bond lengths, which is in contrast to the previous calculations.^10^ This seems to indicate that the substitution of cysteine for selenocysteine has little influence on the tungsten cofactor. In turn, the incorporation of selenium in future complexes inspired by AH allows for probing these systems by ^77^Se NMR spectroscopy. As the exchange of sulfur for selenium does not change the reactivity, according to our findings, valuable knowledge may be extracted from such systems and applied to AH.

## Experimental Section

All manipulations were performed under dinitrogen employing standard Schlenk or glovebox techniques with dry, deoxygenated solvents unless stated otherwise. All solvents were purified by a Pure Solv Solvent Purification System and were stored over activated molecular sieves (3 Å). All chemicals were purchased from commercial sources and used as-is without further purification with the exception of pyridine-*N*-oxide. Pyridine-*N*-oxide was recrystallized from diethyl ether and subsequently sublimed (55 °C, 0.1–2 mbar) prior to use.

NMR spectra were recorded using a Bruker Avance III and Bruker Avance NEO 500 MHz spectrometer. ^1^H NMR spectra were recorded at 300.13 MHz for room temperature or at 500.23 MHz for low temperature measurements and referenced to residual protons of the NMR solvents. ^13^C NMR spectra were obtained at 75.48 MHz for room temperature or at 125.80 MHz for low temperature measurements and spectra were referenced to the deuterated solvent peak. ^31^P{1H} NMR spectra were recorded at 121.49 MHz, with 85% H_3_PO_4_ as an external reference. The chemical shifts δ are given in ppm. The multiplicity of peaks is denoted as broad singlet (bs), singlet (s), doublet (d), triplet (t), quadruplet (q), multiplet (m), doublet of doublets (dd), doublet of doublet of doublets (ddd), and virtual triplet (vt). Coupling constants J are given in Hertz. IR spectra were recorded in the solid-state at a resolution of 4 cm^–1^ on a Bruker ALPHA-P Diamant ATR-FTIR. [WBr_2_(MeCN)_2_(C_2_H_2_)(CO)]/[WBr_2_(MeCN)(C_2_H_2_)_2_(CO)] was prepared according to literature.^13^

Single crystal X-ray structural analysis was performed using monochromatized Ga K_a_ radiation at 100 K on a Bruker D8 Venture Metaljet diffractometer (**2**, **3**, **4** and **7**) or monochromatized Mo K_α_ radiation at 100 K on a Bruker APEX-II CCD (**8** and **9**). Bruker APEX3 software^50^ (**2**, **3**, **4** and **7**) or APEX2 software^51^ was used to collect and reduce data and determine the space group. Absorption corrections were applied using SADABS.^52^ The structure was solved with the SHELXT structure solution program using Intrinsic Phasing (SHELXT 2018/2)^53^ and refined by full-matrix least-squares techniques against *F*^2^ (SHELXL 2019/2)^54^ using the Olex2 software.^55^ All non-hydrogen atoms were refined with anisotropic displacement parameters without any constraints. The hydrogen atoms were constrained to ideal geometries and refined with fixed isotropic displacement parameters using riding models. Crystal data, data collection parameters, structure refinement details and molecular structures of the metal complexes are provided in the Supporting Information. Selected bond lengths and angles are given in Tables S1–S24. Further refinement information, structure and bonding parameters, SHELXL. res and. hkl files are given in the deposited CIF file which is available free of charge from The Cambridge Crystallographic Data Centre (CCDC 2325188–2325193).

### Preparation of the Sodium Pyridine-2-selenolates

#### General Procedure

A 50 mL Schlenk flask was charged with selenium metal (1.50 g, 19 mmol) and degassed water (20 mL) and was placed in an ice bath. NaBH_4_ (720 mg, 20 mmol) was added and the mixture was stirred at 0 °C for 30 min before removing the ice bath during which a color change to brown was observed alongside with gas evolution. Stirring was continued at room temperature for 20 h before adding the respective 2-chloro pyridine (19 mmol) and heating the mixture to reflux for 5 h. The obtained solution was allowed to cool to room temperature before placing it in the fridge at 4 °C. After 24 h, the product had crystallized from the solution. The supernatant was removed by decantation and the crystalline product was washed with degassed water (2 × 10 mL) before drying *in vacuo*. The resulting product was taken up in oxygen-free CH_2_Cl_2_ (20 mL) and filtered through Celite to remove unreacted selenium before reducing the filtrate to dryness *in vacuo*. The purity of the selenol was then controlled by ^1^H NMR spectroscopy. Diselenide that had formed and cocrystallized does not affect the success in the next step, but must be accounted for in the stoichiometry. The crude product was dissolved in THF and NaH (0.9 equiv vs obtained selenol) was added at 0 °C. After stirring for 3 h, all volatiles were removed *in vacuo* and the resulting solids were washed with CH_2_Cl_2_ (3 × 10 mL) to remove unreacted selenol or diselenide before drying the product *in vacuo*.

**Na(PySe)** yield: 1.81 g (52%) of a pale-yellow powder

^1^H NMR (300 MHz, acetonitrile-*d*_3_) δ = 7.86 (ddd, *J* = 5.0, 2.1, 1.0, 1H, py-6-CH), 7.43 (dt, *J* = 8.0, 1.1, 1H, py-3-CH), 6.97 (ddd, *J* = 8.0, 7.2, 2.1, 1H, py-4-CH), 6.65 (ddd, *J* = 7.2, 5.1, 1.2, 1H, py-5-CH) ppm. ^13^C NMR (75 MHz, acetonitrile-*d*_3_) δ = 172.3 (Se–C), 148.8 (py-6-C), 134.2, 133.1, 118.3, 115.7 ppm.

**Na(6-MePySe)** yield: 1.54 g (41%) of a pale-yellow powder

^1^H NMR (300 MHz, acetonitrile-*d*_3_) δ = 7.24 (dt, *J* = 7.9, 0.9, 1H, 3-CH), 6.88 (t, *J* = 7.6, 1H, 4-CH), 6.52 (dt, *J* = 7.4, 0.9, 1H, 5-CH), 2.27 (s, 3H, CH_3_) ppm. ^13^C NMR (75 MHz, acetonitrile-*d*_3_) δ = 171.2 (Se–C), 157.2 (6-C), 134.7, 130.0, 115.0, 24.4 (CH_3_) ppm.

#### 2,2′-Dipyridyldiselenide (PySe)_2_

A 50 mL Schlenk flask was charged with freshly ground NaOH (600 mg, 15 mmol) and Se powder (790 mg, 10 mmol), the flask was placed under N_2_-atmosphere and deoxygenated DMF (15 mL) was added followed by hydrazine (50–60% in water, 625 μL, ca. 10 mmol). The mixture was stirred at room temperature for 3 h before adding 2-chloro-pyridine (1.14 g, 10 mmol). The reaction mixture was heated to reflux for 4 h before allowing to cool to room temperature. The resulting orange product mixture was poured on water (150 mL) and the product was extracted with dichloromethane (3 × 50 mL). The combined organic phases were dried over MgSO_4_, all volatiles were removed on the rotary evaporator and the crude product was purified by flash column chromatography to give after drying 760 mg (48%) of a yellow, crystalline solid. Spectroscopic properties match with literature.^56^

^1^H NMR (300 MHz, chloroform-*d*) δ = 8.46 (ddd, *J* = 4.8, 1.9, 0.9, 2H), 7.79 (dt, *J* = 8.1, 1.0, 2H), 7.54 (ddd, *J* = 8.0, 7.4, 1.9, 2H), 7.08 (ddd, *J* = 7.5, 4.8, 1.1, 2H) ppm.

#### Bis(6-methyl-2-pyridyl)diselenide (6-MePySe)_2_

A 50 mL Schlenk flask was charged with freshly ground NaOH (480 mg, 12 mmol) and selenium metal (630 mg, 8 mmol), the flask was evacuated and backfilled with dinitrogen. Dry, deoxygenated DMF (17 mL) was added followed by N_2_H_4_ (50–60% in water, 500 μL, ca. 8 mmol). The mixture was then stirred overnight. The next day, 2-chloro-6-methylpyridine (1.02 g, 8 mmol) was added and the mixture was refluxed for 5 h under inert conditions. The resulting dark-orange solution was stored in the freezer for 4 days. The crude product solution was poured on water (150 mL) and the product was extracted with dichloromethane (3 × 50 mL). The combined organic phases were dried over MgSO_4_ and all volatiles were removed on the rotary evaporator. The resulting yellow oil was purified by SiO_2_ flash chromatography (product R_f_ = 0.36 at cyclohexane/ethyl acetate 3 + 1). The product was dried *in vacuo* to obtain 780 mg (57%) of a yellow solid. Spectroscopic properties match with literature.^56^

^1^H NMR (300 MHz, chloroform-*d*) δ = 7.60 (d, *J* = 7.9, 1H), 7.41 (t, *J* = 7.8, 1H), 6.91 (d, *J* = 7.6, 1H), 2.53 (s, 3H) ppm.

#### [W(CO)_3_(PySe)_2_] (**1**)

In a Schlenk flask placed in an ice bath, (PySe)_2_ (490 mg, 1.56 mmol) was dissolved in methanol (10 mL) and NaBH_4_ (125 mg, 3.3 mmol) was added slowly. After stirring for 30 min, the ice bath was removed and all volatiles were removed *in vacuo*. To the dry residue, [WBr_2_(CO)_3_(MeCN)_2_] (800 mg, 1.57 mmol) was added before adding acetonitrile (20 mL). The mixture immediately turned deep red and a red-orange solid started to precipitate immediately. After stirring for 2 h, the supernatant was removed by filter cannula and the residue was washed with acetonitrile (2 × 3 mL). The product was dried thoroughly *in vacuo* to give 644 mg (71%) of a crystalline, air-stable red solid. ^1^H NMR spectroscopic data in C_6_D_6_ are in accordance with literature.^35^

^1^H NMR (300 MHz, benzene-*d*_6_) δ = 8.40 (dt, *J* = 5.5, 1.5, 2H), 6.30 (dt, *J* = 8.1, 1.2, 2H), 6.20 (td, *J* = 7.7, 1.8, 2H), 5.92 (ddd, *J* = 7.2, 5.6, 1.4, 2H) ppm. ^1^H NMR (300 MHz, chloroform-*d*) δ = 8.71 (ddd, *J* = 5.6, 1.8, 0.9, 2H), 7.35 (ddd, *J* = 8.2, 7.4, 1.7, 2H), 6.99 (dd, *J* = 8.2, 1.1, 2H), 6.94 (ddd, *J* = 7.1, 5.6, 1.3, 2H) ppm. ^77^Se NMR (57 MHz, chloroform-*d*) δ = 61.3.

IR (cm^–1^): 2001 (ν_CO_), 1905 (ν_CO_), 1878 (ν_CO_).

#### [W(CO)(C_2_H_2_)(CHCH-PySe)(PySe)] (**2**)

Na(PySe) (200 mg, 1.11 mmol) and [WBr_2_(MeCN)_2_(C_2_H_2_)(CO)]/ [WBr_2_(MeCN)(C_2_H_2_)_2_(CO)] (270 mg, 0.57 mmol W) were placed into a 50 mL Schlenk flask, which was cooled to −45 °C (MeCN/N_2_) and acetonitrile (6 mL) was added. The mixture was stirred at −45 °C for 40 min during which the color changed to deep red-purple. Subsequently, all volatiles were removed *in vacuo*. The residue was washed with diethyl ether (2 × 3 mL) and dichloromethane (10 mL) was added. The suspension was filtered through Celite and *n*-heptane (6 mL) was added to the filtrate. The mixture was again filtered through Celite and the filtrate was stored in the freezer at −30 °C. After 3 days, the product was isolated by filtration and dried *in vacuo* to give 48 mg (15% vs total W) of **1** as purple crystals.

^1^H NMR (300 MHz, dichloromethane-*d*_2_) δ = 12.90 (s, 1H), 11.94 (s, 1H), 8.26 (ddd, *J* = 5.4, 1.8, 1.0, 1H), 8.05 (ddd, *J* = 8.4, 1.6, 0.8, 1H), 7.92–7.68 (m, 2H), 7.44 (ddd, *J* = 8.1, 7.5, 1.8, 1H), 7.13 (ddd, *J* = 8.4, 7.0, 1.5, 1H), 7.06–6.91 (m, 3H), 6.40 (d, *J* = 11.0, 1H) ppm. Due to the low stability in solution and limited solubility ^13^C NMR as well as ^77^Se NMR spectra requiring long measurement times could not be obtained.

IR (cm^–1^): 1890 (ν_CO_).

Anal. Calcd for C_15_H_12_N_2_OSe_2_W: C, 31.17; H, 2.09; N, 4.85. Found: C, 30.18; H, 1.97; N, 4.41.

#### [W(CO)(C_2_H_2_)(6-Me-PySe)_2_] (**3**)

##### Method A

A 50 mL Schlenk flask was charged with (6-MePySe)_2_ (421 mg, 1.23 mmol) and NaBH_4_ (102 mg, 2.7 mmol) and cooled to 0 °C to which methanol (15 mL) was added. After stirring for 30 min the mixture was allowed to warm to room temperature and all volatiles were removed *in vacuo*. To the resulting yellow solids, the tungsten acetylene mixture [WBr_2_(MeCN)_2_(C_2_H_2_)(CO)]/[WBr_2_(MeCN)(C_2_H_2_)_2_(CO)] (600 mg, equals 1.25 mmol W) was added followed by acetonitrile (20 mL) forming a red solution. The mixture was stirred at room temperature for 30 min before removing all volatiles *in vacuo*. The resulting solids were washed with acetonitrile (2 × 5 mL) via filter cannula. Thereafter, dichloromethane (10 mL) was added and the mixture was filtered through Celite. The plug was further eluted with dichloromethane until the eluent was colorless and the combined dichloromethane phases were reduced to dryness *in vacuo* to afford 338 mg (60%) of a purple powder. Recrystallization of 120 mg of crude sample from CH_2_Cl_2_/*n*-heptane gave 82 mg (41%) of purple crystals of the pure product.

##### Method B

[WBr_2_(C_2_H_2_)(MeCN)_2_(CO)]_0.5_[WBr_2_(C_2_H_2_)_2_(MeCN)(CO)] (550 mg, 1.16 mmol W) and Na(6-MePySe) (460 mg, 2.37 mmol) were placed in a Schlenk flask, which was placed in an ice bath at 0 °C, and MeCN (6 mL) was added. The mixture was stirred at 0 °C for 30 min before removing all volatiles *in vacuo*. During the reaction the color of the mixture changed deep red/black. The resulting residue was washed with MeCN (3 × 3 mL) and the flask was transferred into the glovebox. The residue was taken up in CH_2_Cl_2_ (5 mL) and *n*-heptane (5 mL) was added. The mixture was filtered through Celite and the eluent was placed in the freezer at −30 °C. The purple, crystalline product was isolated by decantation and dried *in vacuo* to afford 148 mg (22%) of the product.

^1^H NMR (300 MHz, dichloromethane-*d*_2_) δ = 13.76 (bs, 1H, C≡CH), 12.58 (bs, 1H, C≡CH), 7.48 (t, *J* = 7.8, 1H, pyH-*p*), 7.03–6.92 (m, 2H, pyH-*m*), 6.89 (ddd, *J* = 7.9, 1.2, 0.7, 1H, pyH-*m*), 6.77 (ddd, *J* = 7.9, 1.4, 0.6, 1H, pyH-*m*), 6.68 (ddd, *J* = 7.5, 1.3, 0.6, 1H, pyH-*m*), 1.91 (s, 3H, py-CH_3_), 1.18 (s, 3H, py-CH_3_) ppm. ^13^C NMR (75 MHz, dichloromethane-*d*_2_) δ = 237.6 (CO), 206.3 (HC≡CH), 205.7 (C=Se), 172.9 (C=Se), 165.1 (py), 159.8 (py), 155.6 (py), 138.1 (py), 135.3 (py), 128.7, 128.7, 121.0, 120.1, 27.6 (CH_3_), 22.6 (CH_3_) ppm. ^77^Se NMR (57 MHz, dichloromethane-*d*_2_) δ = 365.6, 5.8 ppm. IR (cm^–1^): 1892 (s, ν_CO_).

Anal. Calcd for C_15_H_14_N_2_OSe_2_W·0.15 C_7_H_16_: C, 32.39; H, 2.78; N, 4.71. Found: C, 32.41; H, 2.60; N, 4.71.

#### Isolation of a Mixture of [W(CO)(C_2_H_2_)(6-Me-PySe)_2_] (**3**) and [W(CO)(C_2_H_2_)(CHCH-6-MePySe)(6-MePySe)] (**5**)

A solution of [W(CO)(C_2_H_2_)(6-MePySe)_2_] (65 mg, 112 μmol) in CH_2_Cl_2_ (4 mL) was purged with acetylene for 15 min. After stirring the closed flask for 24 h, it was transferred into the glovebox and *n*-heptane (4 mL) was added. The mixture was filtered through Celite and the filtrate was placed in the freezer at −30 °C. After 3 days, an orange, polycrystalline solid was obtained, which was isolated by decantation and subsequent drying *in vacuo* to afford 20 mg of product. ^1^H NMR spectroscopy revealed the crystals comprising of a mixture of **3**, **5** and at least 1 other compound with a ratio **3**/**5** = 2:1 (Figure S18).

#### [WO(C_2_H_2_)(6-Me-PySe)_2_] (**4**)

A 25 mL Schlenk flask was charged with [W(CO)(C_2_H_2_)(6-Me-PySe)_2_] (186 mg, 320 μmol) to which pyridine-*N*-oxide (53 mg, 550 μmol) and dichloromethane (6 mL) were added. The reaction mixture was stirred for 20 h at room temperature. All volatiles were removed *in vacuo* and the residue was extracted with dichloromethane (10 mL). The suspension was filtered through Celite to remove insoluble components. The obtained yellow solution was reduced to dryness *in vacuo* and the pale yellow residue was washed with acetonitrile (1.5 mL) to afford 152 mg (82%) of crude product. Recrystallization from CH_2_Cl_2_/*n*-heptane afforded the product as pale yellow crystals in 63% overall yield.

^1^H NMR (300 MHz, dichloromethane-*d*_2_) δ = 11.36 {mixture of (s, 1H, C≡CH, 92.4%) and (d, ^3^*J*_SeH_ = 6.1 Hz, 7.6%)}, 11.14 (s, 1H, C≡CH), 7.51 (t, *J* = 7.8, 1H, pyH-*p*), 7.37 (t, *J* = 7.8, 1H, pyH-*p*), 7.12 (ddd, *J* = 7.5, 5.4, 1.3, 2H, pyH-*m*), 7.02–6.94 (m, 2H, pyH-*m*), 2.61 (s, 3H, CH_3_), 2.11 (s, 3H, CH_3_) ppm. ^13^C NMR (75 MHz, dichloromethane-*d*_2_) δ = 168.6 (C=Se), 167.7 (C=Se), 160.9 (C≡C), 160.6 (C≡C), 159.5 (py), 157.2 (py), 138.7 (py), 137.0 (py), 130.5 (py), 128.5 (py), 122.1 (py), 120.6 (py), 25.9 (CH_3_), 21.5 (CH_3_) ppm. ^77^Se NMR (57 MHz, dichloromethane-*d*_2_) δ = 280.4 (d, ^3^*J*_SeH_ = 5.7 Hz), 176.8 ppm. IR (cm^–1^): 924 (ν_W=O_).

Anal. Calcd for C_14_H_14_N_2_OSe_2_W·0.10 C_7_H_16_: C, 31.96; H, 2.66; N, 4.75. found: C, 31.99; H, 2.84; N, 4.94.

#### [W(CO)(CCH_2_PMe_3_)(PMe_3_)_2_(6-Me-PySe)]Cl (**6**)

To a purple solution of [W(CO)(C_2_H_2_)(6-Me-PySe)_2_] (**3**, 45 mg, 77 μmol) in dichloromethane (10 mL) was added PMe_3_ (50 μL, 490 μmol) by syringe which led to an immediate color change to bright orange. After stirring at room temperature for 2 h, all volatiles were removed *in vacuo*. The sticky orange residue was dissolved in dichloromethane (1 mL) to which toluene (10 mL) was added. The flask was then stored at −30 °C overnight causing the product to precipitate. Removing the supernatant via filter cannula, washing the residue with diethyl ether (10 mL) and drying *in vacuo* afforded 45 mg (86%) of **6** as an orange powder.

^1^H NMR (300 MHz, dichloromethane-*d*_2_) δ = 7.05 (tt, *J* = 7.7, 1.5, 1H, pyH-*p*), 6.90 (d, *J* = 7.5, 1H, pyH-*m*), 6.71 (d, *J* = 7.9, 1H, pyH-*m*), 3.77 (dt, *J* = 19.2, 5.2, 2H, CH_2_), 2.36 (s, 3H, CH_3_), 2.15 (d, ^2^*J*_PH_ = 14.2, 9H, PCH_3_), 1.46 (t, ^2^*J*_PH_ = 3.6, 18H, WPCH_3_) ppm.

^13^C NMR (75 MHz, dichloromethane-*d*_2_) δ = 250.3 (q, *J* = 4.8, CO), 246.2 (d, *J* = 14.0, W≡C), 167.7 (pyC-*o*), 157.2 (pyC-*o*), 135.3 (t, *J*_CP_ = 2.0, pyC-*p*), 130.5 (t, *J*_CP_ = 1.7, pyC-*m*), 119.0 (pyC-*m*), 46.3 (d, ^1^*J*_CP_ = 49.6, CH_2_), 25.5 (CH_3_), 19.1 (t, ^1^*J*_CP_ = 14.0, WPCH_3_), 9.6 (d, ^1^*J*_CP_ = 54.0, PCH_3_).

^31^P NMR (121 MHz, dichloromethane-*d*_2_) δ = 20.34 (t, ^1^*J*_PC_ = 6.0, CH_2_PMe_3_), – 21.19 (d, ^2^*J*_PP_ = 5.5, WPMe_3_) ppm ^77^Se NMR (57 MHz, dichloromethane-*d*_2_) δ = −86.1 ppm. IR (cm^–1^): 1853 (ν_CO_).

Anal. Calcd for C_18_H_35_ClNOP_3_SeW·0.20 C_7_H_8_: C, 33.72; H, 5.34; N, 2.03. found: C, 33.82; H, 4.83; N, 1.95.

#### [WO(CCH_2_PMe_3_)(PMe_3_)_2_(6-Me-PySe)]Cl (**7**)

[WO(C_2_H_2_)(6-Me-PySe)_2_] (**5**, 50 mg, 88 μmol) was dissolved in dichloromethane (6 mL) in a 25 mL Schlenk flask to which PMe_3_ (35 μL, 338 μmol) was added. The color gradually changed from yellow to red to green over the course of 2 h. After stirring for 6 h, all volatiles were removed in vacuo. The crude was dissolved in dichloromethane (4 mL) and toluene (4 mL) was added. The volume of the solvent was then reduced to 5 mL by which a dark green solid precipitated. The supernatant was removed via filter cannula and the solids were washed with toluene (2 × 5 mL) and diethyl ether (5 mL). After drying in vacuo, 56 mg (96%) of crude **7** were obtained as a green powder. The product could not be purified as all recrystallization attempts led to more decomposition. ^1^H NMR spectroscopy reveals approximately 93% purity.

^1^H NMR (300 MHz, dichloromethane-*d*_2_) δ = 11.49 (dd, *J* = 37.4, 18.4, 1H, CH), 7.05 (d, *J* = 4.5, 2H, pyH-*p* and pyH-*m*), 6.83 (t, *J* = 4.6, 1H, pyH-*m*), 4.26 (dd, *J* = 36.7, 19.7, 1H, CH), 2.54 (s, 3H, py-CH_3_), 1.94 (d, ^2^*J*_PH_ = 13.4, 9H, C–P(CH_3_)_3_), 1.43 (t, ^2^*J*_PH_ = 4.2, 18H, W–P(CH_3_)_3_) ppm.

^1^H NMR (300 MHz, dichloromethane-*d*_2_) δ = 11.48 (dd, *J* = 37.4, 18.4, 1H, CH), 7.13–6.98 (m, 2H, pyH-*p*), 6.84 (q, *J* = 4.1, 1H, pyH-*m*), 4.26 (dd, *J* = 37.8, 17.7, 1H, CH), 2.54 (s, 3H, py-CH_3_), 1.94 (d, ^2^*J*_PH_ = 13.5, 9H, C–P(CH_3_)_3_), 1.43 (t, ^2^*J*_PH_ = 4.2, 18H, W–P(CH_3_)_3_) ppm. ^13^C NMR (75 MHz, dichloromethane-*d*_2_) δ = 221.7 (WCH), 159.3 (pyC-*o*), 155.8 (pyC-*o*), 134.9 (pyC-*p*), 129.5 (pyC-*m*), 119.9 (pyC-*m*), 25.4 (CH_3_), 14.7 (t, ^1^*J*_CP_ = 15.6, WPCH_3_), 13.2 (d, ^1^*J*_CP_ = 58.0, PCH_3_) ppm. ^31^P NMR (121 MHz, dichloromethane-*d*_2_) δ = 5.24 (CHPMe_3_), – 25.17 (t, *J* = 173.6, WPMe_3_).

IR (cm^–1^): 2967, 2898, 1442, 1430, 1408, 942 (ν_W=O_).

Anal. Calcd for C_17_H_35_ClNOP_3_SeW·0.15 C_7_H_8_: C, 32.14; H, 5.41; N, 2.08. found: C, 32.51; H, 4.83; N, 1.75.

#### [W(CO)(MeCCMe)(PySe)_2_] (**8**)

A solution of [W(CO)_3_(PySe)_2_] (**1**, 84 mg, 0.14 mmol) and 2-butyne (100 μL, 63 mg, 1.2 mmol) in toluene (10 mL) was stirred at 70 °C for 6 h. The color of the solution changed from red to green. Subsequently, all volatiles were removed *in vacuo* and the resulting red residue was recrystallized from dichloromethane/*n*-heptane at −30 °C to give 49 mg (60%) of red crystals.

^1^H NMR (300 MHz, chloroform-*d*) δ = 9.11 (isomer A, dt, *J* = 5.3, 1.6, 1H, *o*-N-py-H), 8.85 (isomer A, dt, *J* = 5.6, 1.4, 1H, *o*-N-py-H), 7.73 (isomer B, dt, *J* = 5.3, 1.4, 0H, *o*-N-py-H), 7.65–7.46 (nondeterminable, m, 2H), 7.23 (isomer A, td, *J* = 7.7, 1.8, 1H), 7.14 (nondeterminable, m, 2H), 7.06 (isomer A, dd, *J* = 8.0, 1.1, 1H), 7.03–6.85 (nondeterminable, m, 3H), 6.73 (isomer B, dt, *J* = 5.7, 1.4, 0H), 6.66 (isomer B, ddd, *J* = 7.3, 5.7, 1.8, 1H), 3.14 (nondeterminable, s, 9H) ppm. ^13^C NMR (126 MHz, chloroform-*d*, – 30 °C) δ = 241.1 (CO), 239.6 (CO), 213.5 (butyne-C, isomer A), 212.3(butyne-C, isomer A), 210.2(butyne-C, isomer B), 208.8 (butyne-C, isomer B), 172.1 (C=Se, isomer B), 171.2 (C=Se, isomer A), 163.8 (C=Se, isomer B), 163.4 (C=Se, isomer A), 149.0 (py-6-C, isomer A), 148.4 (py-6-C, isomer A), 145.9 (py-6-C, isomer B), 145.8 (py-6-C, isomer B), 138.4 (py-C, isomer A), 137.8 (py-C, isomer B), 136.3 (py-C, isomer A), 134.4 (py-C, isomer B), 131.8 (py-C, isomer B), 131.2 (py-C, isomer B), 130.5 (py-C, isomer A), 130.2 (py-C, isomer A), 120.9 (py-C, isomer A), 120.9 (py-C, isomer B), 119.1 (py-C, isomer A), 118.9 (py-C, isomer B), 22.7 (butyne-CH_3_, isomer B), 22.5 (butyne-CH_3_, isomer A), 18.3 (butyne-CH_3_, isomer B), 17.7 (butyne-CH_3_, isomer A) ppm. ^77^Se NMR (57 MHz, chloroform-*d*) δ = 334.8 (isomer B), 295.7 (isomer A), 45.4 (isomer B), – 80.4 (isomer A) ppm. IR (cm^–1^): 1898 (ν_CO_)

Anal. Calcd for C_15_H_14_N_2_OSe_2_W·0.10 C_7_H_16_: C, 31.96; H, 2.66; N, 4.75. Found: C, 31.99; H, 2.84; N, 4.94.

#### [W(CO)(HCC*t*-Bu)(PySe)_2_] (**9**)

A 25 mL Schlenk flask was charged with [W(CO)_3_(PySe)_2_] (100 mg, 172 μmol), 2,2-dimethyl-3-butyne (115 mg, 1.4 mmol) and toluene (5 mL). The mixture was heated to 80 °C for 90 min. Subsequently, all volatiles were removed *in vacuo* and the residue was washed with dry pentane (5 mL). Recrystallization of the crude red powder from CH_2_Cl_2_/*n*-heptane at −30 °C gave 86 mg (84%) of red crystals.

^1^H NMR (500 MHz, chloroform-*d*) δ = 13.40 (s, C≡C–H, isomer B), 13.32 (s, C≡C–H, isomer A), 12.10 (s, C≡C–H, isomer C), 11.90 (s, C≡C–H, isomer D), 9.24 (ddd, *J* = 5.4, 1.8, 0.9, 1H, pyH-*o*, isomer A), 9.12 (d, *J* = 5.3, pyH-*o*, isomer C or D), 8.87 (d, *J* = 5.7, pyH-*o*, isomer C or D), 8.70 (ddd, *J* = 5.6, 1.8, 0.9, 1H pyH-*o*, isomer A), 7.92–7.82 (m, isomer C or D), 7.77 (dt, *J* = 5.4, 1.3, pyH-*o*, isomer B), 7.61 (m, pyH, multiple isomers), 7.34–7.21 (m, pyH, multiple isomers), 7.21–7.09 (m, pyH, multiple isomers), 7.08–7.03 (m, pyH, multiple isomers), 7.04–6.79 (m, pyH, multiple isomers), 6.69 (m, pyH, multiple isomers), 1.52 (s, C≡C-*t*Bu, isomer D), 1.51 (s, C≡C-*t*Bu, isomer C) 1.23 (s, C≡C-*t*Bu, isomer B), 1.21 (s, 9H, C≡C-*t*Bu, isomer A) ppm. ^13^C NMR (126 MHz, chloroform-*d*) δ = 243.3 (CO), 230.0 (CO), 204.4 (C≡C), 204.4 (C≡C), 173.5, 172.1, 163.9, 148.8 (pyC), 148.3 (pyC), 147.1 (pyC), 146.7 (pyC), 138.8 (pyC), 137.7 (pyC), 136.3 (pyC), 134.1 (pyC), 131.8 (pyC), 131.2 (pyC), 130.4 (pyC), 130.2 (pyC), 120.3 (pyC), 120.1 (pyC), 119.1 (pyC), 118.5 (pyC), 42.1 (*t*Bu), 41.9 (C(**C**H_3_)_3_), 31.5 (C(**C**H_3_)_3_), 30.9 (*t*Bu), 30.8 (**C**Me_3_), 30.7 (**C**Me_3_) ppm. ^77^Se NMR (57 MHz, chloroform-*d*) δ = 330.0 (isomer B), 303.4 (isomer A), 52.4 (isomer B), – 86.3 (isomer A). IR (cm^–1^): 1905 (ν_CO_), 752 (ν_tBu_).

Anal. Calcd for C_17_H_18_N_2_OSe_2_W: C, 33.58; H, 2.98; N, 4.61. Found: C, 33.68; H, 2.91; N, 4.58.

## Supporting Information Available

NMR spectra and crystallographic data (PDF)
