# Supplementary material for: The Effect of Selenium-Based Ligands on Tungsten Acetylene Complexes
Source: Inorg Chem. 2024 Jun 20;63(26):12255–67. doi: 10.1021/acs.inorgchem.4c01636 (PMC11220754; doi:10.1021/acs.inorgchem.4c01636)
Supplement: Supplementary file 1 — ic4c01636_si_001.pdf [file ic4c01636_si_001.pdf]

Supporting Information

The Effect of Selenium-Based Ligands on Tungsten Acetylene Complexes

Lorenz Steiner,[a] Antoine Dupé,[a] Karl Kirchner,[b] Nadia C. Mösch-Zanetti, [a]*

[a]Institute of Chemistry, Inorganic Chemistry, University of Graz, 8010 Graz, Austria

[b]Institute of Applied Synthetic Chemistry, Vienna University of Technology, 1060 Vienna, Austria

Table of Contents

General Considerations 2
X-Ray Data 4
NMR Data 23
Computational Details 54
References 63

General Considerations

All manipulations were performed under dinitrogen employing standard Schlenk or glovebox techniques with dry, deoxygenated solvents, unless stated otherwise. All solvents were purified by a Pure Solv Solvent Purification System and were stored over activated molecular sieves (3 Å). All chemicals were purchased from commercial sources and used as-is without further purification with the exception of pyridine-*N*-oxide. Pyridine-*N*-oxide was recrystallized from diethyl ether and subsequently sublimed (55 °C, 0.1-2 mbar) prior to use.

NMR spectra were recorded using a Bruker Avance III and Bruker Avance NEO 500 MHz spectrometer. ^{1}H NMR spectra were recorded at 300.13 MHz for room temperature or at 500.23 MHz for low temperature measurements and referenced to residual protons of the NMR solvents. ^{13}C NMR spectra were obtained at 75.48 MHz for room temperature or at 125.80 MHz for low temperature measurements and spectra were referenced to the deuterated solvent peak. ^{31}P{1H} spectra were recorded at 121.49 MHz, with 85% H_3PO_4 as an external reference. The chemical shifts δ are given in ppm. The multiplicity of peaks is denoted as broad singlet (bs), singlet (s), doublet (d), triplet (t), quadruplet (q), multiplet (m), doublet of doublets (dd), doublet of doublet of doublets (ddd), and virtual triplet (vt). Coupling constants J are given in Hertz. IR spectra were recorded in the solid-state at a resolution of 4 cm^{-1} on a Bruker ALPHA-P Diamant ATR-FTIR. $[WBr_2(MeCN)_2(C_2H_2)(CO)]/[WBr_2(MeCN)(C_2H_2)_2(CO)]$ was prepared according to literature.[1] X-Ray structural analysis was performed using monochromatized Ga K_a radiation at 100K on a Bruker D8 Venture Metaljet diffractometer (**2**, **3**, **4** and **7**) or monochromatized Mo K_α radiation at 100K on a Bruker APEX-II CCD (**8** and **9**). Bruker APEX3 software[2] (**2**, **3**, **4** and **7**) or APEX2 software[3] (**8** and **9**) was used to collect and reduce data and determine the space group. Absorption

corrections were applied using SADABS.[4] The structures were solved with the SHELXT structure solution program using Intrinsic Phasing (SHELXT 2018/2)[5] and refined by full-matrix least-squares techniques against F^2 (SHELXL 2019/2)[6] using the Olex2 software,[7] except for compound **4** for which the structure was solved with the SHELXS structure solution program using direct methods (SHELXS 2014/6).[8] All non-hydrogen atoms were refined with anisotropic displacement parameters without any constraints. Hydrogen atom positions were calculated geometrically and refined using a riding model. CCDC 2325188 – 2325193 contain the supplementary crystallographic data for this paper. This data can be obtained free of charge via http://www.ccdc.cam.ac.uk/ or from Cambridge Crystallographic Data Centre, 12 Union Road, Cambridge, CB2 1EZ, UK.

X-Ray Data

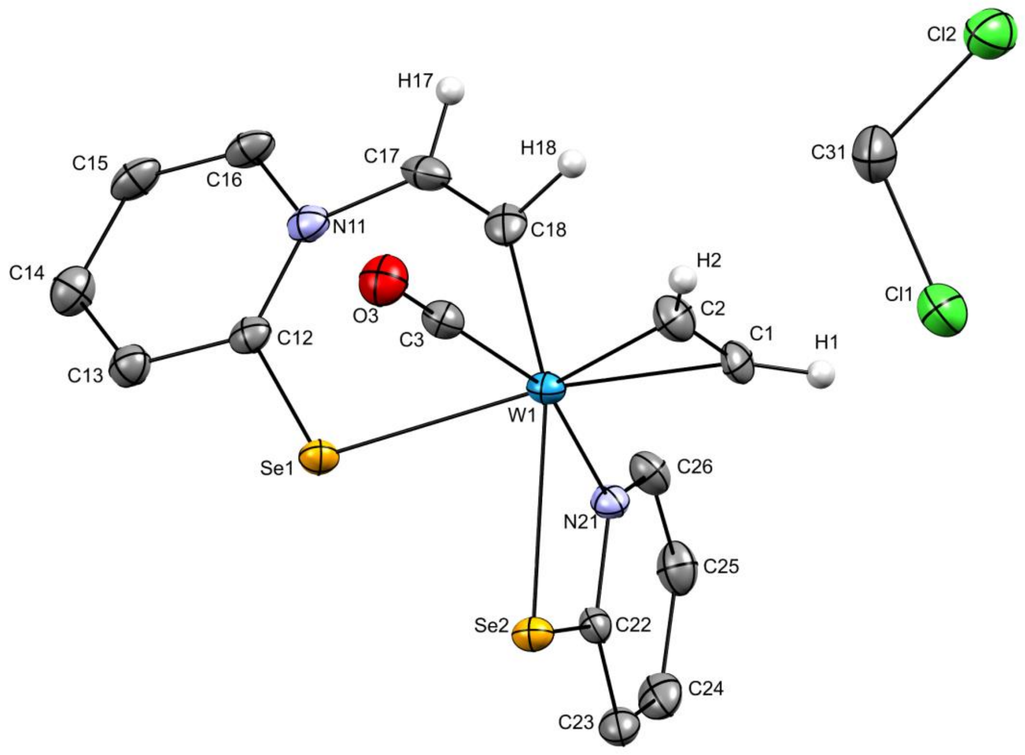

Figure S1. Molecular structure of the metal complex of [W(CO)(C_2H_2)(CHCH-PySe)(PySe)] (**2**) showing the atomic numbering scheme. The probability ellipsoids are drawn at the 50% probability level. Except for those of the ethyne and ethene groups, the H atoms were omitted for clarity.

Table S1. Crystal data and structure refinement for [W(CO)(C_2H_2)(CHCH-PySe)(PySe)] (**2**).

CCDC n°	2325188
Empirical formula	$C_{15}H_{12}N_2OSe_2W \cdot CH_2Cl_2$
Formula weight	662.96
Temperature /K	100.00
Crystal system	triclinic
Space group	P-1
a /Å	7.3648(3)
b /Å	10.3927(4)
c /Å	12.6314(5)
α /°	77.3220(10)
β /°	88.0970(10)
γ /°	81.2200(10)
Volume /Å^3	932.18(6)
Z	2
ρ_{calc} g/cm^3	2.362
μ /mm^{-1}	12.630
F(000)	616.0
Crystal size /mm^3	0.15 × 0.1 × 0.05
Radiation	GaKα (λ = 1.34139)
2Θ range for data collection /°	6.24 to 127.038
Index ranges	$-9 \le h \le 9$, $-13 \le k \le 13$, $-16 \le l \le 16$
Reflections collected	21128
Independent reflections	4596 [R_{int} = 0.0452, R_{sigma} = 0.0353]
Data/restraints/parameters	4596/0/217
Goodness-of-fit on F^2	1.062
Final R indexes [I>=2σ (I)]	R_1 = 0.0391, wR_2 = 0.1005
Final R indexes [all data]	R_1 = 0.0416, wR_2 = 0.1026
Largest diff. peak/hole / eÅ^{-3}	4.37/-2.71

Table S2. Selected bond lengths [Å] for [W(CO)(C_2H_2)(CHCH-PySe)(PySe)] (**2**).

W1	C1	2.040(5)	W1	N21	2.240(4)
W1	C2	2.062(6)	C1	C2	1.319(8)
W1	C3	1.973(5)	C3	O3	1.159(7)
W1	C18	2.113(6)	C17	C18	1.322(8)
W1	Se1	2.6683(6)	C17	N11	1.436(7)
W1	Se2	2.5919(6)			

Table S3. Selected bond angles [°] for [W(CO)(C_2H_2)(CHCH-PySe)(PySe)] (**2**).

C1	W1	C2	37.5(2)	C3	W1	Se2	102.79(15)
C1	W1	C18	100.6(2)	C18	W1	Se1	80.34(15)
C1	W1	Se1	165.04(16)	C18	W1	Se2	149.60(15)
C1	W1	Se2	99.12(16)	Se1	W1	Se2	74.751(18)
C1	W1	N21	85.09(19)	C3	W1	N21	162.62(19)
C2	W1	N21	122.1(2)	C18	W1	N21	92.13(18)
C2	W1	C18	103.1(2)	N21	W1	Se1	79.95(11)
C2	W1	Se1	157.09(17)	N21	W1	Se2	66.77(11)
C2	W1	Se2	106.65(17)	C2	C1	W1	72.2(3)
C3	W1	C1	110.9(2)	C1	C2	W1	70.3(3)
C3	W1	C2	73.4(2)	O3	C3	W1	178.9(5)
C3	W1	C18	91.5(2)	C17	C18	W1	145.5(4)
C3	W1	Se1	83.90(15)	C18	C17	N11	129.1(5)

Table S4. Selected Torsion angles [°] for [W(CO)(C_2H_2)(CHCH-PySe)(PySe)] (**2**).

W1	C18	C17	N11	3.1(11)	C17	C18	W1	C3	-89.0(7)
C1	C2	W1	C3	-178.0(4)	C12	N11	C17	C18	5.9(9)
C2	C1	W1	C3	2.0(4)	C16	N11	C17	C18	-176.5(6)

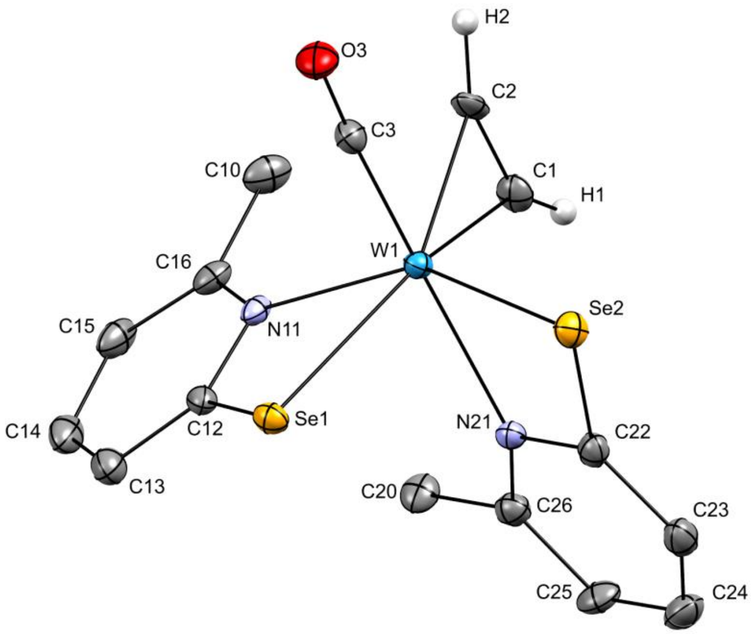

Figure S2. Molecular structure of $[W(CO)(C_2H_2)(6\text{-MePySe})_2]$ (**3**) showing the atomic numbering scheme. The ellipsoids are drawn at the 50% probability level. Except for those of the ethyne ligand, the H atoms were omitted for clarity.

Table S5. Crystal data and structure refinement for [W(CO)(C_2H_2)(6-MePySe)$_2$] (**3**).

CCDC n°	2325189
Empirical formula	$C_{15}H_{14}N_2OSe_2W$
Formula weight	580.05
Temperature/K	109.00
Crystal system	orthorhombic
Space group	$P2_12_12_1$
a/Å	7.2207(8)
b/Å	8.1050(9)
c/Å	27.043(3)
α/°	90
β/°	90
γ/°	90
Volume/Å^3	1582.6(3)
Z	4
ρ_{calc}g/cm^3	2.434
μ/mm^{-1}	12.760
F(000)	1072.0
Crystal size/mm^3	0.26 × 0.22 × 0.05
Radiation	GaKα (λ = 1.34139)
2Θ range for data collection/°	9.912 to 123.916
Index ranges	$-9 \le h \le 9$, $-10 \le k \le 10$, $-35 \le l \le 35$
Reflections collected	37481
Independent reflections	3793 [R_{int} = 0.0442, R_{sigma} = 0.0200]
Data/restraints/parameters	3793/0/193
Goodness-of-fit on F^2	1.064
Final R indexes [I>=2σ (I)]	R_1 = 0.0162, wR_2 = 0.0385
Final R indexes [all data]	R_1 = 0.0168, wR_2 = 0.0388
Largest diff. peak/hole / e Å^{-3}	0.60/-0.90

Table S6. Selected bond lengths [Å] for [W(CO)(C_2H_2)(6-MePySe)$_2$] (**3**).

W1	C1	2.022(4)	W1	Se2	2.5184(5)
W1	C2	2.045(4)	W1	N11	2.215(3)
W1	C3	1.973(4)	W1	N21	2.268(3)
C1	C2	1.305(6)	Se1	C12	1.889(4)
C3	O3	1.149(5)	Se2	C22	1.907(4)
W1	Se1	2.6929(5)			

Table S7. Selected bond angles [°] for [W(CO)(C_2H_2)(6-MePySe)$_2$] (**3**).

C1	W1	C2	37.44(18)	C3	W1	C1	109.55(17)
C2	C1	W1	72.2(3)	C3	W1	C2	72.12(17)
C1	C2	W1	70.3(2)	C3	W1	Se1	88.37(12)
C1	W1	Se1	158.20(13)	C3	W1	Se2	95.40(13)
C1	W1	Se2	104.22(13)	C3	W1	N11	98.35(15)
C2	W1	Se1	156.41(12)	C3	W1	N21	161.54(15)
C2	W1	Se2	108.70(12)	Se1	W1	Se2	85.697(17)
C1	W1	N11	98.69(16)	N11	W1	Se2	147.49(9)
C1	W1	N21	83.36(15)	N21	W1	Se1	82.51(9)
C2	W1	N11	103.54(15)	N11	W1	N21	92.43(12)
C2	W1	N21	119.86(15)				

Table S8. Torsion angles [°] for [W(CO)(C_2H_2)(6-MePySe)$_2$] (**3**).

C2	C1	W1	C3	-1.2(3)
C1	C2	W1	C3	178.83(17)

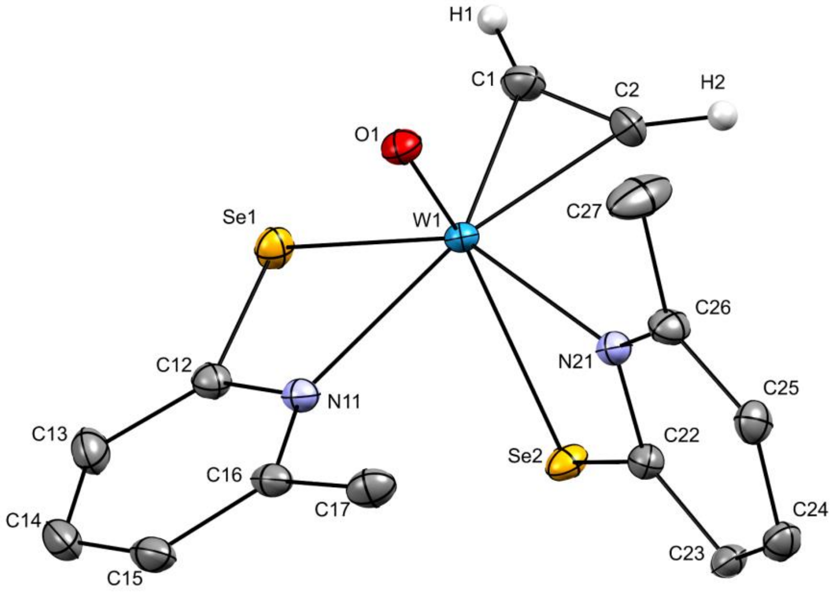

Figure S3. Molecular structure of the metal complex of [$WO(C_2H_2)(6\text{-}MePySe)_2$] (**4**) showing the atomic numbering scheme. The probability ellipsoids are drawn at the 50% probability level. Except for those of the ethyne ligand, the H atoms were omitted for clarity.

Table S9. Crystal data and structure refinement for [WO(C_2H_2)(6-MePySe)$_2$] (**4**).

CCDC n°	2325190
Empirical formula	$C_{14}H_{14}N_2OSe_2W$
Formula weight	568.04
Temperature/K	100.00
Crystal system	orthorhombic
Space group	Pbca
a/Å	14.7718(7)
b/Å	13.5698(7)
c/Å	15.3856(7)
α/°	90
β/°	90
γ/°	90
Volume/Å^3	3084.0(3)
Z	8
ρ_{calc}g/cm^3	2.447
μ/mm^{-1}	13.081
F(000)	2096.0
Crystal size/mm^3	0.38 × 0.35 × 0.13
Radiation	GaKα (λ = 1.34139)
2Θ range for data collection/°	9.18 to 141.676
Index ranges	$-20 \leq h \leq 20$, $-19 \leq k \leq 18$, $-21 \leq l \leq 21$
Reflections collected	62297
Independent reflections	4492 [R_{int} = 0.0483, R_{sigma} = 0.0202]
Data/restraints/parameters	4492/0/183
Goodness-of-fit on F^2	1.298
Final R indexes [I>=2σ (I)]	R_1 = 0.0247, wR_2 = 0.0627
Final R indexes [all data]	R_1 = 0.0247, wR_2 = 0.0627
Largest diff. peak/hole / e Å^{-3}	0.75/-2.11

Table S10. Selected bond lengths [Å] for [WO(C_2H_2)(6-MePySe)$_2$] (**4**).

W1	C1	2.075(3)	W1	Se2	2.7429(3)
W1	C2	2.088(3)	W1	N11	2.293(2)
W1	O1	1.720(2)	W1	N21	2.216(2)
C1	C2	1.280(4)	Se1	C12	1.893(3)
W1	Se1	2.5285(3)	Se2	C22	1.879(3)

Table S11. Selected bond angles [°] for [WO(C_2H_2)(6-MePySe)$_2$] (**4**).

C1	W1	C2	35.8(5)	C1	W1	Se2	102.67(8)
C1	W1	N11	147.33(11)	C2	W1	Se2	89.38(8)
C1	W1	N21	119.45(10)	O1	W1	Se2	153.95(7)
C2	W1	N11	168.08(10)	Se1	W1	Se2	87.012(11)
C2	W1	N21	83.69(10)	C2	C1	H1	143.7(13)
C1	W1	Se1	80.46(9)	C1	C2	H2	144.2(13)
C2	W1	Se1	112.95(9)	N11	W1	N21	91.00(9)

Table S12. Selected torsion angles [°] for [WO(C_2H_2)(6-MePySe)$_2$] (**4**).

C1	C2	W1	O1	-89.5(8)	C1	C2	W1	Se1	26.5(9)
C2	C1	W1	O1	98.8(8)	C2	C1	W1	Se1	-155.4(8)
C1	C2	W1	N21	177.5(8)					
C2	C1	W1	N21	-2.9(10)					

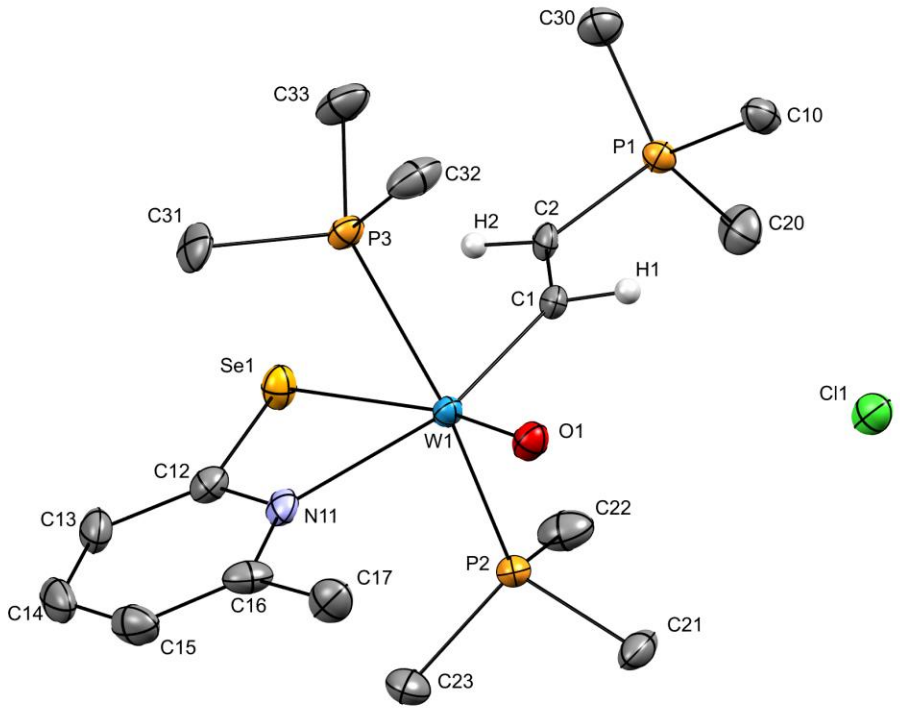

Figure S4. Molecular structure of the metal complex of [$WO(CHCHPMe_3)(PMe_3)_2$(6-MePySe)]Cl (**7**) showing the atomic numbering scheme. The probability ellipsoids are drawn at the 50% probability level. Except for those of the ethenyl ligand, the H atoms were omitted for clarity.

Table S13. Crystal data and structure refinement for [WO(CHCHPMe$_3$)(PMe$_3$)$_2$(6-MePySe)]Cl (**7**).

CCDC n°	2325191
Empirical formula	$C_{17}H_{35}ClNOP_3SeW$
Formula weight	660.63
Temperature/K	100.00
Crystal system	orthorhombic
Space group	Pbca
a/Å	23.1664(12)
b/Å	11.7509(6)
c/Å	18.9618(10)
α/°	90
β/°	90
γ/°	90
Volume/Å^3	5161.9(5)
Z	8
ρ_{calc}g/cm^3	1.700
μ/mm^{-1}	8.582
F(000)	2576.0
Crystal size/mm^3	0.17 × 0.13 × 0.1
Radiation	GaKα (λ = 1.34139)
2Θ range for data collection/°	6.638 to 105.982
Index ranges	$-27 \leq h \leq 27$, $-13 \leq k \leq 13$, $-21 \leq l \leq 22$
Reflections collected	69239
Independent reflections	4464 [R_{int} = 0.0623, R_{sigma} = 0.0264]
Data/restraints/parameters	4464/0/236
Goodness-of-fit on F^2	1.190
Final R indexes [I>=2σ (I)]	R_1 = 0.0424, wR_2 = 0.0857
Final R indexes [all data]	R_1 = 0.0438, wR_2 = 0.0862
Largest diff. peak/hole / e Å^{-3}	1.82/-1.05

Table S14. Selected bond lengths [Å] for [WO(CHCHPMe$_3$)(PMe$_3$)$_2$(6-MePySe)]Cl (**7**).

W1	C1	2.069(6)	C2	P1	1.748(6)
W1	O1	1.707(4)	P1	C10	1.774(6)
W1	Se1	2.8010(7)	P1	C20	1.783(7)
C1	C2	1.362(8)	P1	C30	1.793(6)
W1	N11	2.205(5)	P2	C21	1.815(6)
W1	P2	2.4933(15)	P2	C22	1.809(7)
W1	P3	2.4961(15)	P2	C23	1.826(6)

Table S15. Selected bond angles [°] for [WO(CHCHPMe$_3$)(PMe$_3$)$_2$(6-MePySe)]Cl (**7**).

O1	W1	Se1	169.28(14)	C2	C1	H1	111.6(5)
C1	W1	N11	151.6(2)	C1	C2	P1	123.2(4)
P2	W1	P3	160.86(5)	C12	Se1	W1	74.88(19)
C2	C1	W1	136.9(4)				

Table S16. Selected torsion angles [°] for [WO(CHCHPMe$_3$)(PMe$_3$)$_2$(6-MePySe)]Cl (**7**).

W1	C1	C2	P1	-179.0(3)	
C1	C2	P1	C10	-21.4(6)	
C2	C1	W1	Se1	1.9(6)	
C2	C1	W1	O1	-177.2(6)	

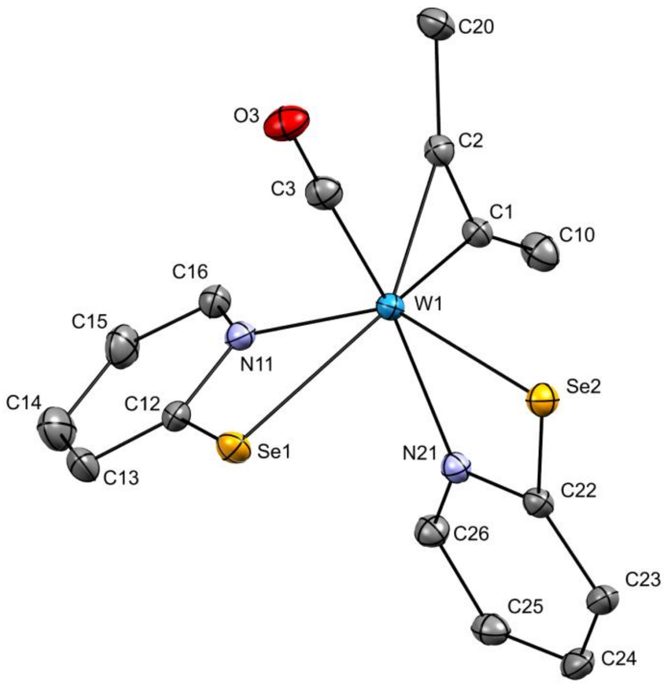

Figure S5. Molecular structure of the metal complex of [W(CO)(MeCCMe)(PySe)$_2$] (**8**) showing the atomic numbering scheme. The probability ellipsoids are drawn at the 50% probability level. the H atoms were omitted for clarity.

Table S17. Crystal data and structure refinement for [W(CO)(MeCCMe)(PySe)$_2$] (**8**).

CCDC n°	2325192
Empirical formula	$C_{15}H_{14}N_2OSe_2W$
Formula weight	580.05
Temperature /K	100.01
Crystal system	monoclinic
Space group	$P2_1/c$
a /Å	9.6456(18)
b /Å	12.869(2)
c /Å	13.741(3)
α /°	90
β /°	103.035(5)
γ /°	90
Volume /Å^3	1661.6(5)
Z	4
ρ_{calc} g/cm^3	2.319
μ /mm^{-1}	11.332
F(000)	1072.0
Crystal size /mm^3	0.2 × 0.15 × 0.05
Radiation	MoKα (λ = 0.71073)
2Θ range for data collection /°	4.334 to 66.54
Index ranges	$-14 \le h \le 14, -19 \le k \le 19, -21 \le l \le 21$
Reflections collected	75458
Independent reflections	6373 [R_{int} = 0.1008, R_{sigma} = 0.0405]
Data/restraints/parameters	6373/0/192
Goodness-of-fit on F^2	1.050
Final R indexes [I>=2σ (I)]	R_1 = 0.0277, wR_2 = 0.0651
Final R indexes [all data]	R_1 = 0.0377, wR_2 = 0.0702
Largest diff. peak/hole / eÅ^{-3}	2.77/-1.92

Table S18. Selected bond lengths [Å] for [W(CO)(MeCCMe)(PySe)$_2$] (**8**).

W1	C1	2.015(3)	W1	N21	2.225(3)
W1	C2	2.053(3)	C1	C2	1.317(4)
W1	C3	1.971(3)	C3	O3	1.158(4)
W1	Se1	2.7156(5)	Se1	C12	1.885(3)
W1	Se2	2.5539(5)	Se2	C22	1.920(3)
W1	N11	2.167(3)			

Table S19. Selected bond angles [°] for [W(CO)(MeCCMe)(PySe)$_2$] (**8**).

C1	W1	C2	37.76(11)	N11	W1	N21	85.81(9)
C1	W1	Se1	156.25(8)	Se1	W1	Se2	88.801(15)
C1	W1	Se2	104.65(9)	C2	C1	C10	142.0(3)
C3	W1	N21	163.54(11)	C1	C2	C20	142.0(3)
N11	W1	Se2	145.25(7)	O3	C3	W1	177.8(3)

Table S20. Selected torsion angles [°] for [W(CO)(MeCCMe)(PySe)$_2$] (**8**).

C1	C2	W1	C3	-178.3(2)
C2	C1	W1	C3	1.8(2)

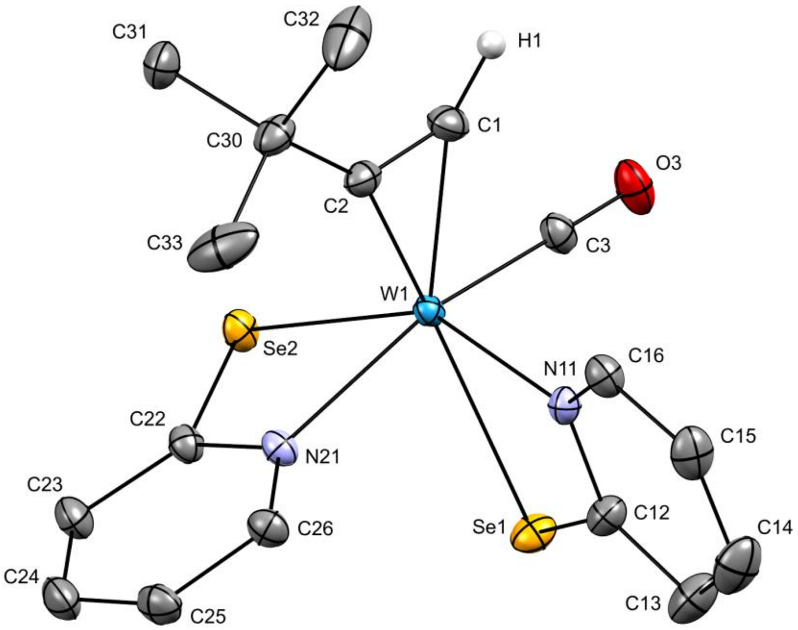

Figure S6. Molecular structure of the metal complex of [W(CO)(HCC*t*-Bu)(PySe)$_2$] (**9**) showing the atomic numbering scheme. The probability ellipsoids are drawn at the 50% probability level. Except the H atoms were omitted for clarity.

Table S21. Crystal data and structure refinement for [W(CO)(HCC*t*-Bu)$(PySe)_2$] (**9**).

CCDC n°	2325193
Empirical formula	$C_{17}H_{18}N_2OSe_2W$
Formula weight	608.10
Temperature/K	99.99
Crystal system	monoclinic
Space group	$P2_1/n$
a/Å	8.5235(7)
b/Å	14.1999(12)
c/Å	15.3246(13)
α/°	90
β/°	93.576(2)
γ/°	90
Volume/Å^3	1851.2(3)
Z	4
ρ_{calc}g/cm^3	2.182
μ/mm^{-1}	10.177
F(000)	1136.0
Crystal size/mm^3	0.23 × 0.15 × 0.1
Radiation	MoKα (λ = 0.71073)
2Θ range for data collection/°	5.326 to 69.932
Index ranges	$-13 \leq h \leq 13$, $-22 \leq k \leq 22$, $-24 \leq l \leq 24$
Reflections collected	107182
Independent reflections	8137 [R_{int} = 0.0584, R_{sigma} = 0.0236]
Data/restraints/parameters	8137/0/211
Goodness-of-fit on F^2	1.065
Final R indexes [I>=2σ (I)]	R_1 = 0.0213, wR_2 = 0.0543
Final R indexes [all data]	R_1 = 0.0252, wR_2 = 0.0558
Largest diff. peak/hole / e Å^{-3}	1.87/-1.16

Table S22. Selected bond lengths [Å] for [W(CO)(HCC*t*-Bu)(PySe)$_2$] (**9**).

W1	C1	2.022(2)	W1	N11	2.1628(16)
W1	C2	2.034(2)	W1	N21	2.2192(16)
W1	C3	1.973(2)	C1	C2	1.326(3)
C3	O3	1.157(3)	C2	C30	1.511(3)
W1	Se1	2.7288(3)	Se1	C12	1.883(2)
W1	Se2	2.5426(3)	Se2	C22	1.9097(19)

Table S23. Selected bond angles [°] for [W(CO)(HCC*t*-Bu)(PySe)$_2$] (**9**).

C1	W1	Se1	150.08(6)	N11	W1	Se2	148.29(4)
C2	W1	Se1	159.87(6)	N11	W1	N21	87.97(6)
C1	W1	C2	38.15(9)	C1	C2	C30	136.27(19)
Se2	W1	Se1	90.458(8)	C30	C2	W1	153.06(15)
C3	W1	N21	158.82(7)	O3	C3	W1	179.5(2)

Table S24. Selected torsion angles [°] for [W(CO)(HCC*t*-Bu)(PySe)$_2$] (**9**).

W1	C1	C2	C30	-175.6(2)
C1	C2	W1	C3	0.94(13)
C2	C1	W1	C3	-179.09(13)

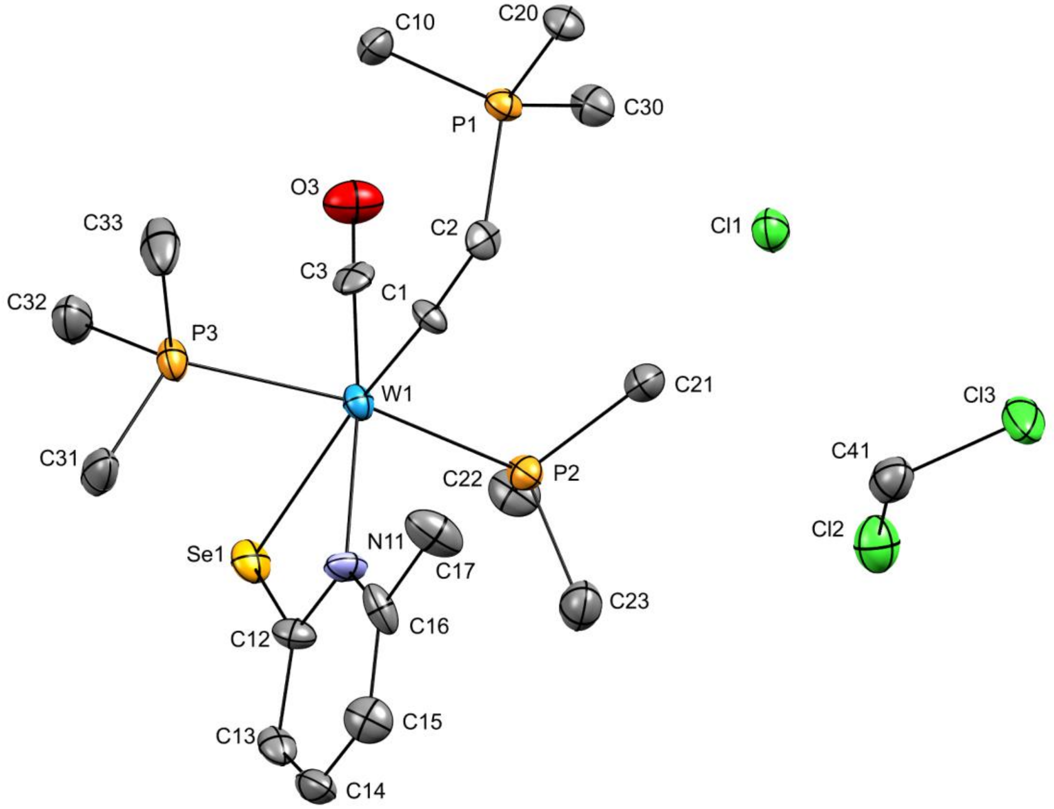

Figure S7. Molecular structure of the metal complex of $[WO(CCH_2PMe_3)(PMe_3)_2(6\text{-}MePySe)]Cl$ (**6**) showing the atomic numbering scheme. The probability ellipsoids are drawn at the 50% probability level. H atoms were omitted for clarity.

NMR Data

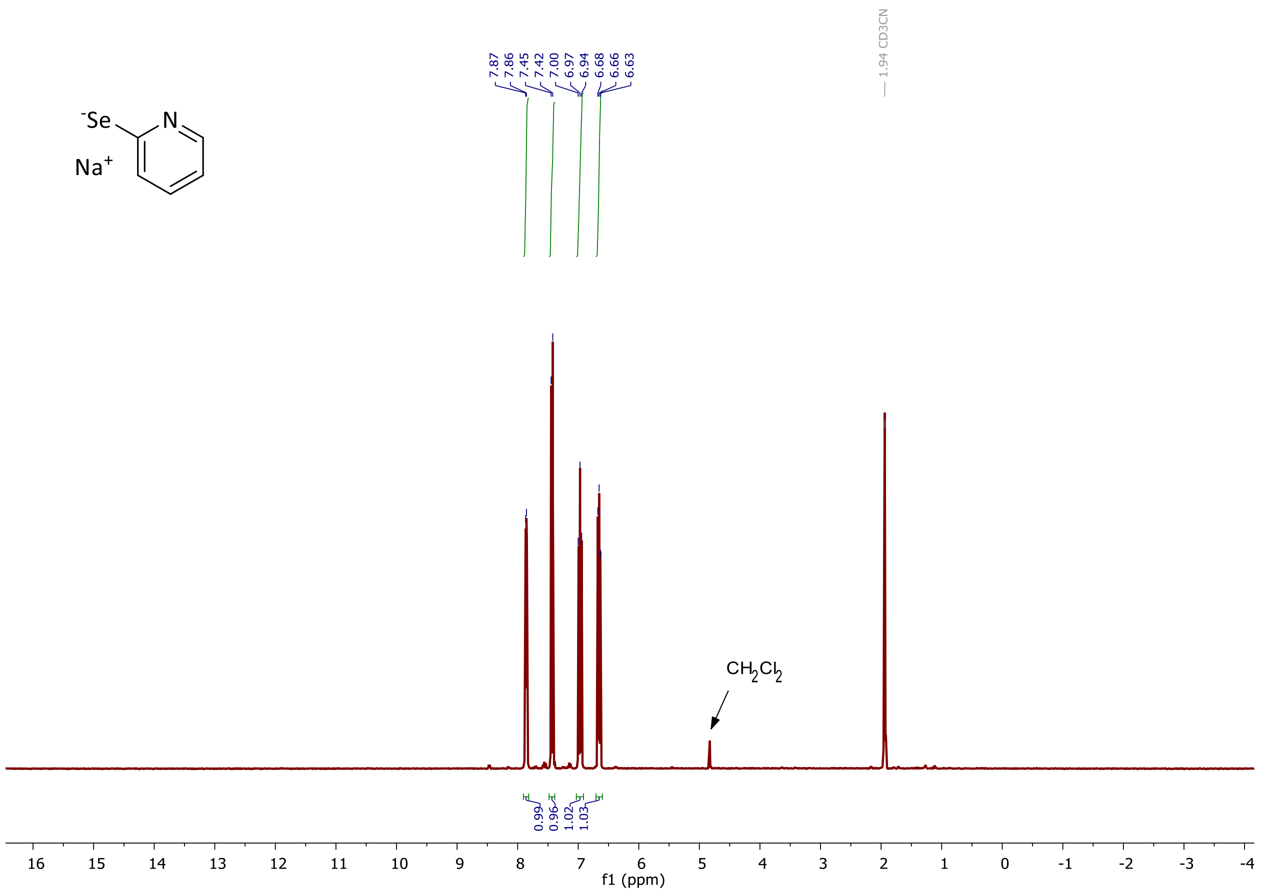

Figure S8. ^{1}H NMR spectrum of Na(PySe) in CD_3CN.

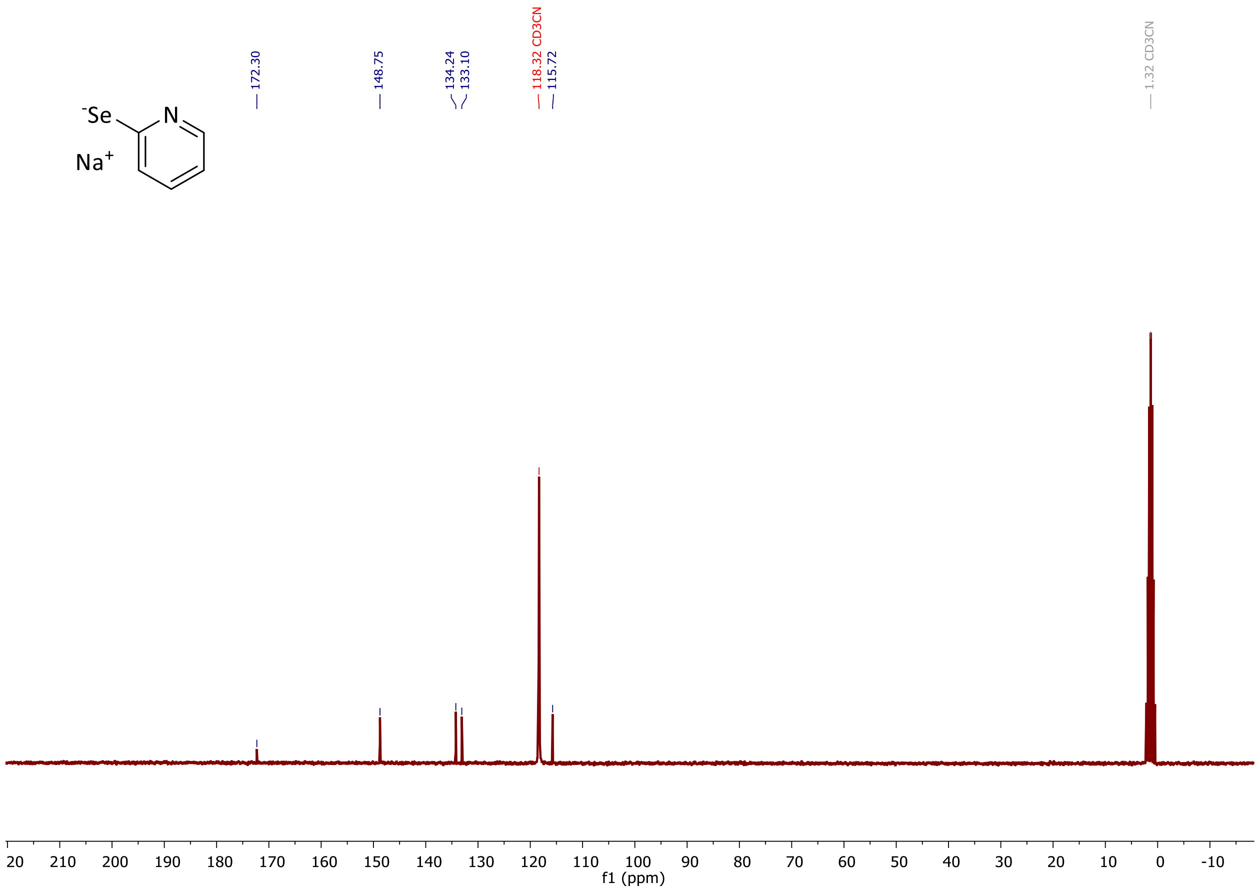

Figure S9. ^{13}C NMR spectrum of Na(PySe) in CD_3CN.

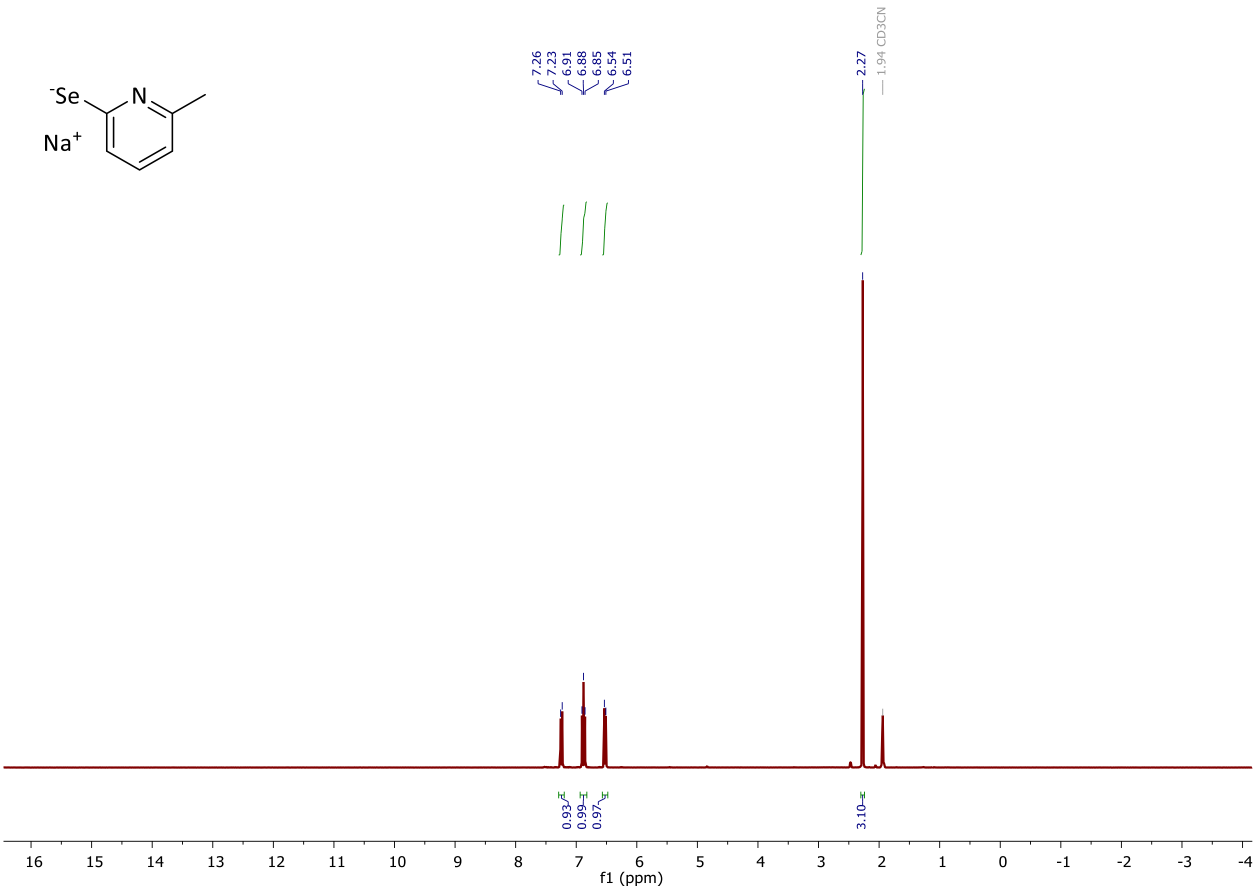

Figure S10. ^{1}H NMR spectrum of Na(6-MePySe) in CD_3CN.

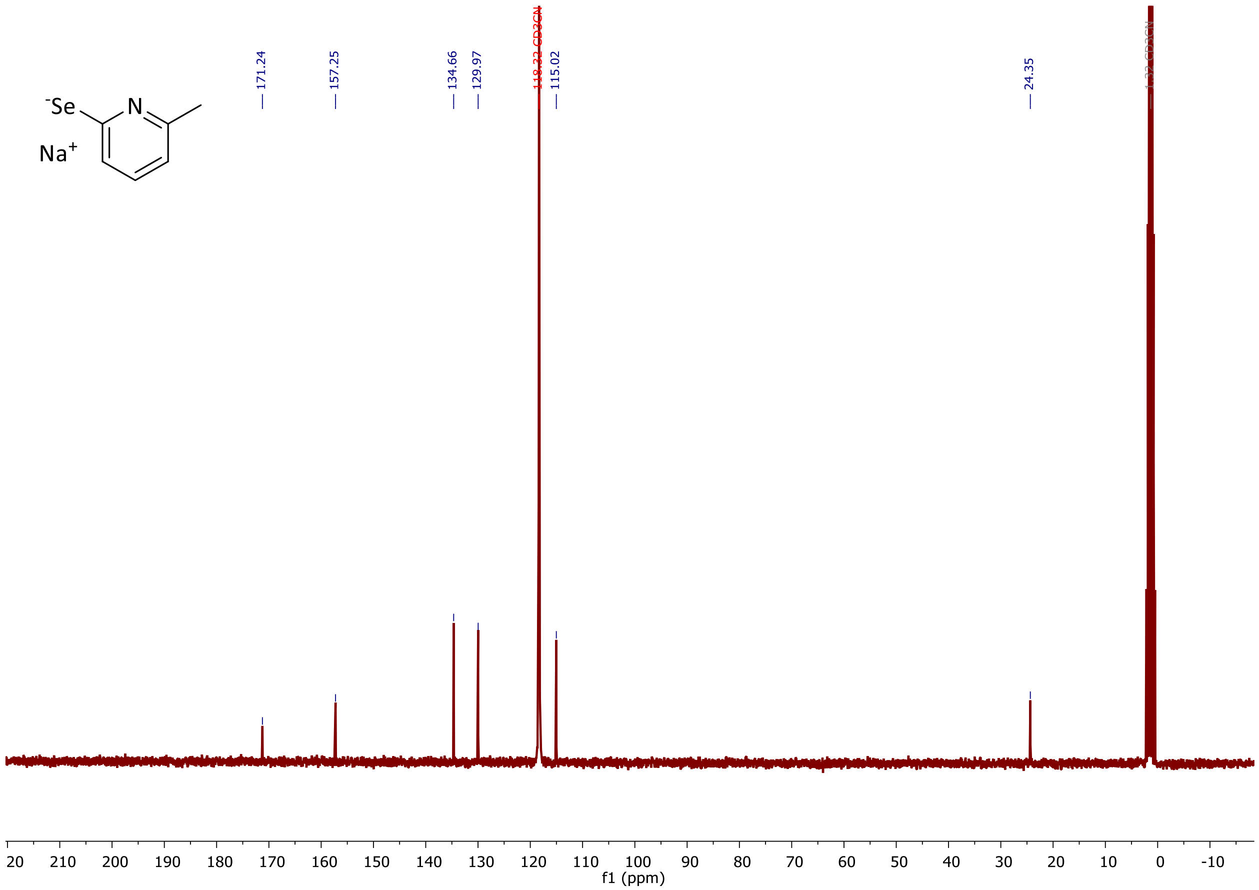

Figure S11. ^{13}C NMR spectrum of Na(6-MePySe) in CD_3CN.

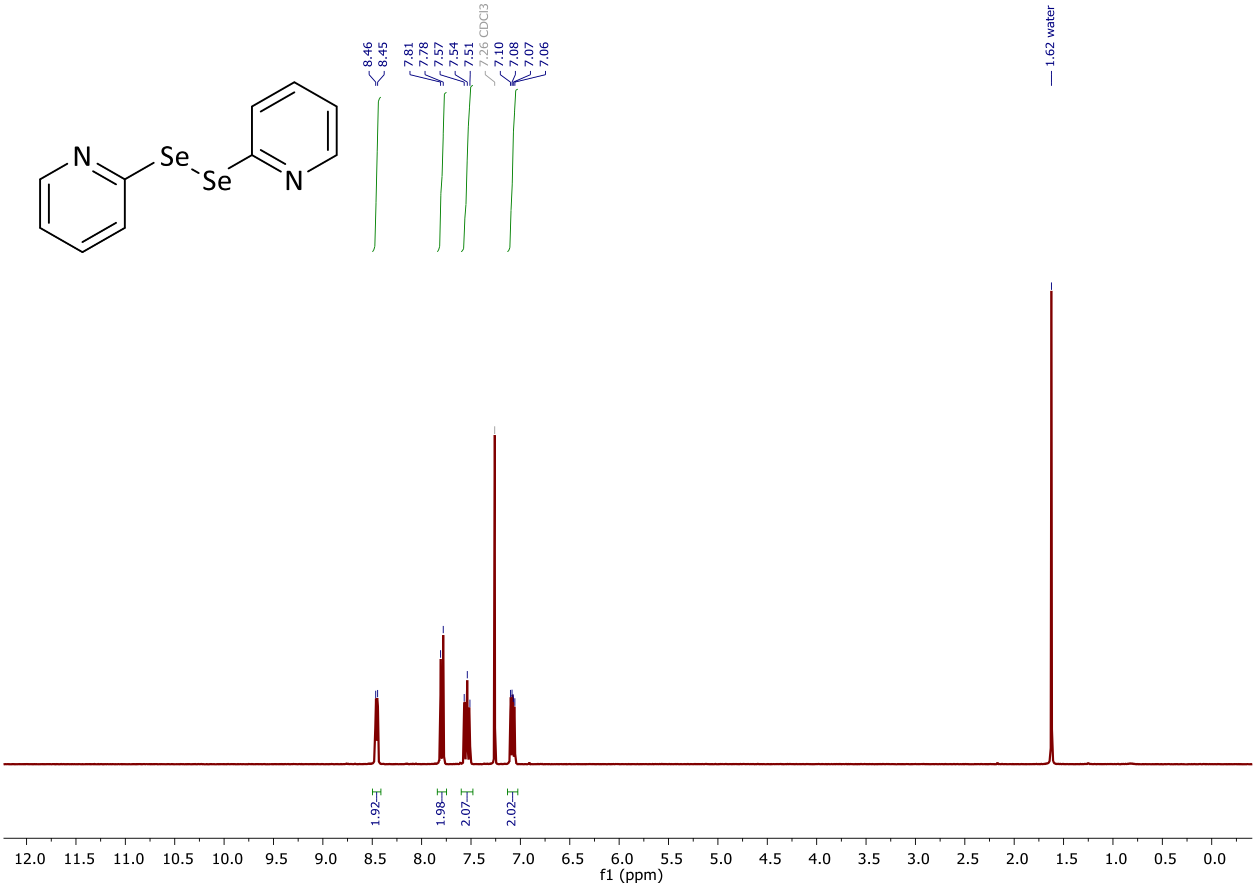

Figure S12. ^{1}H NMR spectrum of $(PySe)_2$ in $CDCl_3$.

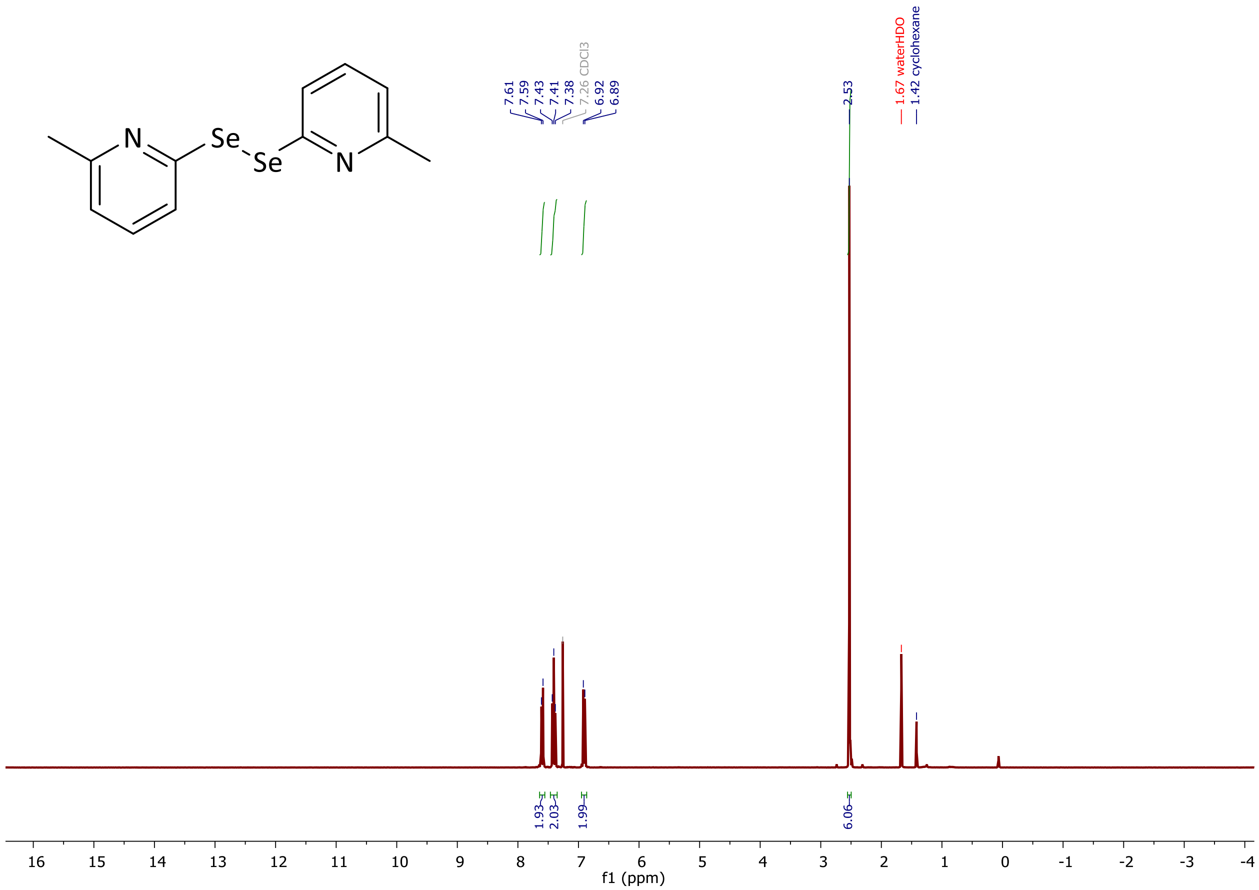

Figure S13. ^{1}H NMR spectrum of $(6\text{-MePySe})_2$ in $CDCl_3$.

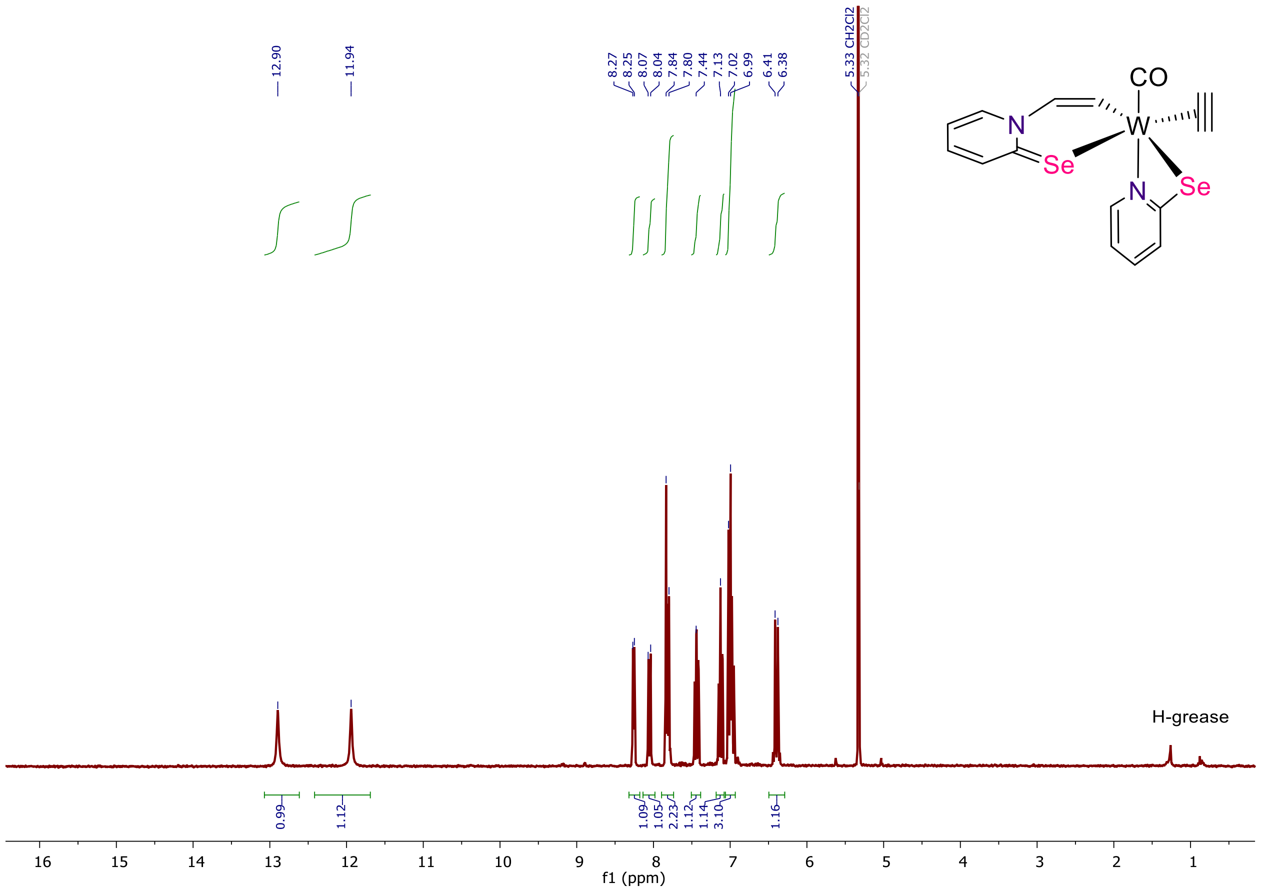

Figure S14. ^{1}H NMR spectrum of $[W(CO)(C_2H_2)(CHCH\text{-}PySe)(PySe)]$ (**2**) in CD_2Cl_2.

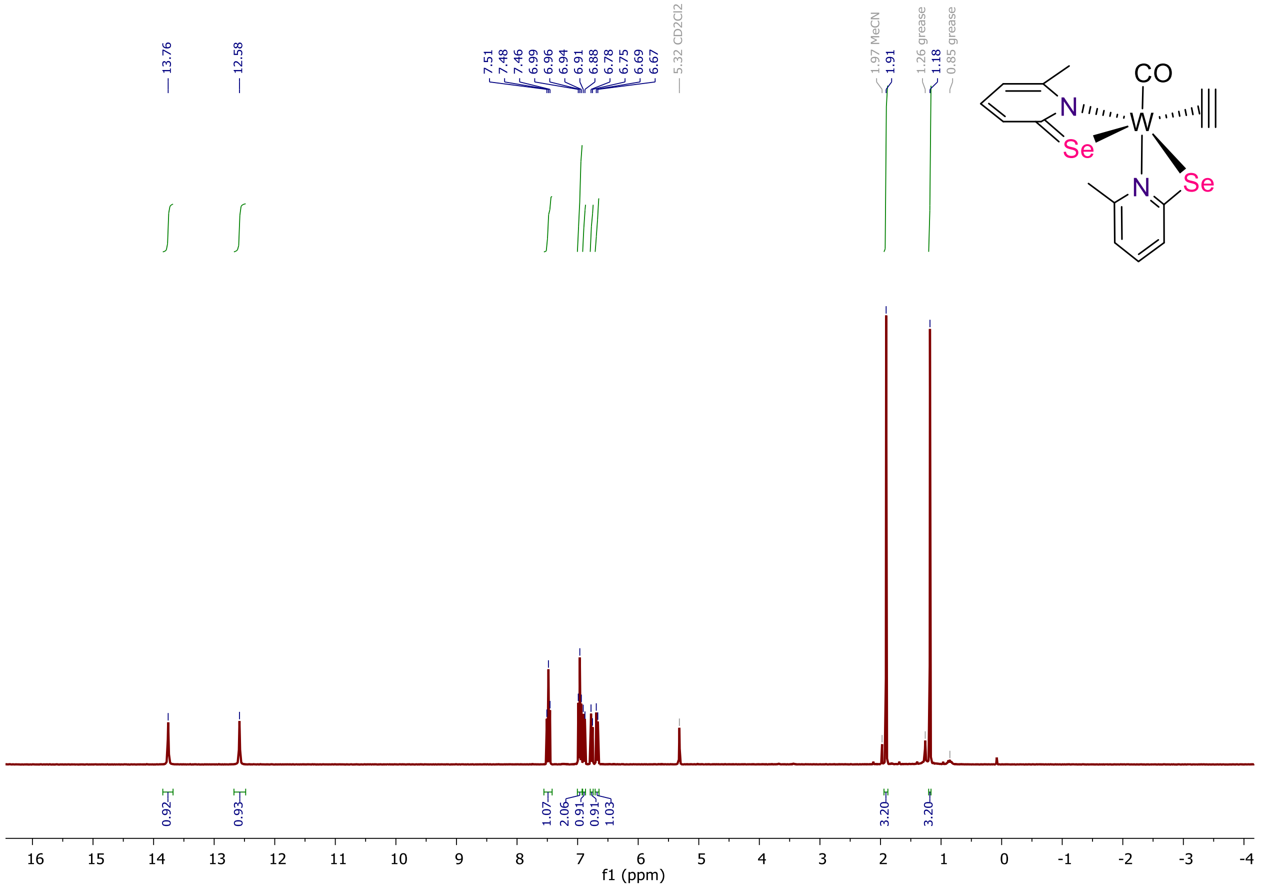

Figure S15. ^{1}H NMR spectrum of [W(CO)(C_2H_2)(6-Me-PySe)$_2$] (**3**) in CD_2Cl_2 before recrystallization.

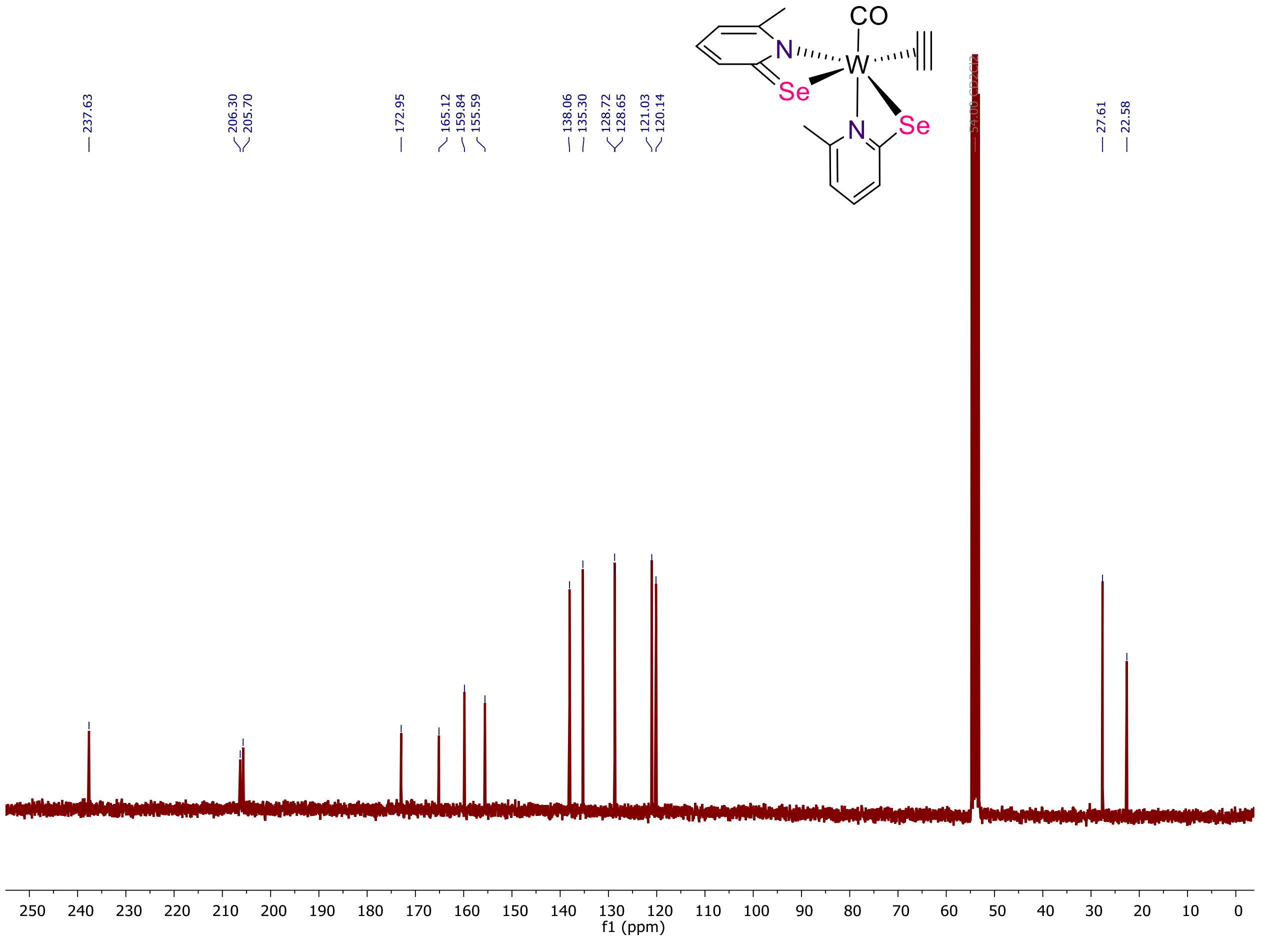

Figure S16. ^{13}C NMR spectrum of [W(CO)(C_2H_2)(6-Me-PySe)$_2$] (**3**) in CD_2Cl_2 before recrystallization.

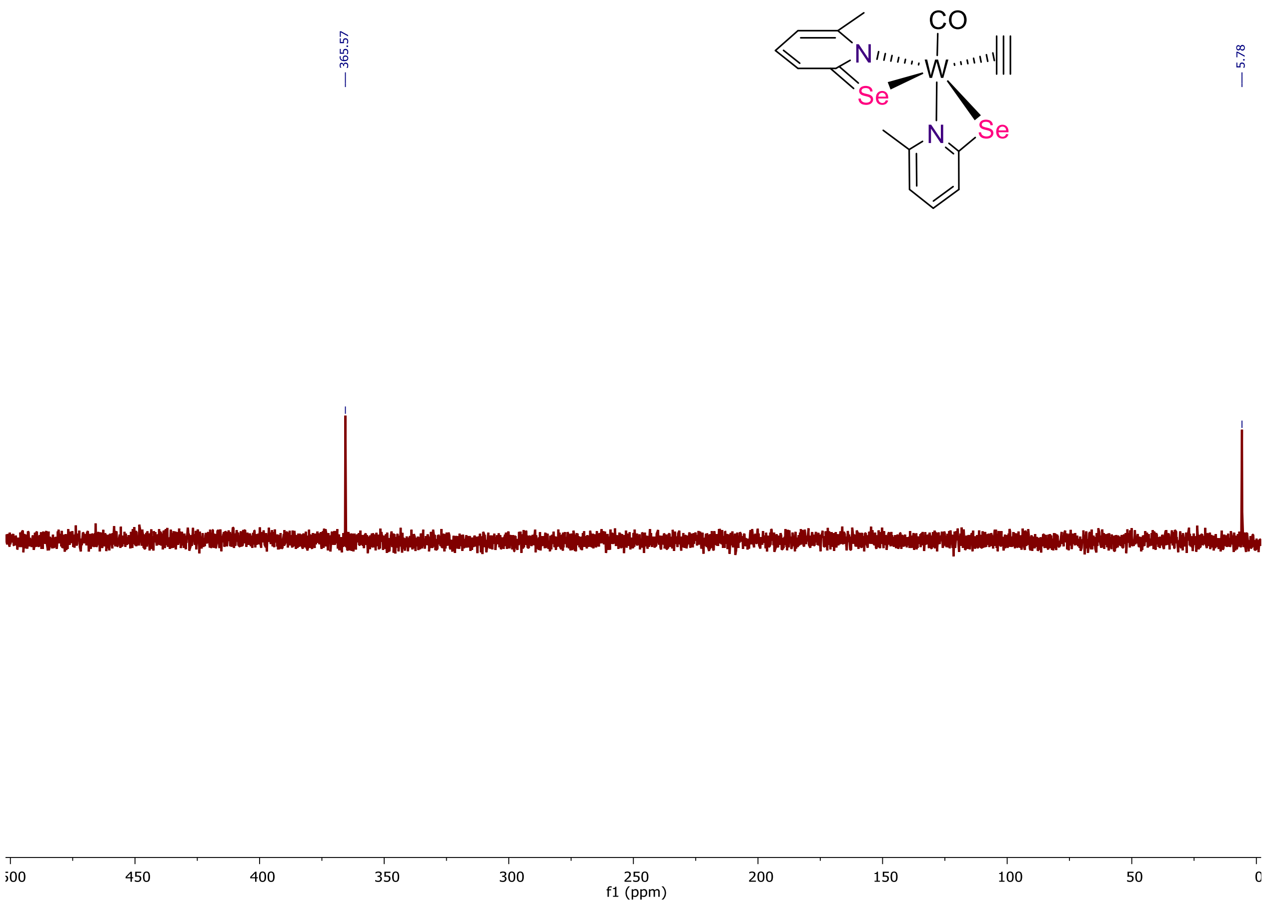

Figure S17. ^{77}Se NMR spectrum of [W(CO)(C_2H_2)(6-Me-PySe)$_2$] (**3**) in CD_2Cl_2.

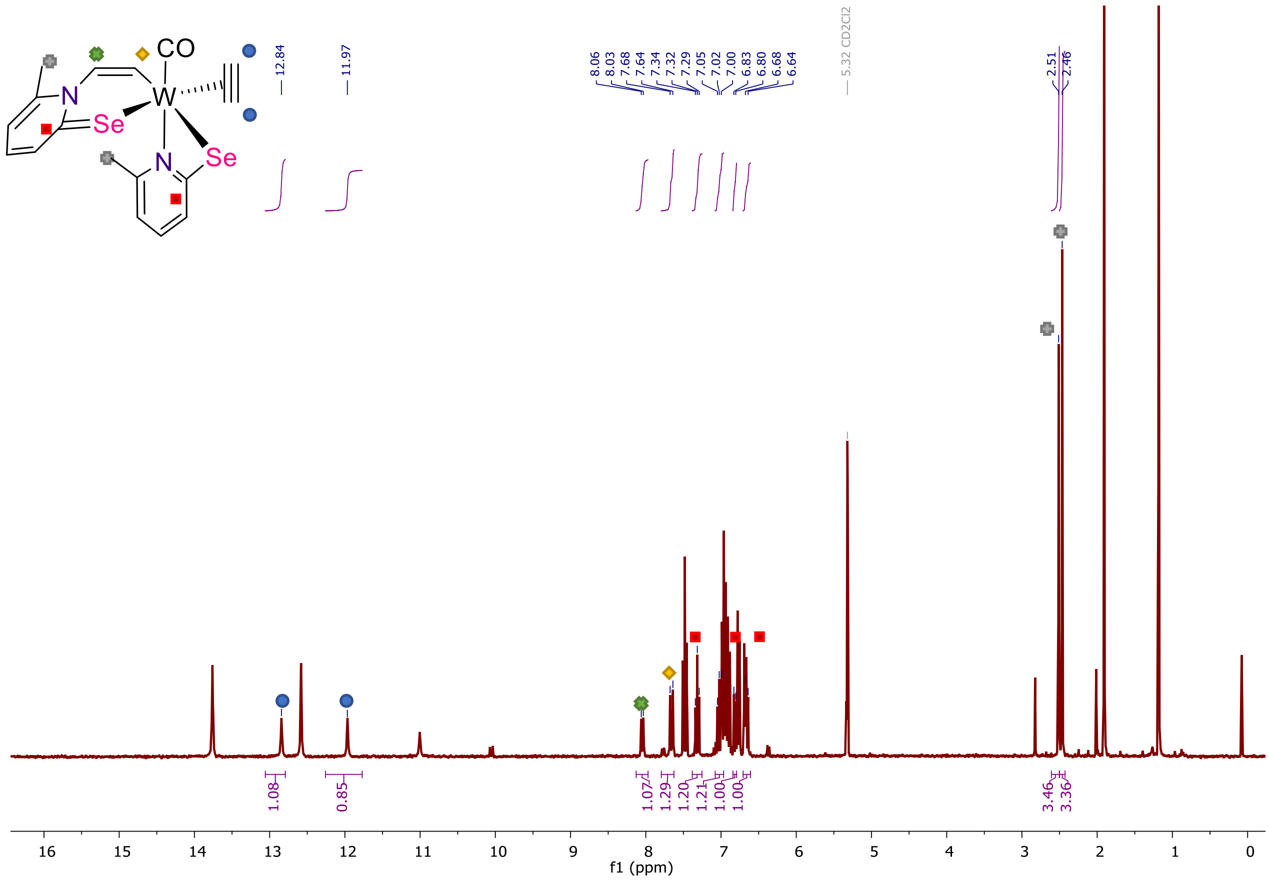

Figure S18. ^{1}H NMR spectrum of [W(CO)(C_2H_2)(CHCH-6-MePySe)(6-MePySe)] (**5**) in CD_2Cl_2. Chemical shifts and corresponding integrals are obtained by subtracting the main impurity which is unreacted [W(CO)(C_2H_2)(6-Me-PySe)$_2$] (**3**).

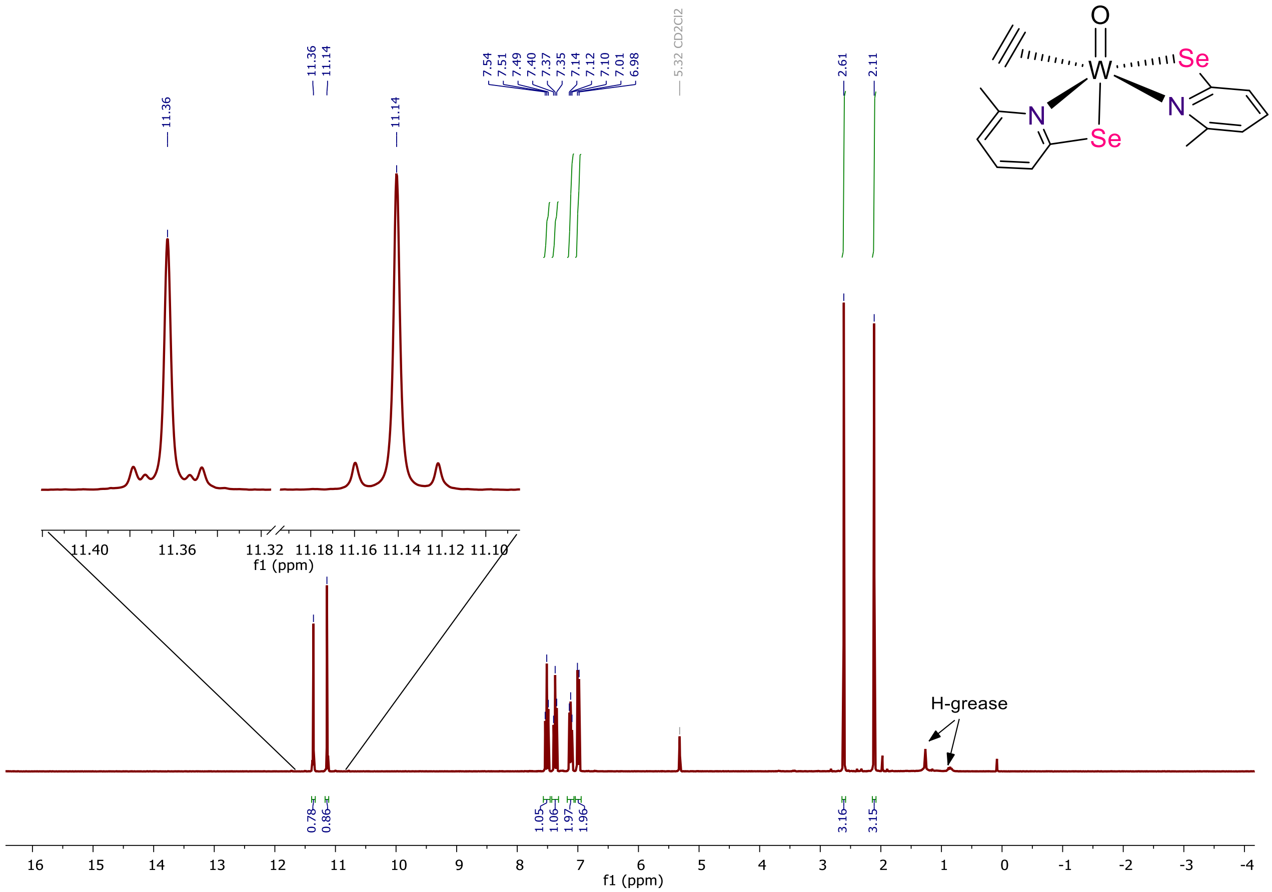

Figure S19. ^{1}H NMR spectrum of [$WO(C_2H_2)(6\text{-Me-PySe})_2$] (**4**) in CD_2Cl_2.

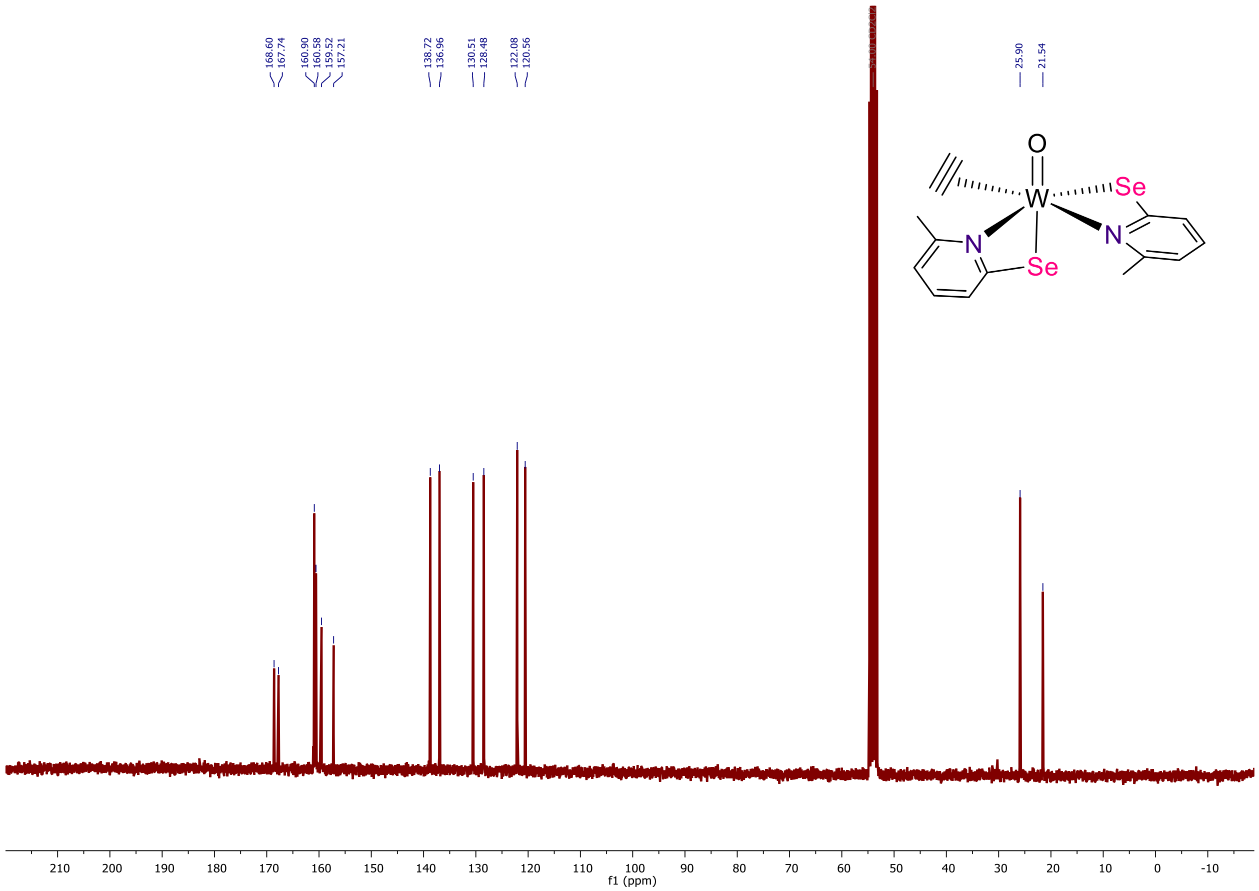

Figure S20. ^{13}C NMR spectrum of [WO(C_2H_2)(6-Me-PySe)$_2$] (**4**) in CD_2Cl_2.

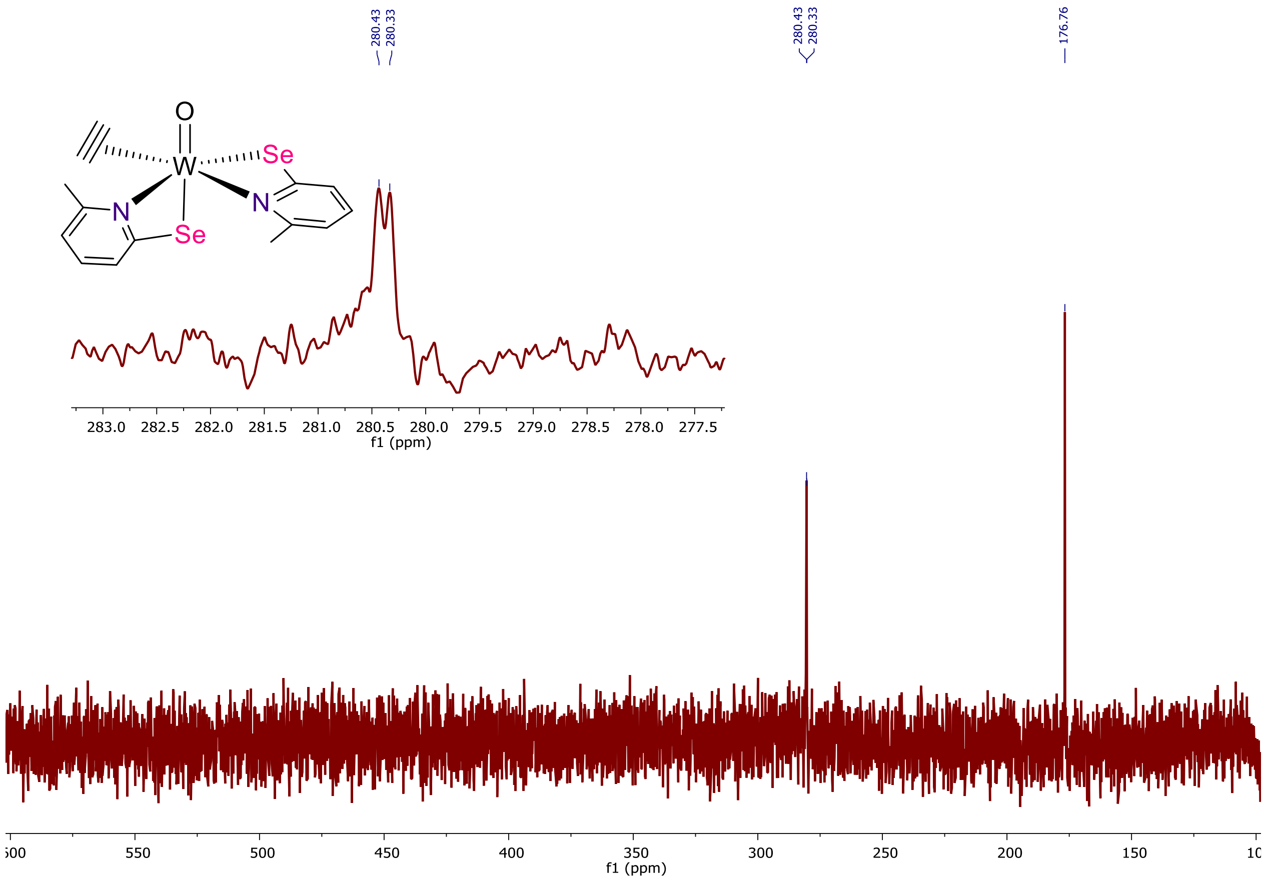

Figure S21. ^{77}Se NMR spectrum of $[WO(C_2H_2)(6\text{-Me-PySe})_2]$ (**4**) in CD_2Cl_2.

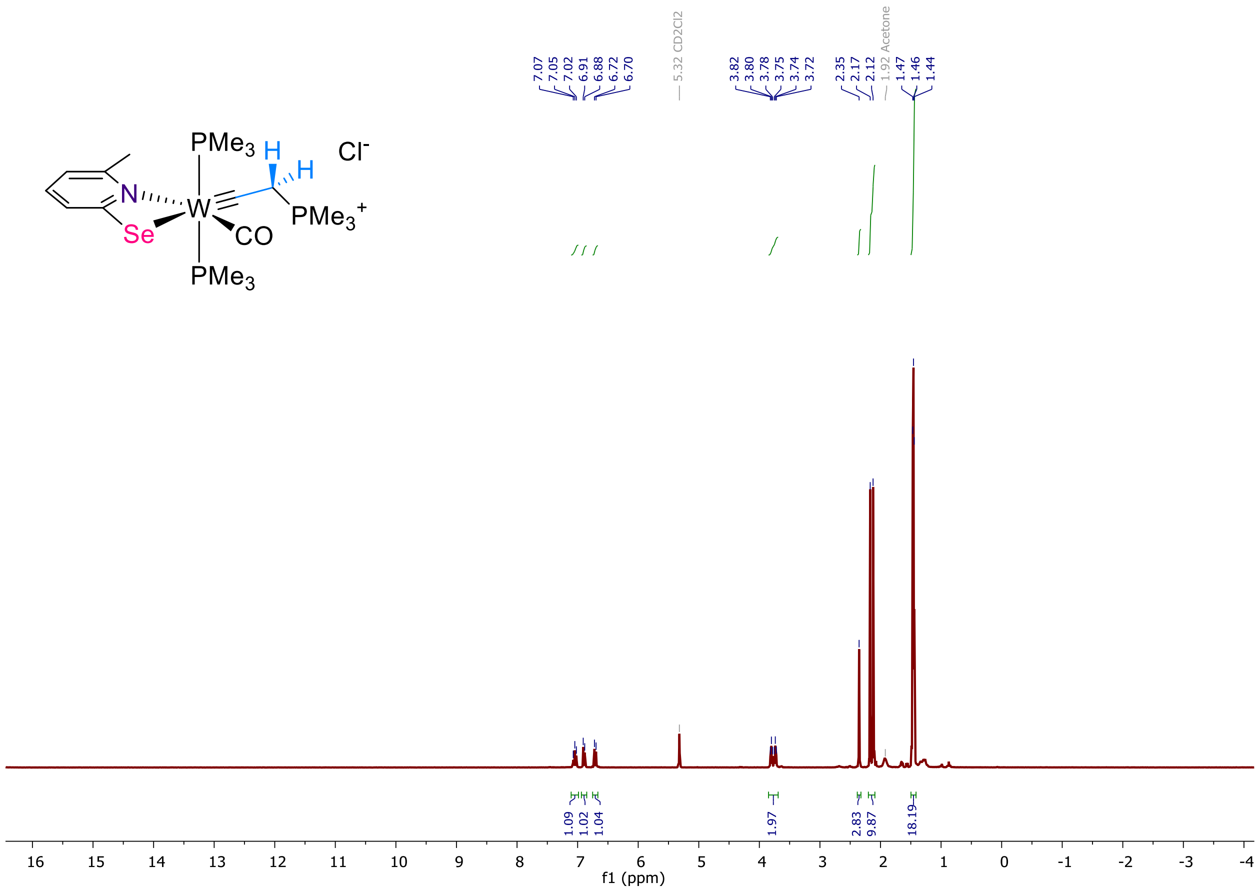

Figure S22. ^{1}H NMR spectrum of [W(CO)(CCH$_2$PMe$_3$)(PMe$_3$)$_2$(6-Me-PySe)]Cl (**6**) in CD_2Cl_2.

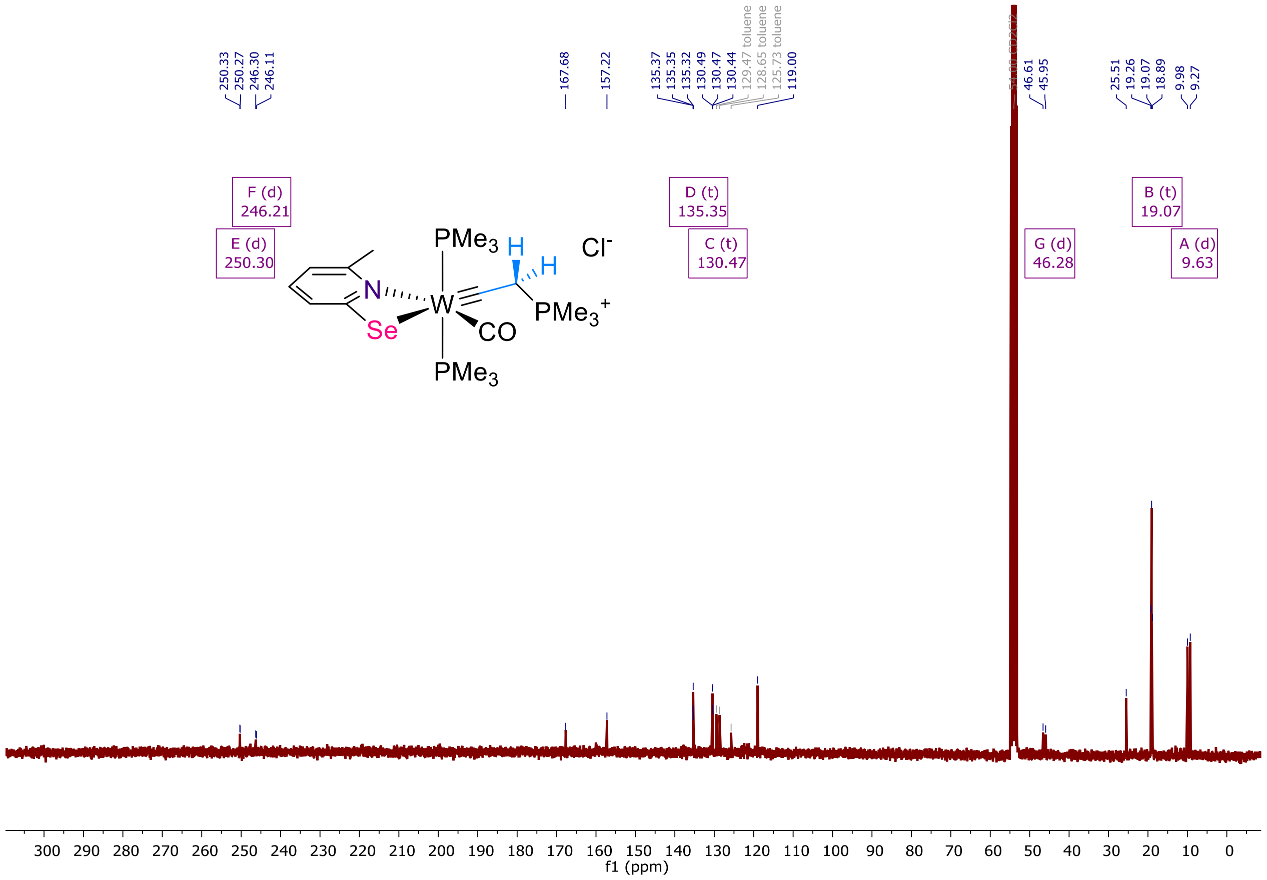

Figure S23. ^{13}C NMR spectrum of $[W(CO)(CCH_2PMe_3)(PMe_3)_2(6\text{-Me-PySe})]Cl$ (**6**) in CD_2Cl_2. Extra signals originate from toluene and are labelled as such.

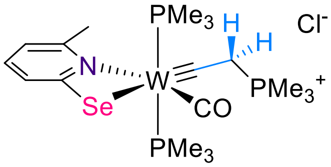

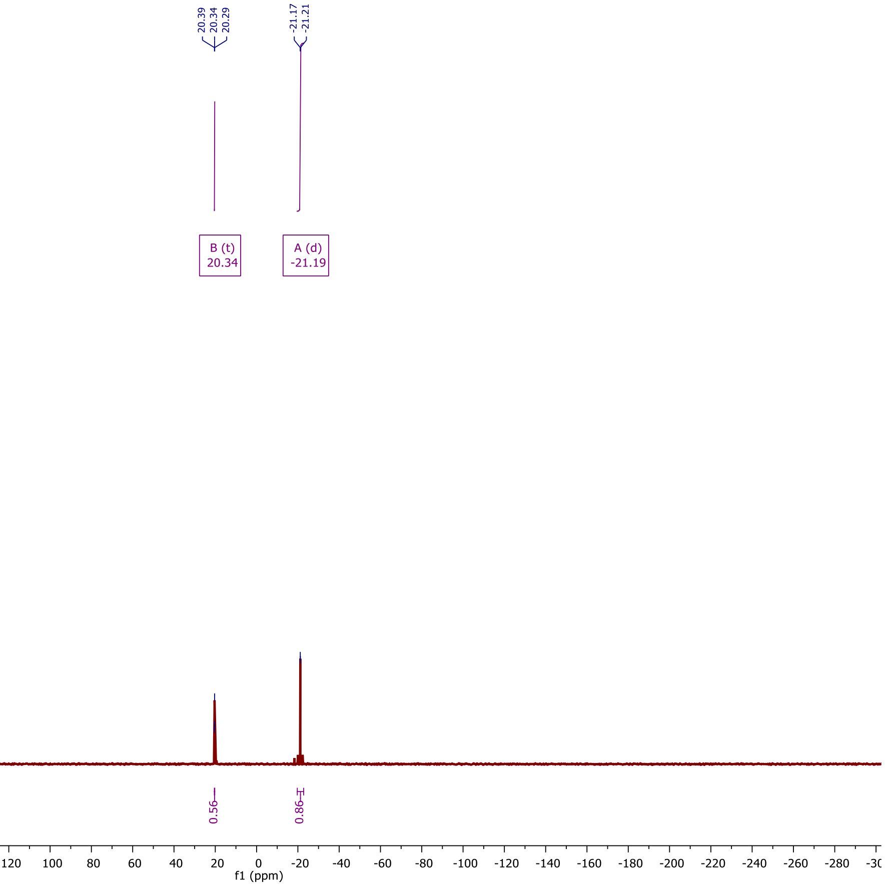

Figure S24. ^{31}P NMR spectrum of [W(CO)(CCH$_2$PMe$_3$)(PMe$_3$)$_2$(6-Me-PySe)]Cl (**6**) in CD$_2$Cl$_2$.

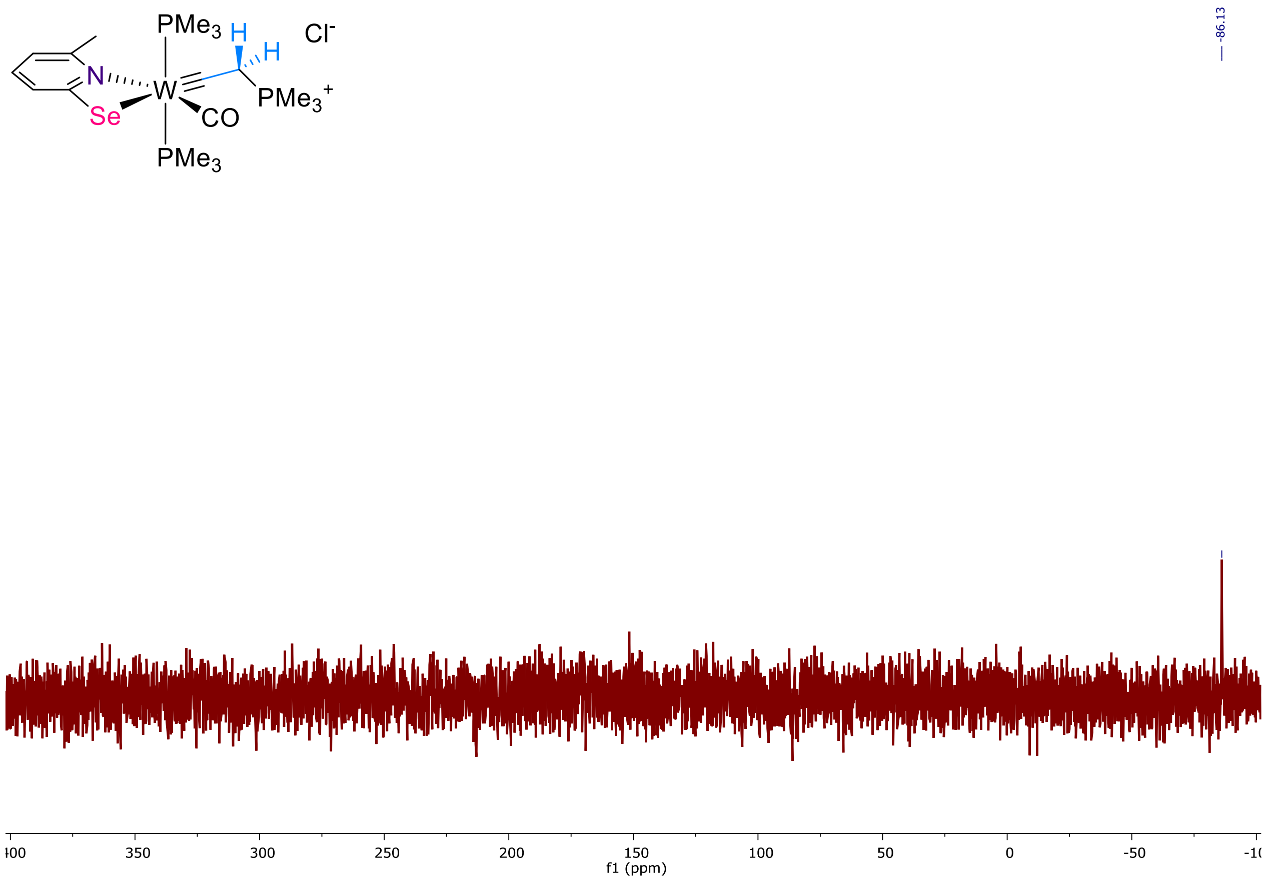

Figure S25. ^{77}Se NMR spectrum of $[W(CO)(CCH_2PMe_3)(PMe_3)_2(6\text{-}Me\text{-}PySe)]Cl$ (**6**) in CD_2Cl_2.

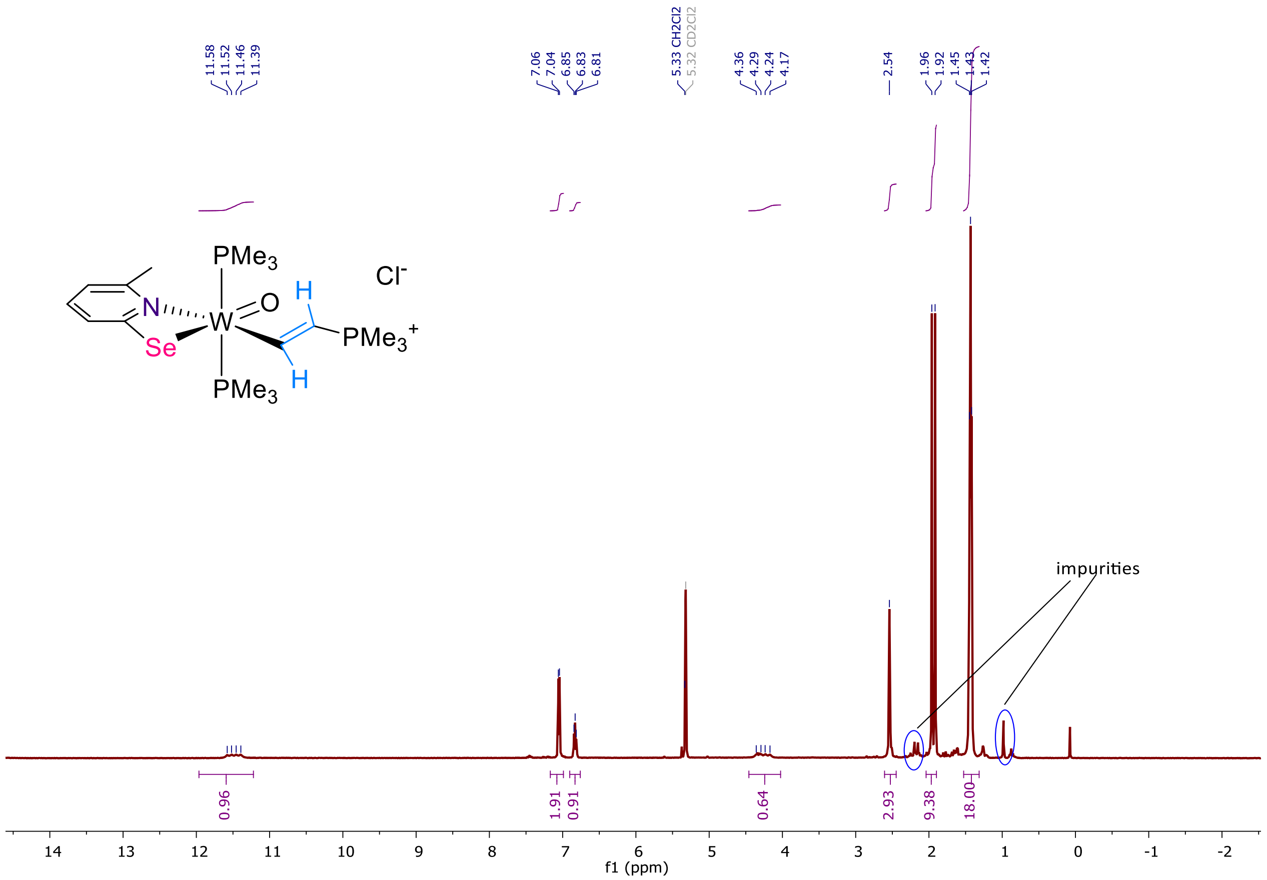

Figure S26. ^{1}H NMR of $[WO(CCH_2PMe_3)(PMe_3)_2(6\text{-Me-PySe})]Cl$ (**7**) in CD_2Cl_2.

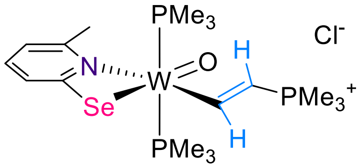

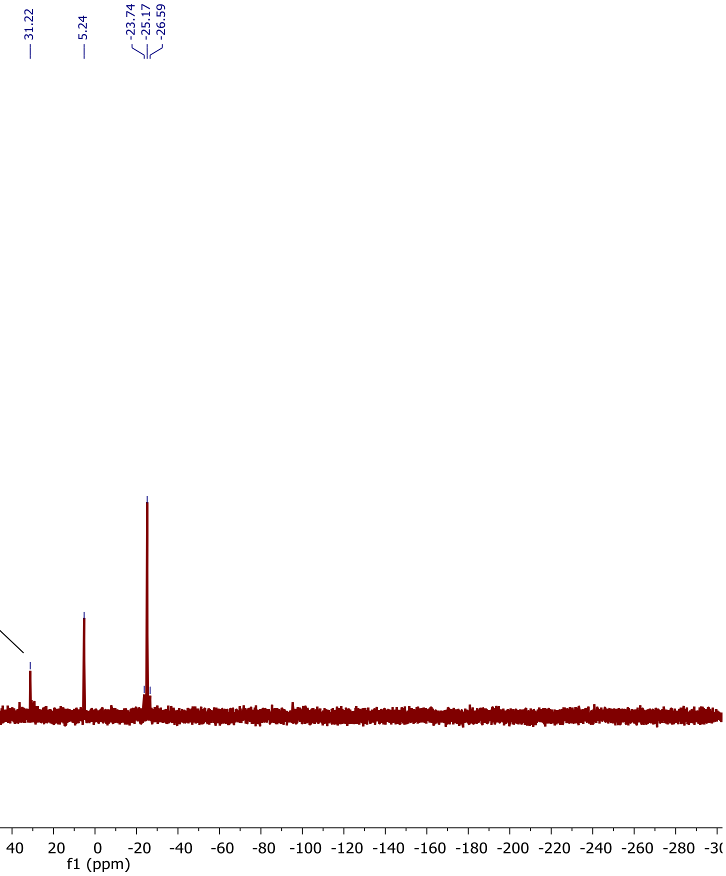

Figure S27. ^{31}P NMR spectrum of [WO(CCH$_2$PMe$_3$)(PMe$_3$)$_2$(6-Me-PySe)]Cl (**7**) in CD_2Cl_2.

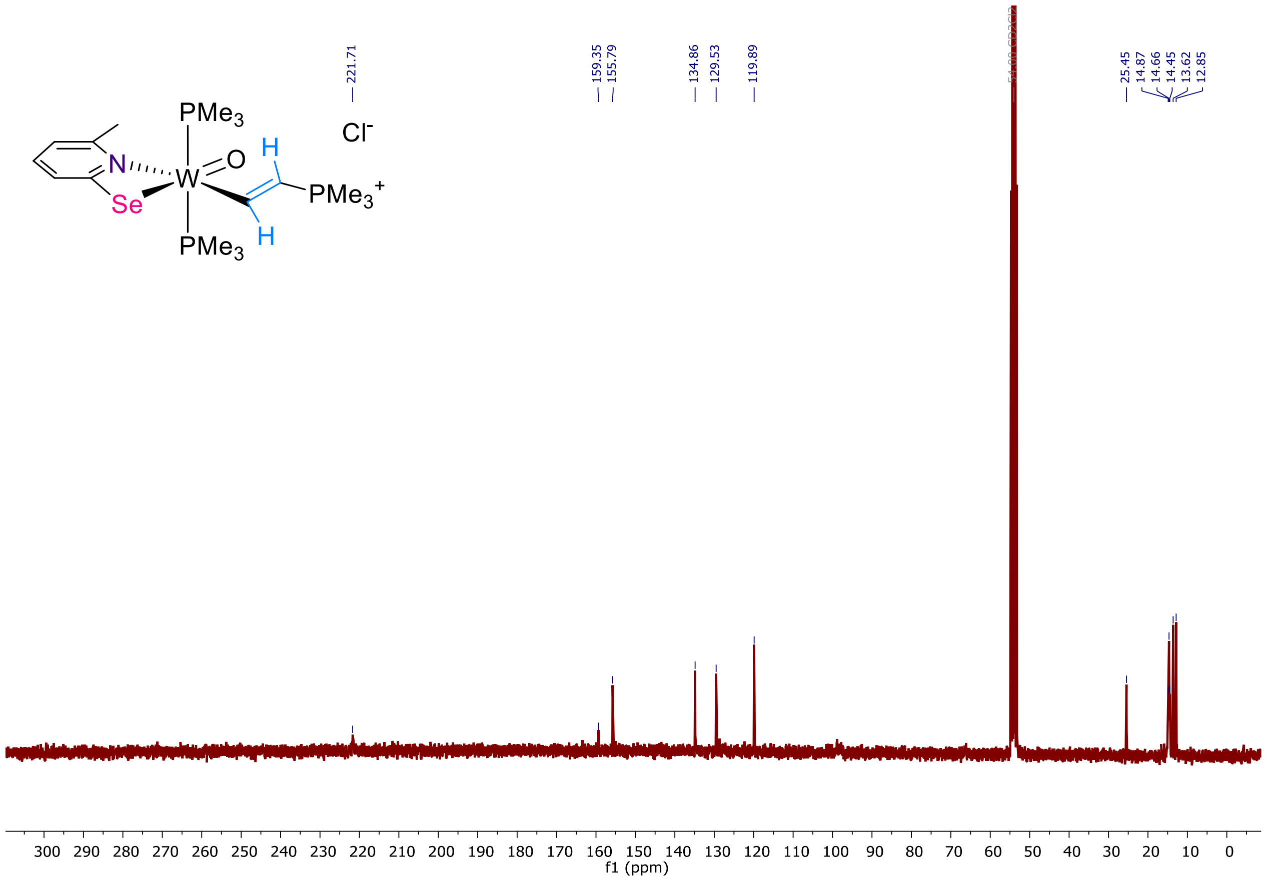

Figure S28. ^{13}C NMR spectrum of [WO(CCH$_2$PMe$_3$)(PMe$_3$)$_2$(6-Me-PySe)]Cl (**7**) in CD$_2$Cl$_2$.

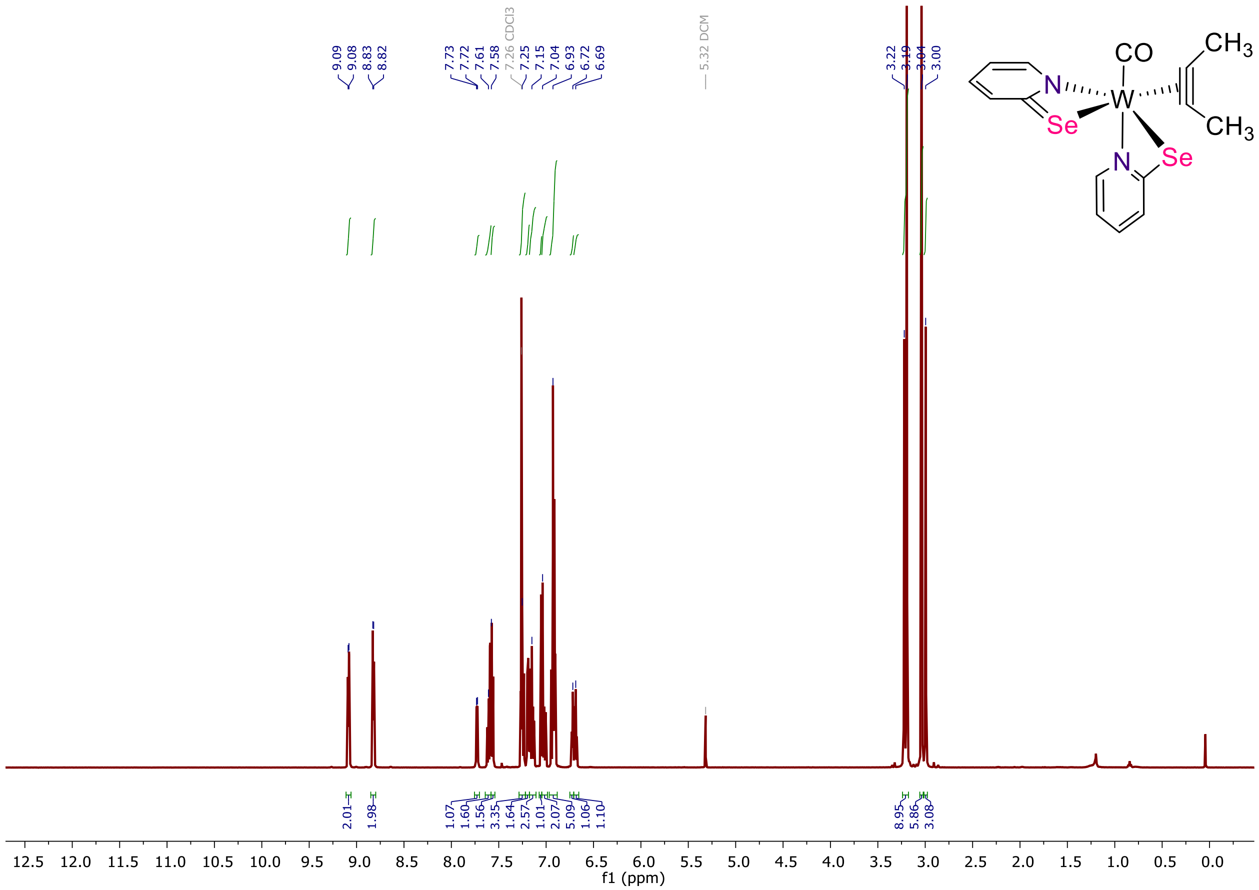

Figure S29. ^{1}H NMR spectrum of $[W(CO)(MeCCMe)(PySe)_2]$ (**8**) at -30 °C in $CDCl_3$.

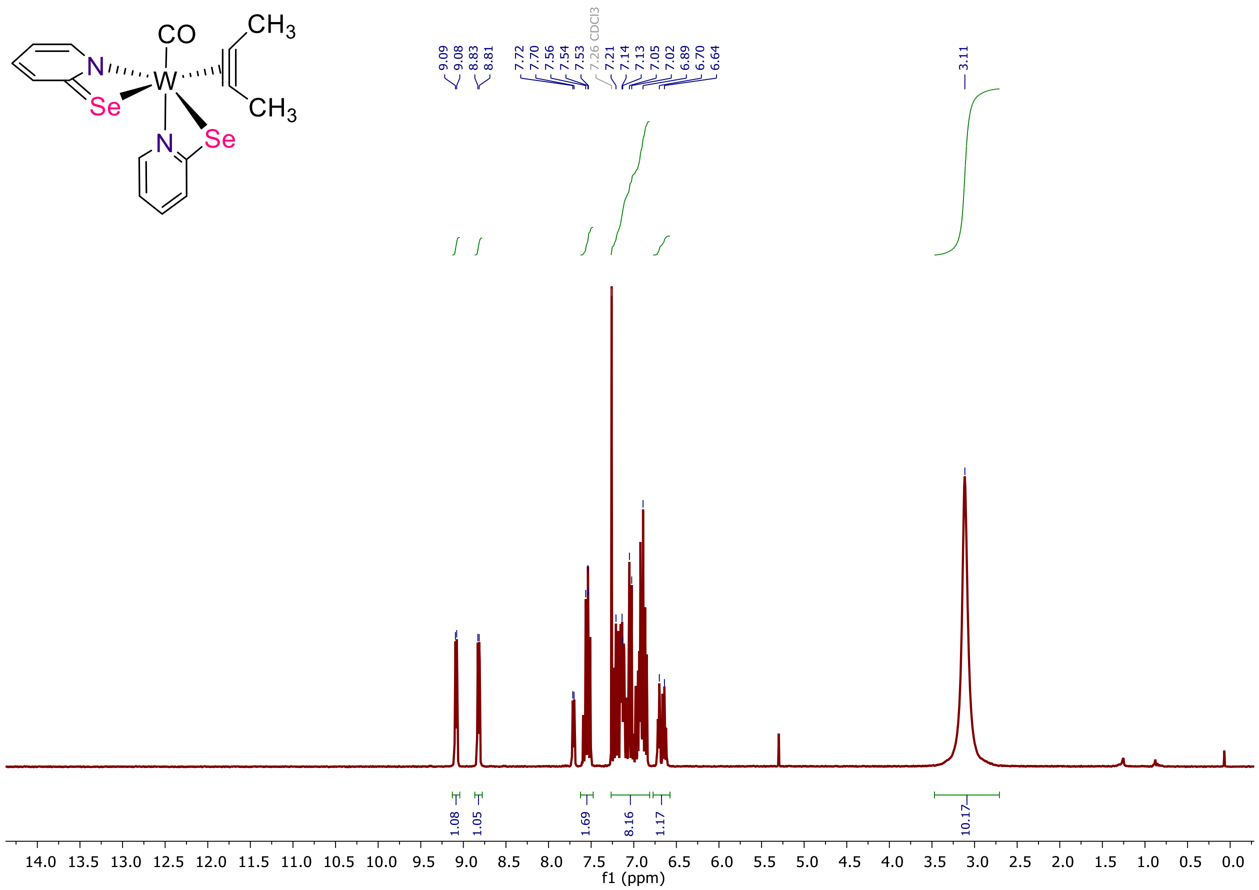

Figure S30. ^{1}H NMR spectrum of [W(CO)(MeCCMe)(PySe)$_2$] (**8**) at 22 °C in $CDCl_3$.

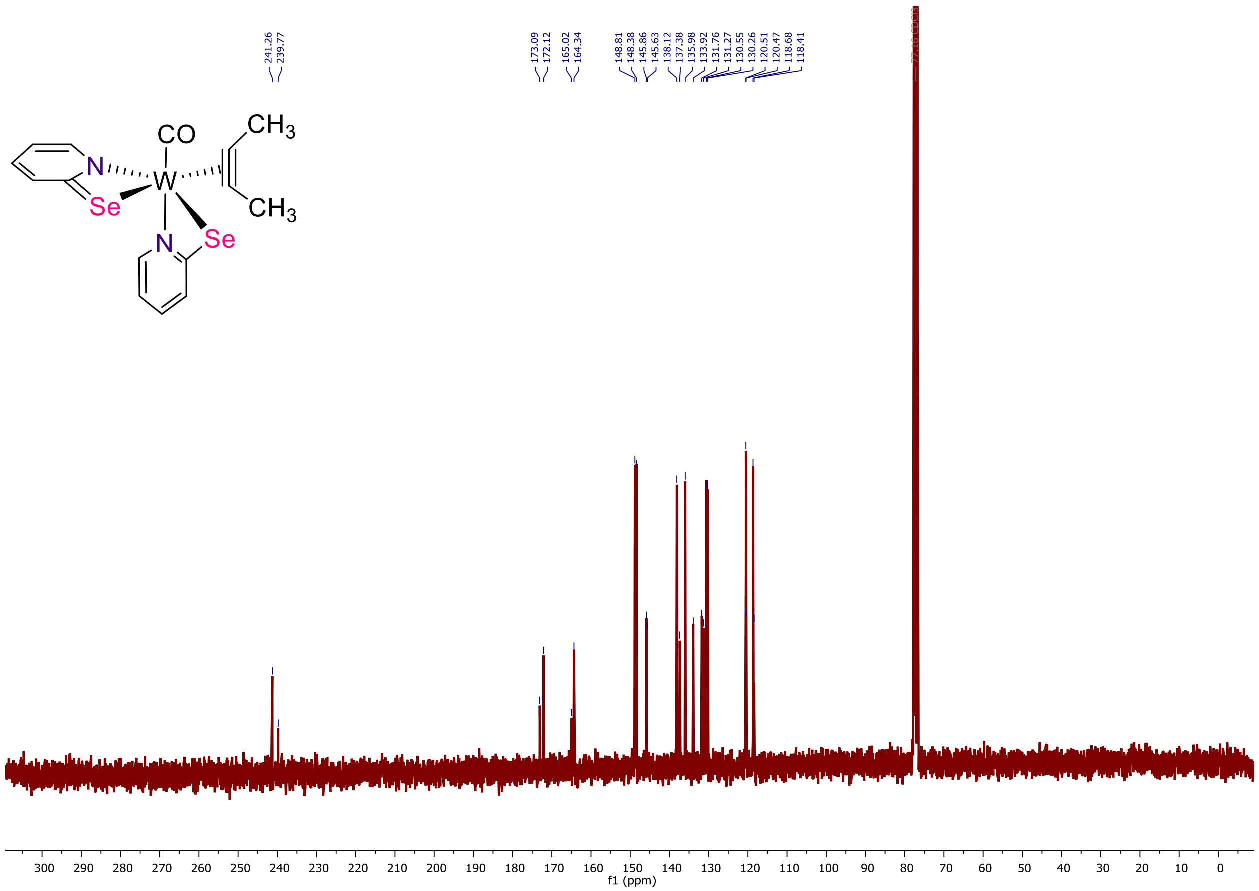

Figure S31. ^{13}C NMR spectrum of [W(CO)(MeCCMe)(PySe)$_2$] (**8**) at 22 °C in $CDCl_3$.

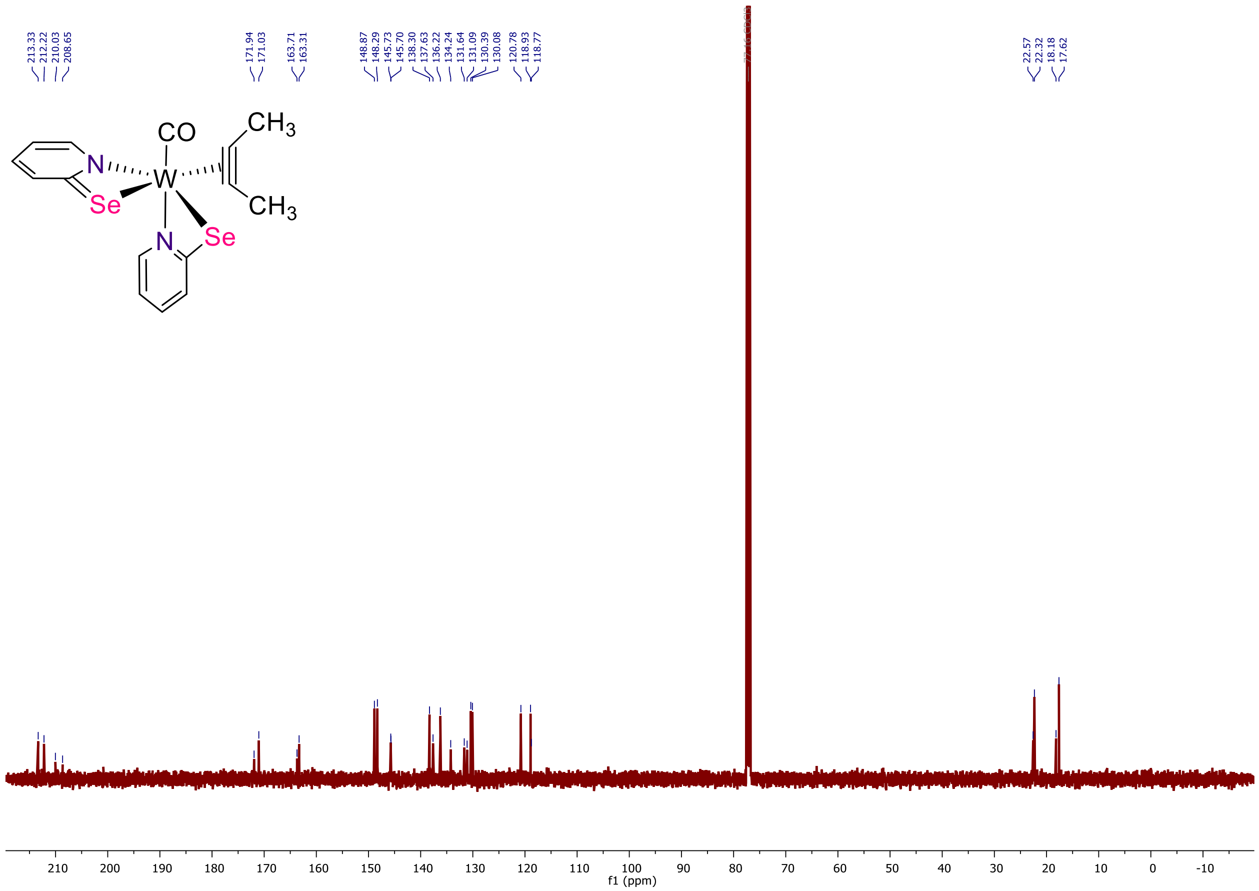

Figure S32. ^{13}C NMR spectrum of [W(CO)(MeCCMe)(PySe)$_2$] (**8**) at -30 °C in $CDCl_3$.

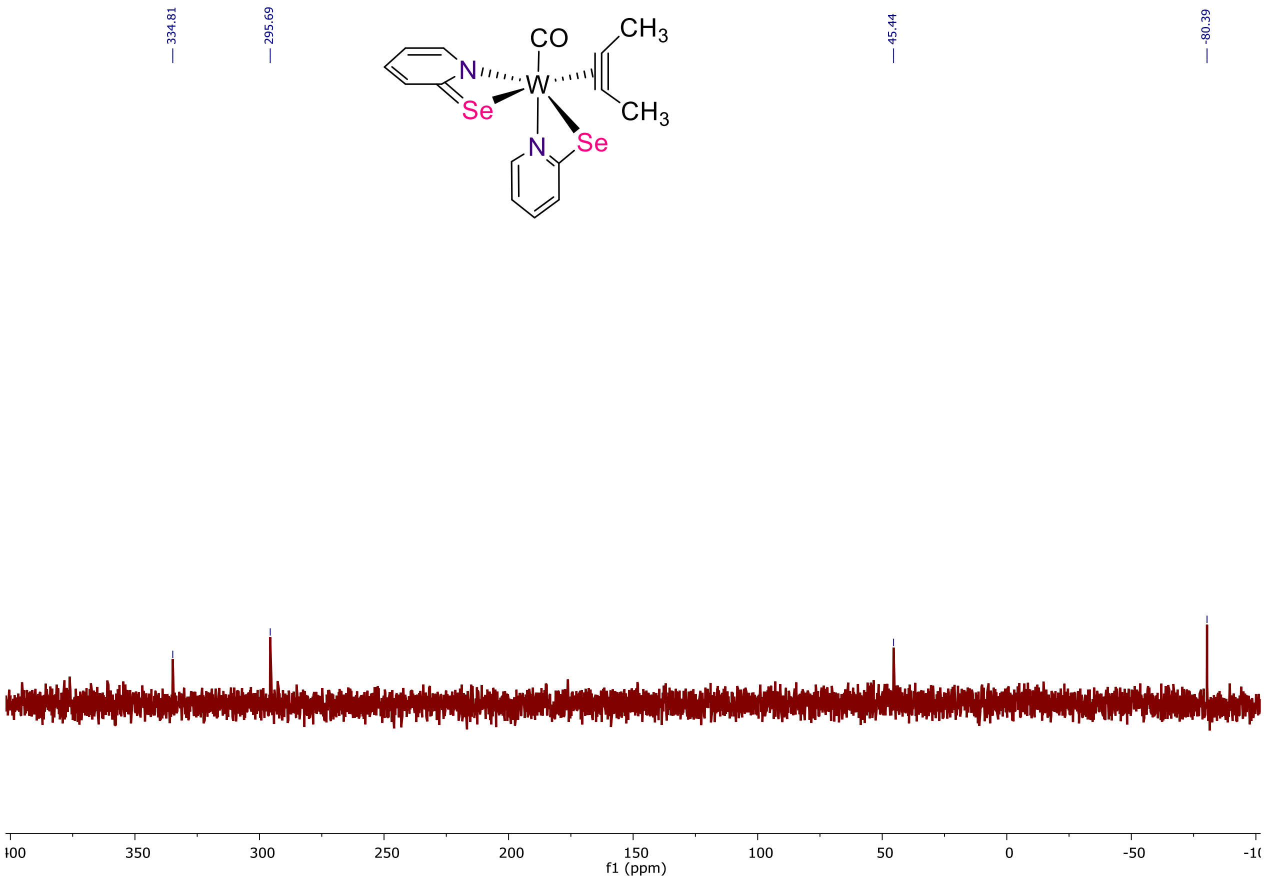

Figure S33. ^{77}Se NMR spectrum of [W(CO)(MeCCMe)(PySe)$_2$] (**8**) $CDCl_3$.

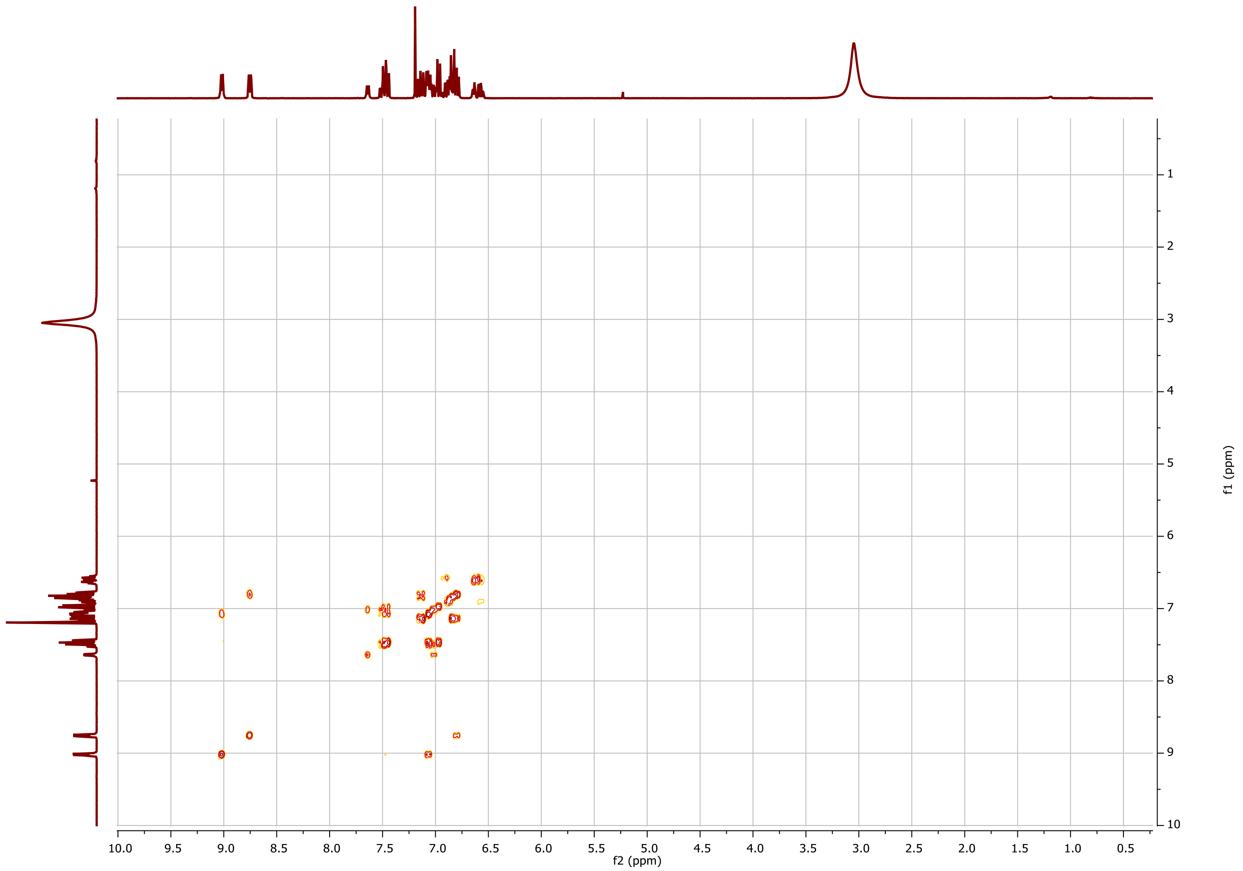

Figure S34. COSY NMR spectrum of $[W(CO)(MeCCMe)(PySe)_2]$ (**8**) at 22 °C in $CDCl_3$.

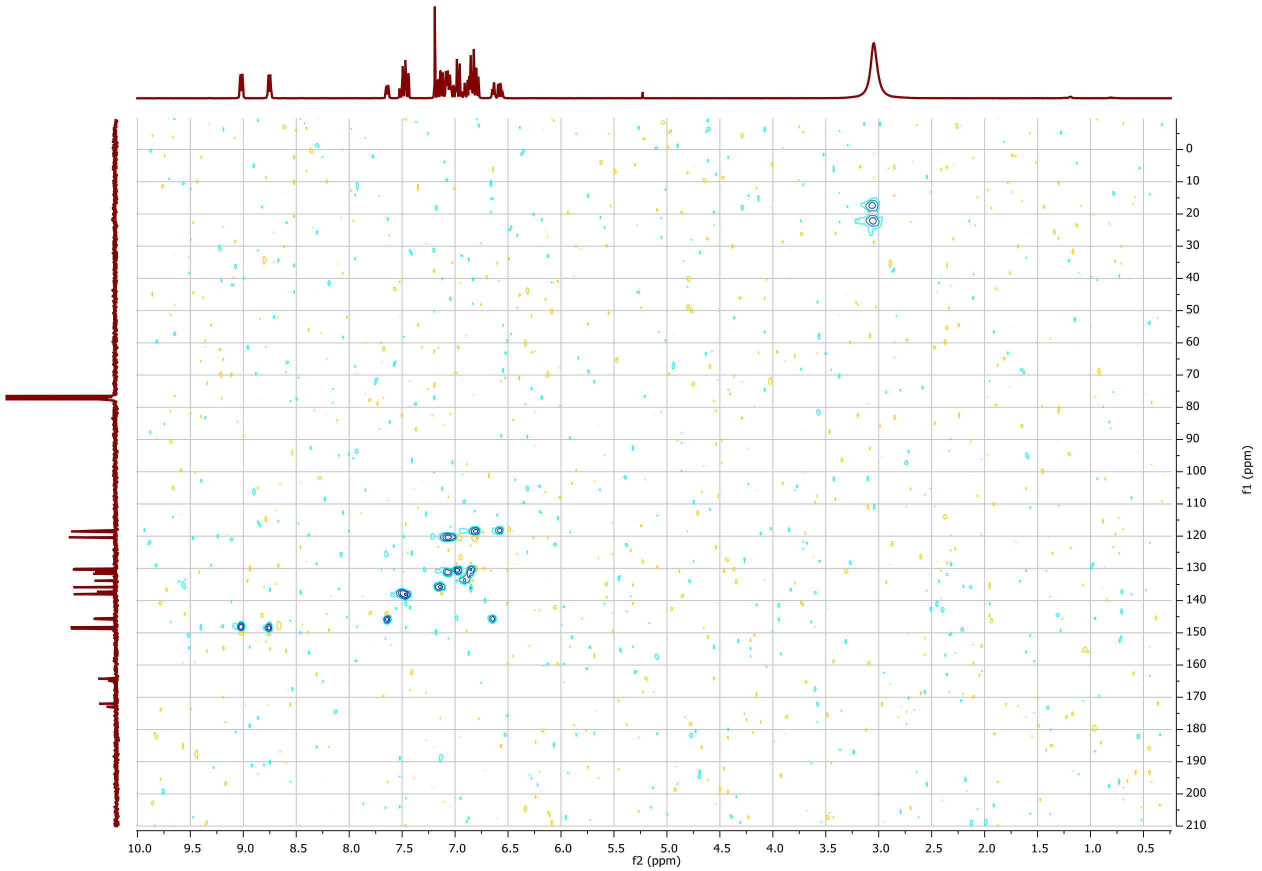

Figure S35. HSQC NMR spectrum of [W(CO)(MeCCMe)(PySe)$_2$] (**8**) at 22 °C in $CDCl_3$.

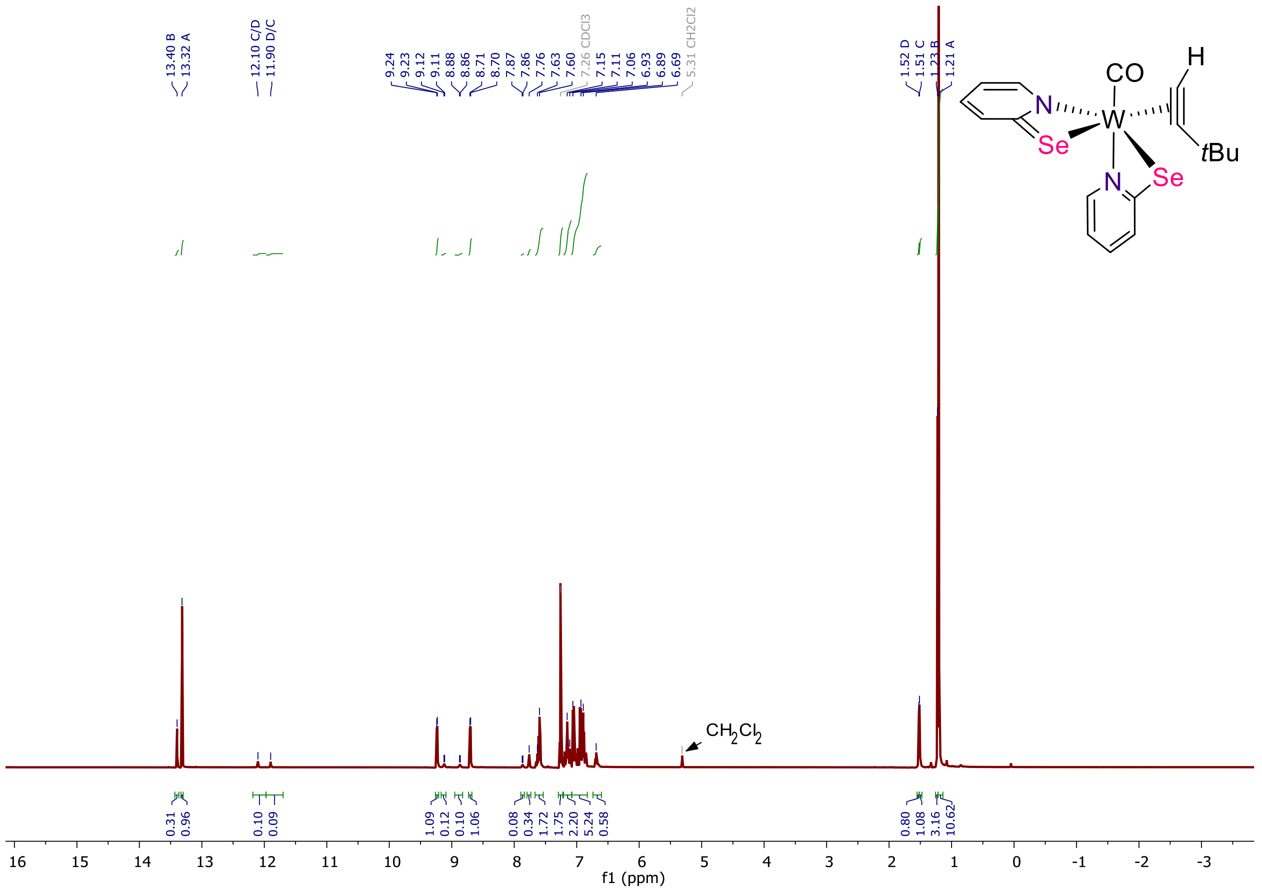

Figure S36. ^{1}H NMR spectrum of [W(CO)(*t*Bu-CCH)(PySe)$_2$] (**9**) in $CDCl_3$ at -30 °C. Isomers are labelled with A, B, C and D, where assignment is feasible. Labels with C/D or D/C are exclusively C and D, or D and C.

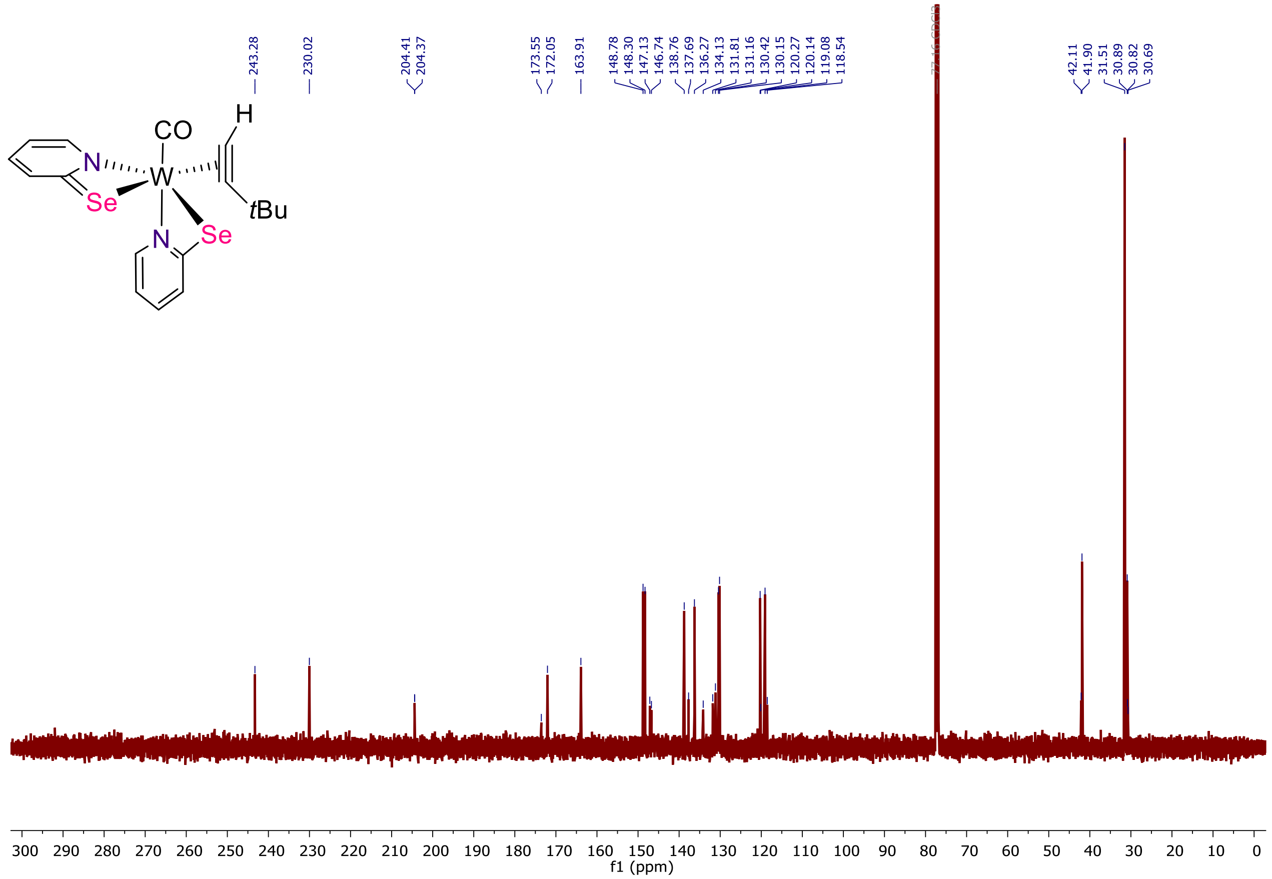

Figure S37. ^{13}C NMR spectrum of [W(CO)(*t*Bu-CCH)(PySe)$_2$] (**9**) in $CDCl_3$ at -30 °C.

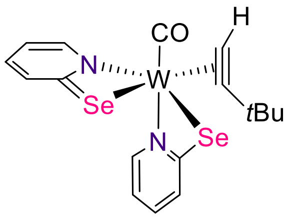

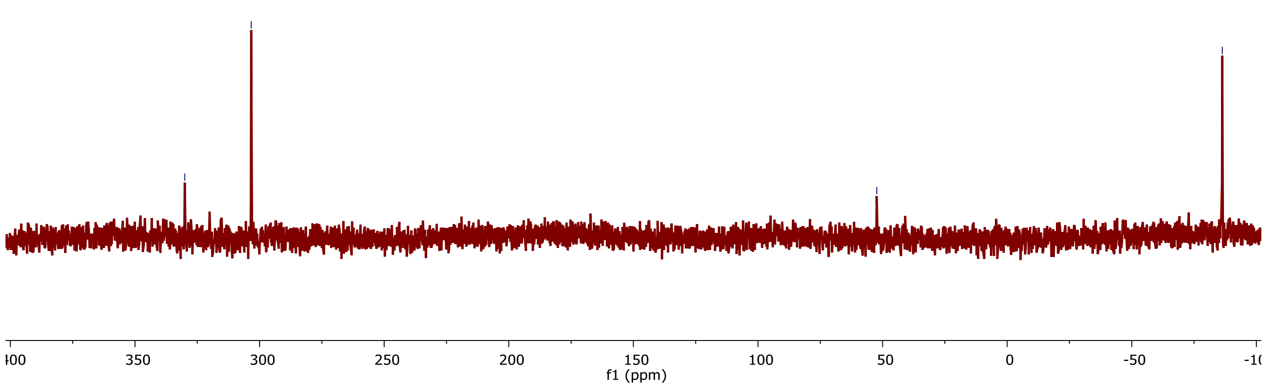

Figure S38. ^{77}Se NMR spectrum of [W(CO)(*t*Bu-CCH)(PySe)$_2$] (**9**) in $CDCl_3$ at -20 °C.

Computational Details

The computational results presented have been achieved using the Vienna Scientific Cluster (VSC). Calculations were performed using the GAUSSIAN 09 software package[9] and the PBE0 functional without symmetry constraints. That functional uses a hybrid generalized gradient approximation (GGA), including 25% mixture of Hartree-Fock[10] exchange with DFT[11] exchange-correlation, given by Perdew, Burke and Ernzerhof functional (PBE).[12,13] The optimized geometries were obtained with the Stuttgart/Dresden ECP (SDD) basis set[14-16] to describe the electrons of the tungsten atoms. For all other atoms, a standard 6-31G** basis set was employed.[17-22] A Natural Population Analysis (NPA)[23-29] was used to study the electronic structure and bonding of the optimized species.

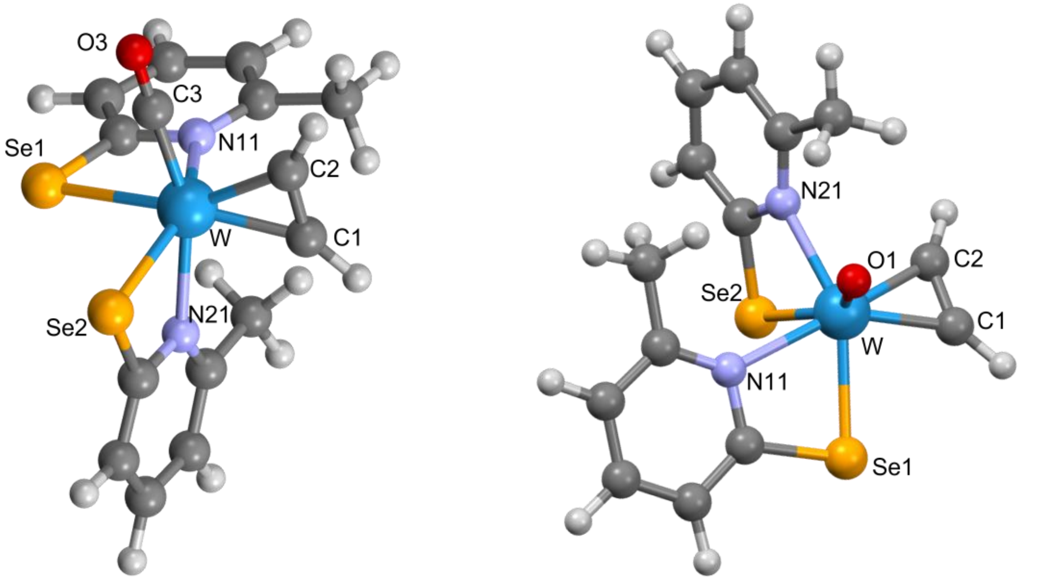

Figure S39. Optimized structures for **3** (left) and **4** (right).

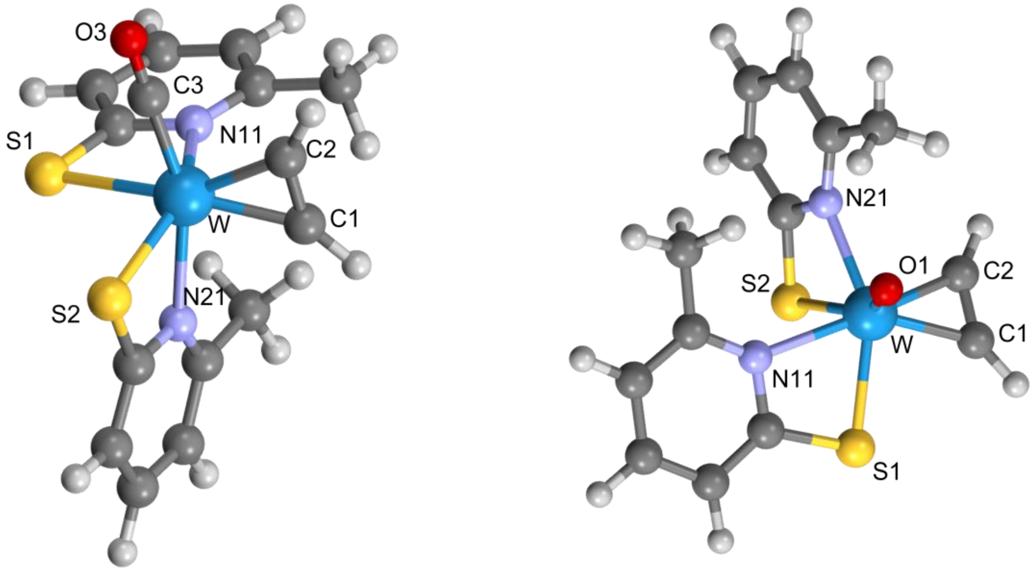

Figure S40. Optimized structures for $[W(CO)(C_2H_2)(6\text{-}MePyS)_2]$ (left) and $[WO(C_2H_2)(6\text{-}MePyS)_2]$ (right).[30]

Table S25. Selected calculated bond lengths (Å) in **3**, **4**, [W(CO)(C_2H_2)(6-MePyS)$_2$], and [WO(C_2H_2)(6-MePyS)$_2$].

	[W(CO)(C_2H_2)(6-MePy**E**)$_2$]		[WO(C_2H_2)(6-MePy**E**)$_2$]	
	**E** = Se (**3**)	**E** = S[30]	**E** = Se (**4**)	**E** = S[30]
W1-C1	2.029	2.027	2.083	2.083
W1-C2	2.041	2.043	2.087	2.088
W1-C3	1.984	1.985	-	-
W1-N11	2.237	2.221	2.327	2.307
W1-N21	2.292	2.279	2.238	2.224
W1-E1	2.702	2.602	2.554	2.661
W1-E2	2.530	2.419	2.760	2.443
W1-O1	-	-	1.722	1.720
C1-C2	1.320	1.319	1.291	1.290
C3-O3	1.158	1.158	-	-

XYZ coordinates of [W(CO)(C_2H_2)(6-MePyS)$_2$]

74	4.460209000	4.869361000	16.277916000
6	2.951108000	3.667426000	15.655870000
1	2.629747000	2.677477000	15.356795000
6	2.481773000	4.895504000	15.768063000
1	1.589828000	5.493225000	15.638304000
6	3.862703000	6.751708000	16.474811000
8	3.492480000	7.845417000	16.562838000

16	6.556227000	5.641904000	17.612853000
7	4.412306000	4.520499000	18.471089000
6	5.626186000	4.959116000	18.898852000
6	6.010210000	4.842418000	20.239381000
1	6.988937000	5.197480000	20.541256000
6	5.117189000	4.286674000	21.136322000
1	5.384077000	4.186699000	22.184663000
6	3.863123000	3.867286000	20.689860000
1	3.138327000	3.445763000	21.377483000
6	3.526314000	3.996685000	19.347312000
6	2.182439000	3.576662000	18.849012000
1	1.664224000	4.418567000	18.378905000
1	1.570819000	3.200087000	19.671254000
1	2.266257000	2.795199000	18.086429000
16	5.806426000	5.158919000	14.289117000
7	5.813630000	3.096287000	15.808904000
6	6.405883000	3.559864000	14.685778000
6	7.371207000	2.822470000	14.001857000
1	7.833632000	3.229512000	13.109998000
6	7.699617000	1.571185000	14.499959000
1	8.439215000	0.959344000	13.990877000
6	7.080807000	1.097931000	15.655783000
1	7.329125000	0.123516000	16.062214000
6	6.137323000	1.891180000	16.304286000

6	5.468569000	1.455502000	17.568878000
1	5.840472000	2.041109000	18.416146000
1	5.658492000	0.399009000	17.769337000
1	4.389223000	1.619728000	17.509086000

XYZ coordinates of [WO(C_2H_2)(6-MePyS)$_2$]

74	4.213408000	16.420428000	1.485791000
8	4.982676000	16.386147000	-0.052706000
6	2.812190000	17.944797000	1.260717000
1	1.833266000	18.141847000	0.847220000
6	3.821196000	18.428517000	1.903256000
1	4.229719000	19.308709000	2.379176000
16	2.195684000	15.046101000	1.388407000
7	4.575550000	14.148181000	1.653918000
6	3.305110000	13.705030000	1.565561000
6	2.989879000	12.348621000	1.604619000
1	1.956769000	12.027860000	1.537523000
6	4.038721000	11.449649000	1.717022000
1	3.839502000	10.382035000	1.747166000
6	5.350885000	11.914656000	1.779067000
1	6.183786000	11.224162000	1.853873000
6	5.600113000	13.284253000	1.743653000
6	6.986768000	13.837854000	1.804215000
1	7.130112000	14.583794000	1.017847000

1	7.725642000	13.043726000	1.679780000
1	7.165519000	14.328177000	2.766567000
16	4.276370000	16.025111000	4.116589000
7	6.107249000	16.909612000	2.544271000
6	5.882736000	16.583086000	3.843316000
6	6.899365000	16.722069000	4.800476000
1	6.699035000	16.448042000	5.829786000
6	8.124090000	17.212599000	4.393066000
1	8.930363000	17.330586000	5.112011000
6	8.323720000	17.571964000	3.056948000
1	9.271905000	17.978963000	2.724035000
6	7.291503000	17.420082000	2.139755000
6	7.450229000	17.827702000	0.710930000
1	7.293235000	16.982603000	0.035596000
1	8.442777000	18.251112000	0.545000000
1	6.695092000	18.571632000	0.438334000

XYZ coordinates of **3**

74	2.759094000	3.167718000	16.326336000
6	4.246974000	4.388566000	15.684958000
1	4.554188000	5.375192000	15.360350000
6	4.738813000	3.171798000	15.828175000
1	5.642955000	2.589018000	15.715073000
6	3.387126000	1.301829000	16.573632000

8	3.780674000	0.218337000	16.686198000
34	0.573419000	2.304138000	17.660629000
7	2.780958000	3.548408000	18.530770000
6	1.591321000	3.096500000	19.007646000
6	1.240565000	3.233084000	20.352183000
1	0.277310000	2.865045000	20.686456000
6	2.142988000	3.824508000	21.216986000
1	1.901000000	3.942547000	22.269430000
6	3.376009000	4.252549000	20.726838000
1	4.112971000	4.699382000	21.384970000
6	3.680431000	4.100572000	19.378050000
6	5.010675000	4.527242000	18.850066000
1	5.535770000	3.679838000	18.397885000
1	5.626018000	4.934804000	19.654581000
1	4.908604000	5.284961000	18.066543000
34	1.405958000	2.773219000	14.224783000
7	1.376386000	4.921969000	15.811524000
6	0.776529000	4.494874000	14.678267000
6	-0.175906000	5.258251000	14.008719000
1	-0.641574000	4.874980000	13.108011000
6	-0.490746000	6.504586000	14.527276000
1	-1.221244000	7.135902000	14.028964000
6	0.135769000	6.944820000	15.690791000
1	-0.096607000	7.914988000	16.116523000

6	1.067346000	6.125788000	16.324635000
6	1.737370000	6.538966000	17.595457000
1	1.340900000	5.963077000	18.438140000
1	1.574706000	7.599996000	17.796389000
1	2.811795000	6.344653000	17.543785000

YXZ coordinates of **4**

74	0.943950000	2.365531000	1.508715000
6	-0.437797000	0.807782000	1.565362000
1	-1.145810000	0.271139000	0.949656000
6	0.084838000	0.917050000	2.740939000
1	0.090446000	0.521073000	3.746670000
8	2.305283000	1.634948000	0.747683000
34	-0.668872000	3.197994000	-0.288012000
7	1.590453000	4.364521000	0.507881000
6	0.599199000	4.589460000	-0.376829000
6	0.585371000	5.700222000	-1.213665000
1	-0.234510000	5.849270000	-1.906782000
6	1.652012000	6.582085000	-1.132002000
1	1.683566000	7.461880000	-1.768691000
6	2.690592000	6.327863000	-0.240345000
1	3.542527000	6.995616000	-0.172172000
6	2.641162000	5.200648000	0.576736000
6	3.735197000	4.885670000	1.544243000

1	3.406829000	5.070982000	2.572120000
1	4.613450000	5.503876000	1.347575000
1	4.015632000	3.832175000	1.466035000
34	-0.122438000	4.267229000	3.200904000
7	2.098863000	2.774079000	3.381162000
6	1.449726000	3.757736000	4.053632000
6	1.959779000	4.268895000	5.252880000
1	1.423197000	5.060876000	5.762190000
6	3.132806000	3.737226000	5.752053000
1	3.553969000	4.114697000	6.679916000
6	3.766697000	2.697849000	5.068331000
1	4.673798000	2.246842000	5.455042000
6	3.226916000	2.217960000	3.880524000
6	3.861612000	1.077136000	3.152988000
1	3.145139000	0.260886000	3.018390000
1	4.722515000	0.706441000	3.712806000
1	4.174679000	1.367099000	2.146719000

References

(1) Vidovič, C.; Peschel, L. M.; Buchsteiner, M.; Belaj, F.; Mösch-Zanetti, N. C. Structural Mimics of Acetylene Hydratase: Tungsten Complexes Capable of Intramolecular Nucleophilic Attack on Acetylene. *Chem. Eur. J.* **2019**, *25* (63), 14267–14272.

(2) *APEX3*, Bruker AXS, Inc. 2018.

(3) *APEX2*, Bruker AXS, Inc. 2012.

(4) *SADABS*, Bruker AXS, Inc. 2016.

(5) Sheldrick, G. M. SHELXT - integrated space-group and crystal-structure determination. *Acta Cryst.* **2015**, A*71*, 3–8.

(6) Sheldrick, G. M. Crystal structure refinement with SHELXL. *Acta Cryst.* **2015**, C*71*, 3–8.

(7) Dolomanov, O. V.; Bourhis, L. J.; Gildea, R. J.; Howard, J. A. K.; Puschmann, H. OLEX2 : a complete structure solution, refinement and analysis program. *J. Appl. Crystallogr.* **2009**, *42* (2), 339–341.

(8) Sheldrick, G. M. A short history of SHELX. *Acta Cryst.* **2008**, *A64,* 112–122.

(9) Gaussian 09, Revision A.01, M. J. Frisch, G. W. Trucks, H. B. Schlegel, G. E. Scuseria, M. A. Robb, J. R. Cheeseman, G. Scalmani, V. Barone, G. A. Petersson, H. Nakatsuji, X. Li, M. Caricato, A. Marenich, J. Bloino, B. G. Janesko, R. Gomperts, B. Mennucci, H. P. Hratchian, J. V. Ortiz, A. F. Izmaylov, J. L. Sonnenberg, D. Williams-Young, F. Ding, F. Lipparini, F. Egidi, J. Goings, B. Peng, A. Petrone, T. Henderson, D. Ranasinghe, V. G. Zakrzewski, J. Gao, N. Rega, G. Zheng, W. Liang, M. Hada, M. Ehara, K. Toyota, R. Fukuda, J. Hasegawa, M. Ishida, T. Nakajima,

Y. Honda, O. Kitao, H. Nakai, T. Vreven, K. Throssell, J. A. Montgomery, Jr., J. E. Peralta, F. Ogliaro, M. Bearpark, J. J. Heyd, E. Brothers, K. N. Kudin, V. N. Staroverov, T. Keith, R. Kobayashi, J. Normand, K. Raghavachari, A. Rendell, J. C. Burant, S. S. Iyengar, J. Tomasi, M. Cossi, J. M. Millam, M. Klene, C. Adamo, R. Cammi, J. W. Ochterski, R. L. Martin, K. Morokuma, O. Farkas, J. B. Foresman, and D. J. Fox, Gaussian, Inc., Wallingford CT, 2016.

(10) Hehre, W. J. Ab initio molecular orbital theory. *Acc. Chem. Res.* **1976**, *9* (11), 399–406.

(11) Parr, R. G. Density Functional Theory. *Annu. Rev. Phys. Chem.* **1983**, *34* (1), 631–656.

(12) Perdew, J. P. Density-functional approximation for the correlation energy of the inhomogeneous electron gas. *Phys. Rev. B: Condens. Matter* **1986**, *33* (12), 8822–8824.

(13) Perdew, J. P.; Burke, K.; Ernzerhof, M. Generalized Gradient Approximation Made Simple. *Phys. Rev. Lett.* **1996**, *77* (18), 3865–3868.

(14) Leininger, T.; Nicklass, A.; Stoll, H.; Dolg, M.; Schwerdtfeger, P. The accuracy of the pseudopotential approximation. II. A comparison of various core sizes for indium pseudopotentials in calculations for spectroscopic constants of InH, InF, and InCl. *J. Chem. Phys.* **1996**, *105* (3), 1052–1059.

(15) Küchle, W.; Dolg, M.; Stoll, H.; Preuss, H. Energy-adjusted pseudopotentials for the actinides. Parameter sets and test calculations for thorium and thorium monoxide. *J. Chem. Phys.* **1994**, *100* (10), 7535–7542.

(16) Häussermann, U.; Dolg, M.; Stoll, H.; Preuss, H.; Schwerdtfeger, P.; Pitzer, R. M. Accuracy of energy-adjusted quasirelativistic ab initio pseudopotentials. *Mol. Phys.* **1993**, *78* (5), 1211–1224.

(17) Curtiss, L. A.; McGrath, M. P.; Blaudeau, J.-P.; Davis, N. E.; Binning, R. C.; Radom, L. Extension of Gaussian-2 theory to molecules containing third-row atoms Ga–Kr. *J. Chem. Phys.* **1995**, *103* (14), 6104–6113.

(18) McGrath, M. P.; Radom, L. Extension of Gaussian-1 (G1) theory to bromine-containing molecules. *J. Chem. Phys.* **1991**, *94* (1), 511–516.

(19) Raghavachari, K.; Trucks, G. W. Highly correlated systems. Excitation energies of first row transition metals Sc–Cu. *J. Chem. Phys.* **1989**, *91* (2), 1062–1065.

(20) Hay, P. J. Gaussian basis sets for molecular calculations. The representation of 3 d orbitals in transition-metal atoms. *J. Chem. Phys.* **1977**, *66* (10), 4377–4384.

(21) Krishnan, R.; Binkley, J. S.; Seeger, R.; Pople, J. A. Self-consistent molecular orbital methods. XX. A basis set for correlated wave functions. *J. Chem. Phys.* **1980**, *72* (1), 650–654.

(22) McLean, A. D.; Chandler, G. S. Contracted Gaussian basis sets for molecular calculations. I. Second row atoms, Z =11–18. *J. Chem. Phys.* **1980**, *72* (10), 5639–5648.

(23) Reed, A. E.; Curtiss, L. A.; Weinhold, F. Intermolecular interactions from a natural bond orbital, donor-acceptor viewpoint. *Chem. Rev.* **1988**, *88* (6), 899–926.

(24) Carpenter, J. E. Extension of Lewis structure concepts to open-shell and excited-state. PhD Thesis, University of Wisconsin, Madison, WI.

(25) Foster, J. P.; Weinhold, F. Natural hybrid orbitals. *J. Am. Chem. Soc.* **1980**, *102* (24), 7211–7218.

(26) Reed, A. E.; Weinhold, F. Natural bond orbital analysis of near-Hartree–Fock water dimer. *J. Chem. Phys.* **1983**, *78* (6), 4066–4073.

(27) Reed, A. E.; Weinhold, F. Natural localized molecular orbitals. *J. Chem. Phys.* **1985**, *83* (4), 1736–1740.

(28) Reed, A. E.; Weinstock, R. B.; Weinhold, F. Natural population analysis. *J. Chem. Phys.* **1985**, *83* (2), 735–746.

(29) Carpenter, J. E.; Weinhold, F. Analysis of the geometry of the hydroxymethyl radical by the "different hybrids for different spins" natural bond orbital procedure. *J. Mol. Struct.* **1988**, *169*, 41–62.

(30) Ehweiner, M. A.; Belaj, F.; Kirchner, K.; Mösch-Zanetti, N. C. Synthesis and Reactivity of a Bioinspired Molybdenum(IV) Acetylene Complex. *Organometallics* **2021**, *40* (15), 2576–2583.
